# Single coronary artery presenting dilated cardiomyopathy and hyperlipidemia with the *SCN5A* and *APOA5* gene mutation: A case report and review of the literature

**DOI:** 10.3389/fcvm.2023.1113886

**Published:** 2023-05-22

**Authors:** Xiaoxia Hu, Jing Kong, Tingting Niu, Liang Chen, Jingjing Yang

**Affiliations:** ^1^ Department of Cardiology, Qilu Hospital of Shandong University, Jinan, Shandong, China; ^2^ Department of Medical Technology, Jinan Vocational College of Nursing, Jinan, Shandong, China; ^3^ Department of Emergency Medicine, Qilu Hospital of Shandong University, Jinan, Shandong, China

**Keywords:** *SCN5A*, single coronary artery, dilated cardiomyopathy, genetic mutation, hyperlipidemia

## Abstract

We present a 55-year-old man with chest tightness and dyspnoea after activity lasting for 2 months who was diagnosed with single coronary artery (SCA) and presented with dilated cardiomyopathy (DCM) with the c.1858C > T mutation in the *SCN5A* gene. The computed tomography coronary angiogram (CTCA) showed congenital absence of the right coronary artery (RCA), and the right heart was nourished by the left coronary artery branch with no apparent stenosis. Transthoracic echocardiography (TTE) revealed enlargement of the left heart and cardiomyopathy. Cardiac magnetic resonance imaging (CMR) revealed DCM. Genetic testing showed that the c.1858C > T variant of the *SCN5A* gene could lead to Brugada syndrome and DCM. SCA is a rare congenital anomaly of the coronary anatomy, and this case reported as SCA accompanied by DCM is even rarer. We present a rare case of a 55-year-old man with DCM with the c.1858C > T (p. Arg620Cys)/c.1008G > A (p.(Pro336=) variant of the *SCN5A* gene, congenital absence of RCA, and c.990_993delAACA (p. Asp332Valfs*5) variant of the *APOA5* gene. To our knowledge, this is the first report of DCM combined with the *SCN5A* gene mutation in SCA after searching the PubMed, CNKI and Wanfang databases.

## 1. Introduction

Coronary artery abnormalities include abnormal number and origin, while single coronary artery (SCA) is relatively rare, accounting for 0.031% in coronary angiography (1, 2) and 0.024%–0.066% in the general population (3, 4). It is unclear whether SCA is an isolated congenital heart disease or is associated with other congenital abnormalities. Dilated cardiomyopathy (DCM) is currently defined by the presence of left ventricular or biventricular dilatation and systolic dysfunction in the absence of abnormal loading conditions (hypertension, valve disease) or coronary artery disease sufficient to cause global systolic impairment (5, 6). The causes of DCM are heterogeneous (7), and we believe this is the result of genetic predisposition interacting with extrinsic or environmental factors (8, 9). The *SCN5A* gene mutation is associated with a range of clinical diseases. Here, we present a rare case of SCA with DCM accompanied by the *SCN5A* gene mutation.

## 2. Case presentation

**Figure 1:**
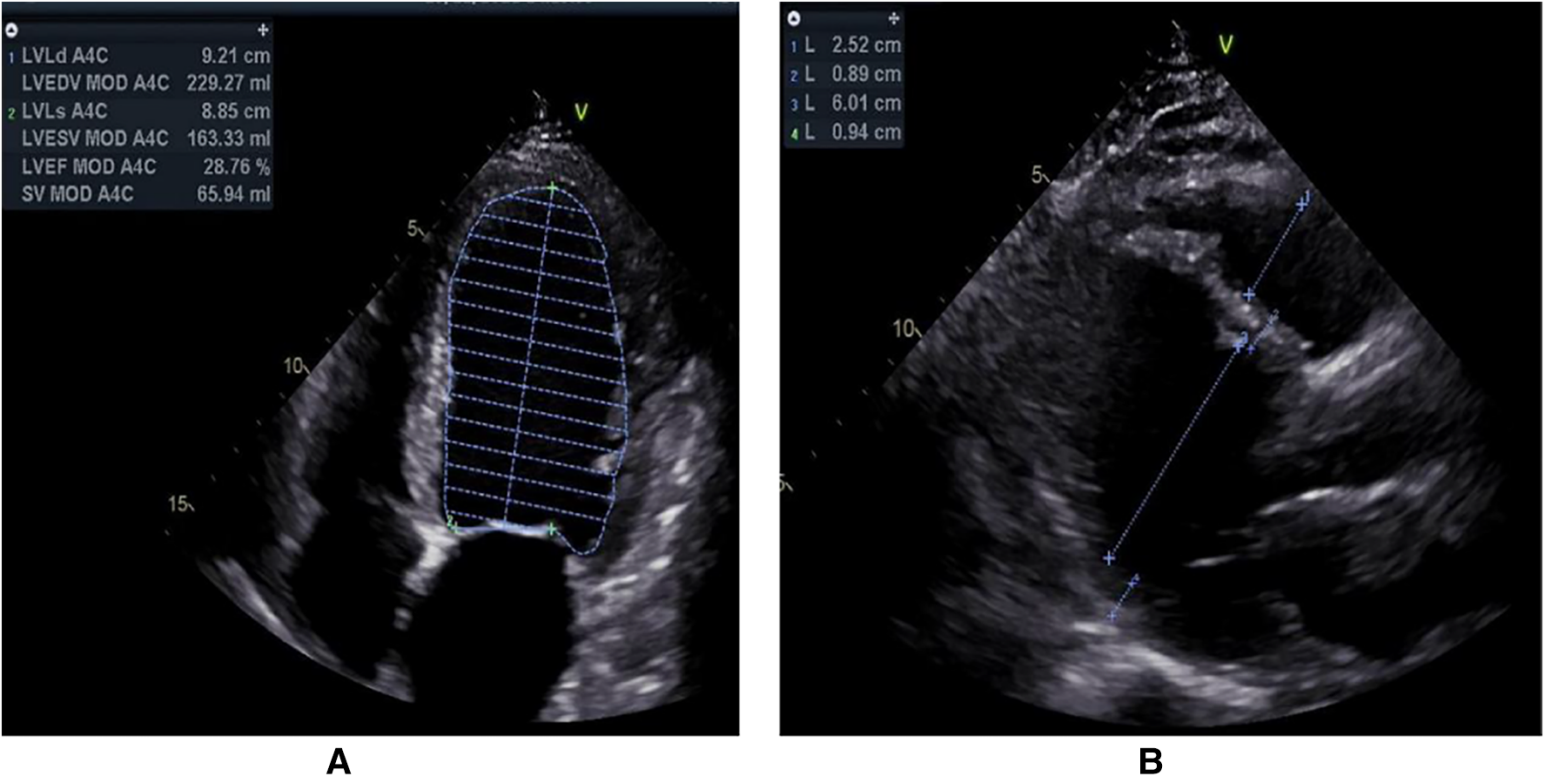
Transthoracic echocardiography was routinely examined from left parasternal long-axis view (**A**) and apical four-chamber view (**B**).

A 55-year-old male was admitted to our hospital because of dyspnoea. That day, he felt dyspnoea after 200 metres of flat walking. Emergency medical services were called, and he was transported to the emergency department at our hospital. On evaluation, the systolic blood pressure was 116/91 mmHg, the pulse was 115 beats per minute, the respiratory rate was 17 breaths per minute, and the oxygen saturation was 100%. An electrocardiogram (ECG) showed sinus bradycardia with T-wave inversions and premature ventricular contractions (Supplementary Figure S1). On arrival, the patient reported paroxysmal dyspnoea. He had multiple similar episodes during the previous 20 days, without fever, cough, vomiting or diarrhoea. The symptoms were usually provoked by physical exertion, mental stress or intense emotion. Evaluations at other hospitals showed chronic bronchitis and emphysema, an enlarged left ventricle and decreased cardiac function, and further treatment was recommended. The patient had no other illnesses. His family history was unremarkable. He was a farmer. He drank alcohol occasionally, and had smoked in the past 30 years, and did not use illicit drugs or herbal preparations. There were no recent exposures to ill persons. Physical examination showed oedema of both lower limbs, while the other limbs were normal. Levels of sodium, chloride, carbon dioxide, D-dimer, magnesium and tests of liver function and renal function were normal. Troponin I and serum NT-proBNP were rising. A 24-h Holter monitor revealed occasional atrial premature beats, frequent multiple ventricular premature beats and ST-T changes. Transthoracic echocardiography (TTE) showed decreased systolic function (LVEF = 29%), an enlarged left ventricle (LVEDD = 60 mm), cardiomyopathy, moderate mitral regurgitation and moderate pulmonary hypertension (Figure 1). There was no family history of cardiovascular disease. Drugs improving microcirculation and cardiac function were administered, and he was then admitted to our ward. Laboratory tests were conducted, and the results are shown in Table 1.

**Table 1:** Laboratory data.

Variable	Reference range in adults	At admission	At discharge
Hemoglobin (g/L)	130–175	154	161
White-cell count (*10^12^/L)	3.5–9.5	9.04	5.22
Platelet count (*10^9^/L)	125–350	191	194
D-Dimer (ug/ml)	<0.50	0.80	0.25
Potassium (mmol/L)	3.60–5.0	4.06	4.05
Sodium (mmol/L)	137–145	141.0	141
Creatinine (umol/L)	58–133	69	92
BUN (mmol/L)	3.2–7.1	20	5.70
NT-proBNP (pg/ml)	≥900	2243.00	1367
Hs-CTNI (ng/L)	0–17.5	36.82	14.42
LDL-C (mmol/L)	1.00–3.37		3.71
CHO (mmol/L)	2.80–6.00		5.63
TG (mmol/L)	0.30–1.70		2.44
GLU (mmol/L)	3.90–6.10		4.73
T3 (pmol/L)	2.30–6.30		5.26
T4 (pmol/L)	10.3–24.5		19.53
TSH (UIU/ml)	0.35–5.5		1.22

BUN, blood urea nitrogen; NT-proBNP, N-terminal pro-B-type natriuretic peptide; Hs-CTNI, hypersensitive cardiac troponin I; LDL-C, Low-Density Lipoprotein Cholesterol; CHO, Cholesterol; TG, Triglyceride; GLU, glucose; T3, triiodothyronine; T4, tetraiodothyronine; TSH, thyroid stlmulating hormone. The variabkes were test using haematological samples. Reference values are affected by many variables, including the patient population and the laboratory methods used. The ranges used at qilu Hospital of Shandong university are for adults who are not pregnant and do not have medical conditions that could affect the results. They may therefore not be appropriate for all patients.

**Figure 2:**
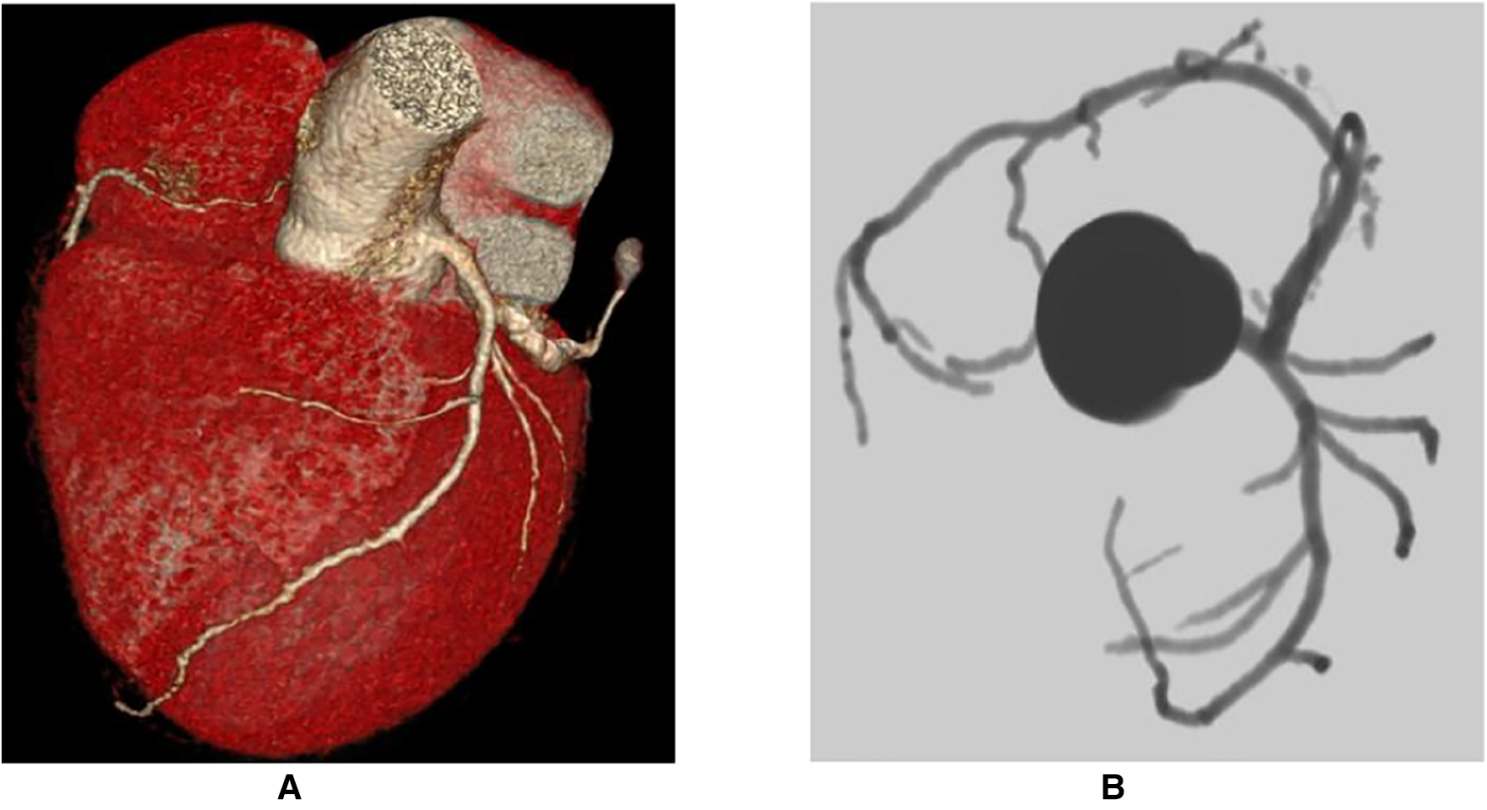
Computed tomography coronary angiogram. (**A**) Course of the LCx on the posterior atrioventricular groove and continuation of its course in the RCA territory along with take-off of the posterior descending artery. (**B**) Absence of take-off of the RCA from the right coronary sinus of Valsalva (yellow arrow) and normal origin of the LMS which bifurcates into the LAD and LCX. LAD, left anterior descending artery; LCX, left circumflex artery; LMS, left main stem artery; RCA, right coronary artery.

**Figure 3:**
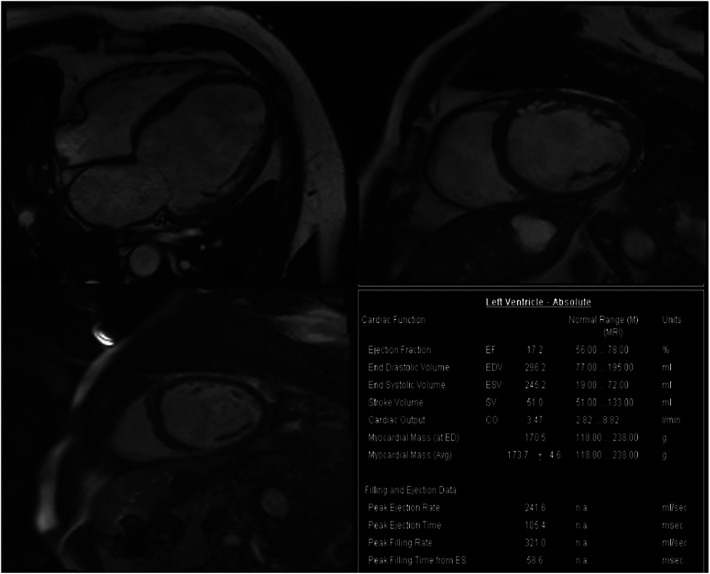
Cardiac magnetic resonance imaging showed DCM. CMR detected dilation of the left ventricle (LVEDV = 296.2 ml), reduction of cardiac systolic function (CO = 3.47l/min, LVEF = 17.2%) and increase of left ventricular mass (LVM = 170.5 g), no signs of storage disease or inflammation. CMR, Cardiac magnetic resonance imaging; LVEDV, left ventricular end-diastolic volume; LVEF, left ventricular ejection fraction; CO, cardiac output.

Based on the above clinical examination, computed tomography coronary angiogram (CTCA) was performed for the patient to rule out atherosclerotic coronary artery disease. To our surprise, the patient had a very rare SCA, a congenital abnormality of the coronary artery system that may provide low perfusion to the entire heart muscle, which causes chest pain, angina or dyspnoea. CTCA showed that a coronary artery from Valsalva's left sinus was divided into the left anterior descending branch (LAD) and the left circumflex branch (LCX). The distal end of the LCX continued its course beyond the crux into the atrioventricular groove, supplying the right atrium and right ventricle with a superdominant LCX without stenosis and a calcium score of zero Agatston units (Figure 2). Because no intervention would be appropriate in the absence of significant coronary artery stenosis in CTCA, we decided not to perform invasive coronary angiography. To identify the cause of heart failure, we performed cardiovascular magnetic resonance (CMR) on the patient. CMR showed reduced left ventricular systolic function (LVEF = 17.2%), left ventricular enlargement (LVEDV = 296.2 ml), thinning of the myocardium and abnormal delayed reinforcement in the basal segment of the ventricular septum, which was consistent with the diagnosis of DCM (Figure 3). Although improved cardiac imaging techniques have made endomyocardial biopsy (EMB) less necessary, it has traditionally been used to confirm the aetiology in some forms of DCM. However, EMB is no longer frequently performed and was not conducted in this case.

**Figure 4:**
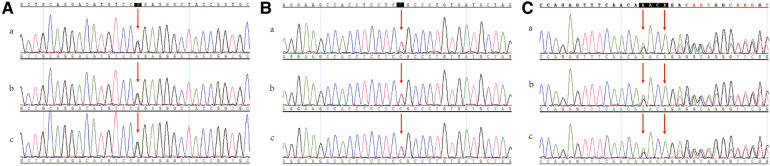
Sanger sequencing data. (**A**) *SCN5A*; NM_198056.2; c.1858C > T; p.Arg620Cys. (**B**) *SCN5A*; NM_198056.2; c.1008G > A; p.(Pro336=). (**C**) *APOA5*; NM_052968.4; c.990_993delAACA; p.Asp332Valfs*5. (a) proband (b) the son of proband (c) the daughter of proband.

We suspected that the mutation of a certain gene caused the patient to have SCA and DCM, therefore, we arranged a clinical gene test of the whole exon. Genetic screening showed the *SCN5A*; NM_198056.2:c.1858C > T (p. Arg620Cys) mutation (10–13) and the pathogenicity of this variant has been reported; *SCN5A*; NM_ 198056.2:c.1008 g > a [P. (pro336=)] mutation and there is no report on the pathogenicity of this variant. Sanger sequencing result is shown in Figure 4 and NSG data is shown in Supplementary Table S1. The *SCN5A* C.1858C > T (P. arg620Cys) mutation may be associated with DCM in this patient. At the same time, *APOA5*; NM_052968.4:c.990_993delAACA (p. Asp332Valfs*5) was mutated in this patient, and its pathogenicity has been reported. According to ACMG Guidelines, this variant is considered a suspected pathogenic variant (14). The *APOA5* gene mutation results in hypertriglyceridemia or hyperlipoproteinemia type 5, which was in line with the diagnosis of hypertriglyceridemia based on the patient's blood test. The relationship between SCA and DCM was not elucidated by genetic testing. There was no previous report on the absence of a RCA with DCM. According to findings of CTCA, TTE, CMR and other test results, we considered that the occurrence of DCM in this patient may not due to the absence of the RCA. Rather, we supposed that a gene mutation may cause the SCA and DCM.

Therefore, we performed family verification on the two children of the patient. The children of the proband both carried the *SCN5A*: c.1858C > T(p. Arg620Cys)/c.1008G > A [p.(Pro336=)] variant and *APOA5*: c.990_993delAACA (p. Asp332Valfs*5) variant.

## 3. Discussion

This is a rare case of congenital absence of the RCA in a patient with a DCM-related gene mutation and hyperlipidemia. Our patient had tested positive for a gene variant known to cause DCM and had 2 months of chest tightness and dyspnoea after activity along with hyperlipidemia. The son and daughter of the proband also tested positive for the same mutation. We did not perform a full pedigree of the family since other relatives refused to be tested for the relevant mutations.

### 3.1. Genetic DCM

The prevalence of DCM and of genetically mediated DCM is not fully known because of geographic variations, patient selection and changes in the diagnostic criteria (15–18). The European Society of Cardiology Working Group on Myocardial and Pericardial Diseases presented an update of the existing classification scheme of cardiomyopathies in 2008. They grouped the cardiomyopathies into specific morphological and functional phenotypes, including hypertrophic cardiomyopathies, DCM, arrhythmogenic right ventricular cardiomyopathies (ARVC), restrictive cardiomyopathies and unclassified cardiomyopathies. Each phenotype was then subclassified into genetic and nongenetic forms, but overlaps exist between the two groups (6, 8). Meanwhile, studies have shown that 20%–50% of non-ischemia cardiomyopathy patients have a family history (19). All of these findings suggest that genetic factors play an important role in the pathogenesis of cardiomyopathy.

Familial patterns or genetic causes have been identified in up to 35% of cases of idiopathic DCM (20). The genetic causes of DCM are diverse. An autosomal dominant trait is the most common, while autosomal recessive, X-linked and mitochondrial inheritance patterns are less common (21). *TTN* and *LMNA* are the main genes associated with the predominant cardiac phenotype, accounting for up to 25% and 5% of all cases of autosomal dominant DCM, respectively (22). *RBM20* and *DES* are also common in clinical practice (23, 24). Other genetic causes of a predominant DCM phenotype include mutations in sarcomere genes, Z-disc protein-encoding genes, genes encoding desmosomal proteins and genes associated with ion channel function (10–29).

The *SCN5A* gene is associated with ion channel function caused by DCM. With the development of gene detection technology, an increasing number of individual genes have been associated with inheritance in familial DCM cases. Cheng Shen et al. performed a cross-sectional study in Chinese patients with sporadic DCM and suggested that *MYBPC3*, *SCN5A*, *MYH7*, *MYPN* and *LDB3* are the major genes hosting the at-risk genomic variants of sporadic DCM (30).

### 3.2. *SCN5A* gene mutation-related clinical diseases

**Figure 5:**
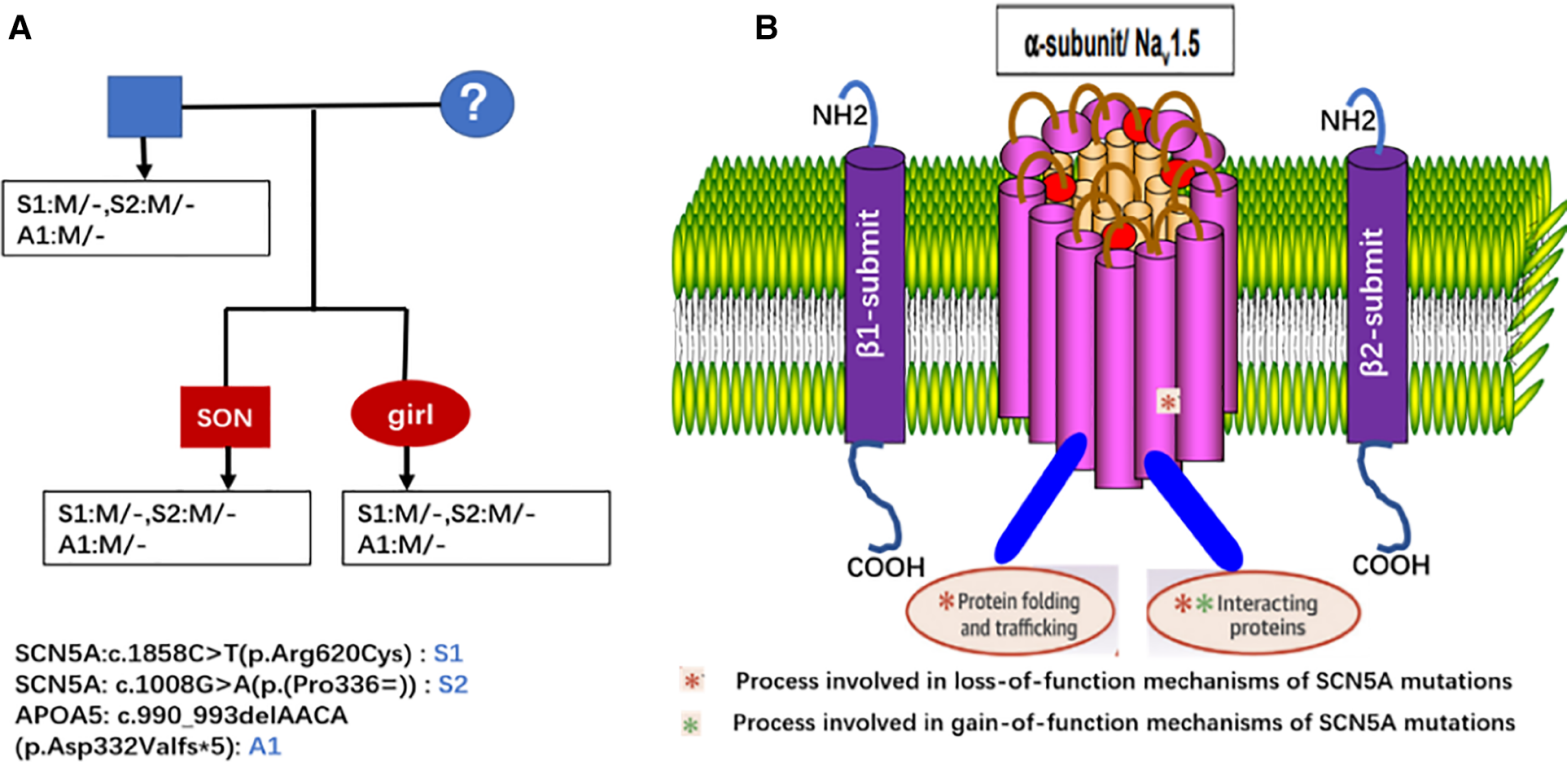
Genetic test results of the proband and his children, both the proband and his children had the same three genes mutation and were heterozygous. (**A**) Molecular structure of the *SCN5A* translated into the a-subunit (Nav1.5) of the cardiac sodium channel. (**B**) Nav1.5 consists of an N-terminus, C-terminus and 4 structurally homologous domains.

The *SCN5A* gene, located on chromosome 3p21 with 28 exons, encodes the alpha subunit of the main sodium channel Nav1.5, which enables the rapid influx of Na^+^ ions (INa) (19). This alpha subunit is expressed in human cardiomyocytes as well as in many other tissues and cells, such as embryonic and denervated skeletal muscle cells in the brain, interstitial cells of Cajal in the human jejunum and colon and smooth muscle cells (31–36). Some studies have shown that the *SCN5A* splice variant is also expressed in macrophages, where it activates innate immune signalling for antiviral defence (37–39). Voltage-gated Na^+^ channels are crucial in the excitation and propagation of electrical impulses in cardiomyocytes (40). Nav1.5 interacts with several proteins, including ancillary *β*-subunits, fascia adherens junctions, desmosomes, gap junctions and intracellular proteins that regulate the gain-of-function and loss-of-function of Nav1.5 proteins (Figure 5) (41). *SCN5A* gene mutations are associated with a clinical spectrum, including Brugada syndrome, long-QT syndrome, progressive cardiac conduction disease, sinus node dysfunction, atrial fibrillation, DCM, multifocal ectopic Purkinje-related premature contractions, irritable bowel syndrome (IBS) and other gastrointestinal disorders, such as chronic idiopathic intestinal pseudo-obstruction (32, 34, 41, 42).

### 3.3. *SCN5A* gene mutation and DCM

The genetic background of patients with DCM is complex: 7% have a single heterozygous mutation, more than 38% have a compound heterozygous or combined mutation, and 12.8% have three or more mutations (43–45). Although many previous works strongly implied that the *SCN5A* gene mutation plays a critical role in the development of cardiomyopathy, the mechanisms remain controversial. Several mechanisms have been postulated. *SCN5A* gene mutations related to tachyarrhythmia or other conduction abnormalities induce DCM, especially for some patients with a long history of arrhythmia (41, 46–49). A primary disruption of Nav1.5 can affect cellular pH and Ca^2+^ homeostasis and result in a DCM phenotype (42, 50–52). The SCN5A channel mutation disrupts the sodium channel domain to target the appropriate cytoskeletal components, such as syntrophins and dystrophins (48, 53–57). In addition, the environment and common gene variants may act together with *SCN5A* mutations to cause DCM (42, 52, 58, 59).

The proband and offspring carried the same three mutations, but they have not shown signs of DCM, and we will continue to follow the family.

### 3.4. SCA: absence of RCA

In 1979, Lipton et al. defined and classified SCA, which can be divided into 9 types according to origin and anatomical process. The cause of SCA is still unknown (60). Although isolated reports indicate that specific coronary abnormalities occur in families, no clear pattern of coronary inheritance has been found in humans (61). We reported a rare L-I pattern of a SCA according to the Lipton classification and summarized a review of identical types of SCA literature, in which the RCA was absent and the LCX was markedly dominant and nourished the right ventricle and atrium beyond the atrioventricular groove. A careful review of the literature revealed 59 cases with a similar anomalous coronary origin and pattern, including demographic characteristics, symptoms, complications, diagnosis and treatment strategy (Table 2) (62–112). Of these 59 patients, 22 were male and 37 were female, and their mean age was 56 years (range 30–87 years).

**Table 2:** Summary characteristics of L-1 of SCA.

Case	Author/Year	Age/Sex	Coronary atherosclerotic heart disease	Arrhythmia/Cardiomyopathy/Congenital heart disease	Treatment	Hyperlipidemia	Symptom	CT/Coronary angiography/Autopsy	Associated conditions
1	Krumbh 1938^[58]^	44/F	NA	NA	NA	NA	NA	Autopsy	Pulmonary embolism
2	Smith 1950^[59]^	80/F	NA	NA	None	NA	Abdominal pain	Autopsy	None
3	LAURIE 1964^[60]^	60/M	NA	NA	None	NA	Weakness	Autopsy	Lymphoma
4	Lipton 1979^[61]^	39/M	None	None	NA	None	ATCP	CAG	None
5	Tavernarakis 1986^[62]^	57/M	Yes	Complete RBBB	Medicine	None	TCP	CAG	None
6	Sheth 1988^[63]^	60/M	None	None	NA	None	TCP	CAG	HP
7	Vrolix 1991^[64]^	51/M	Yes	None	CABG	None	TCP	CAG	IHD
8	Desmet 1992^[65]^	52/F	None	None	None	None	ATCP	CAG	None
9	Desmet 1992^[65]^	53/M	Yes	None	CABG	NA	NA	CAG	NA
10	Desmet 1992^[65]^	45/F	Yes	None	CABG	NA	NA	CAG	NA
11	Desmet 1992^[65]^	41/F	Yes	None	Medicine	NA	NA	CAG	NA
12	Desmet 1992^[65]^	64/M	Yes	None	Medicine	NA	NA	CAG	NA
13	Desmet 1992^[65]^	55/M	Yes	None	Medicine	NA	NA	CAG	NA
14	Shammas 2001^[66]^	44/F	Yes	None	NA	None	ATCP	CAG	None
15	Shammas 2001^[66]^	30/M	None	None	NA	Yes	Chest discomfort /dyspnea	CAG	Smoking
16	Turhan 2003^[67]^	52/M	None	None	NA	None	TCP	CAG	HP/Smoking
17	Asha 2003^[68]^	62/M	Yes	None	CABG	None	TCP	CAG	OMI
18	Chou 2004^[69]^	42/M	Yes	None	Medicine	Yes	TCP	CAG	Smoking
19	Yoshimoto 2004^[70]^	63/M	None	Af	NA	None	ATCP	CAG	None
20	Kang 2006^[71]^	43/M	None	None	None	None	ATCP	CAG	HP
21	Kunimasa 2007^[72]^	61/M	Yes	None	PCI	Yes	TCP	CT/CAG	HP/DM
22	Celik 2008^[73]^	57/M	Yes	None	Medicine	None	TCP	CAG	None
23	Tanawuttiwat 2009^[74]^	46/F	None	None	Medicine	Yes	TCP	CT/CAG	Smoking/HT
24	Datta 2010^[75]^	69/F	None	None	Medicine	None	TCP	CT/CAG	None
25	Choi 2010^[76]^	68/F	None	None	Medicine	None	Chest discomfort	CAG	None
26	Chung 2010^[77]^	77/F	Yes	None	PCI	None	TCP	CT/CAG	None
27	Ghaffari 2010^[78]^	65/F	None	Imcomplete RBBB	NA	None	Dyspnea	CT/CAG	HP/DM
28	Voyce 2010^[79]^	76/F	Yes/RVMI	None	PCI	None	None	CAG	None
29	Kalyani 2011^[80]^	35/M	None	None	NA	None	None	Autopsy	None
30	Kafkas 2011^[81]^	68/M	Yes/MI	IHD	PCI	Yes	Dyspnea	CAG	HP
31	Sonmez 2011^[82]^	63/F	Yes/MI	None	PCI	None	TCP	CAG	HP
32	Chen 2012^[83]^	75/F	Yes/MI	Af	Medicine	None	Chest tightness	CAG	HP/DM
33	Ma SH 2012^[84]^	39/M	Yes/MI	IHD	PCI	Yes	TCP	CT/CAG	Smoking
34	Devidutta 2013^[85]^	52/F	Yes	None	PCI	Yes	TCP	CAG	HP
35	Blaschke 2013^[86]^	59/F	None	None	None	None	TCP	NA	None
36	Pourbehi 2013^[87]^	47/M	Yes	None	PCI	None	ATCP	CAG	None
37	Ay Y 2014^[88]^	58/M	Yes	None	CABG	None	TCP	CAG	None
38	Mishra 2014^[89]^	55/M	Yes/MI	Complete RBBB/IHD	PCI	None	TCP	CT/CAG	Smoking
39	Zamani 2014^[90]^	55/M	Yes/MI	IHD	PCI	None	TCP	CAG	Smoking
40	Dai 2014^[91]^	66/M	Yes	None	PCI	Yes	TCP	CAG	HP/DM
41	Pourafkari 2014^[92]^	44/M	Yes/MI	IHD	PCI	None	TCP	CAG	None
42	Agustin 2014^[93]^	40/M	None	None	Medicine	None	TCP	CT	None
43	Ha S J 2014^[94]^	63/M	Yes	None	Medicine	None	ATCP	CT/CAG	HP
44	Egashira 2014^[95]^	64/M	None	Valvular heart disease /BAV/AEE/PFO	None	None	Dyspnea	CAG	None
45	Singh 2015^[96]^	60/M	Yes	None	Medicine	None	Atypical chest discomfort	CAG	HP/DM
46	Singh 2015^[96]^	68/F	Yes	None	PCI	None	TCP	CAG	Asthma
47	Buccheri 2015^[97]^	75/F	Yes/MI	None	PCI	Yes	NA	CAG	HP/IGT
48	Inaba 2015^[98]^	48/M	None	None	Medicine	Yes	TCP	CT	DM
49	García-Blas 2015^[99]^	87/M	Yes	None	PCI	None	Syncope	CAG	None
50	Patil 2016^[100]^	55/F	Yes/MI	IHD	PCI	Yes	TCP	CT/CAG	HP/DM
51	Witkowska 2017^[101]^	40/F	Yes/MI	None	PCI	None	NA	CT/CAG	Metabolic syndrome
52	Gatti 2017^[102]^	62/M	None	None	Medicine	None	TCP	CT	HP/IGT
53	Lee H-C 2018^[103]^	43/M	None	None	Medicine	Yes	Dyspnea /chest tightness	CT	None
54	Cirakoglu 2018^[1]^	37/M	None	Atrioventricular block / transposition of the great arteries	Medicine	None	TCP	CT/CAG	HP
55	Iftikhar 2019^[104]^	45/M	Yes/MI	None	PCI	None	TCP	CAG	HP/Smoking
56	Silva Matte 2019^[105]^	80/M	Yes/MI	IHD	PCI	None	TCP	CAG	HP/HT/Smoking
57	Phan 2019^[106]^	56/M	Yes/MI	BAV	PCI	None	TCP	CAG	HP/Smoking
58	Katsaras 2021^[107]^	59/F	None	PFO/ASD	Medicine	None	TCP	CT	HP/DM/asthma/Brugada sydrome
59	Ahmed 2021^[108]^	66/F	Yes	None	Medicine	Yes	ATCP	CT/CAG	HP

ATCP, atypical chest pain; TCP, typical chest pain; PCI, percutaneous coronary intervention; CABG, coronary artery bypass grafting; CT,computed tomography; CAG, Coronary angiography; (O)MI, (old)myocardial infarction; M, male; F, female; AF, atrial fibrillation; RBBB, right bundle branch block; RV, right ventricle; IHD, ischemic heart disease; BAV, bicuspid aortic valve; PFO, patent foramen ovale; AAE, annuloaortic ectasia; ASD, atrial septal defect; IGT, impaired glucose tolerance; HT, hypothyroidism; HP, hypertension; DM, Diabetes Mellitus; NA, not available.

#### 3.4.1. SCA and coronary heart disease

SCA is generally considered to be a benign abnormality; however, some authors have reported that 15% of SCA patients develop myocardial ischaemia as a direct consequence of coronary artery abnormalities (78). SCA abnormalities showed a higher risk of coronary atherosclerosis in a study (113). However, the relationship between congenital abnormalities and atherosclerosis is controversial. Tanriverdi H and Rudan D suggested that atherosclerosis in patients with coronary artery abnormalities was a coincidence (114, 115). According to Shirani's review, 15% of patients with isolated SCA have evidence of myocardial ischaemia without significant atherosclerotic stenosis (116). There were 36 (61%) patients with coronary artery absence combined with coronary atherosclerotic heart disease (CHD); of them, coronary artery bypass grafting was performed in 5 patients, and percutaneous coronary intervention was conducted in 20 patients, which was higher than the general population. SCA anomaly by itself is unlikely to induce myocardial ischaemia, and it has been considered a benign lesion (98). We consider that SCA promotes the occurrence of CHD. When evaluating clinical symptoms and the degree of myocardial area at risk, it is extremely crucial to refer patients with combined SCA and CHD for selective coronary revascularization. Previous studies have shown that interventional surgery for SCA with atherosclerosis has potentially serious consequences; thus, it is rarely performed (92, 102). However, our report revealed that the proportion of patients undergoing interventional surgery in this condition was as high as 69% (25/36). Therefore, interventional operation can be performed successfully in a patient who have SCA with CHD when the anatomy is appropriate.

#### 3.4.2. SCA with congenital heart disease

Congenital heart disease is uncommon in the L-I pattern of SCA, accounting for 7% (4/59). Patent foramen ovale and bicuspid aortic valve were reported in 2 patients, and one of them had annuloaortic ectasia leading to heart failure (101, 105, 107). Congenitally corrected transposition in a situs inversus was shown in a patient (1).

#### 3.4.3. SCA with arrhythmia

Overall, 10% (6/59) of SCA patients have shown arrhythmia, including 3 patients with right bundle branch block (68, 85, 102), 2 patients with atrial fibrillation (30, 43), 1 patient with atrioventricular block (72) and 1 patient with Brugada syndrome (105). The risk of complete atrioventricular block in patients with atrioventricular discordance has been demonstrated, and SCA anomaly likely does not present an additional risk for atrioventricular block.

#### 3.4.4. SCA with cardiomyopathy

The present study showed that 12% (7/59) of patients had coexisting cardiomyopathy, 6 had ischaemic cardiomyopathy due to CHD, and 1 had valvular heart disease. To our knowledge, there is no case of SCA with DCM to date. There were 13 patients with hyperlipidemia.

17 patients were diagnosed with SCA by computed tomography (CT). It has been reported that CTCA is the primary method for determining the diagnosis of SCA and can help delineate the course of the proximal artery. CTCA is also an interesting new model; in addition to being noninvasive, it reveals adjacent structures to understand the origin and development of coronary arteries (89).

Guidelines regarding the management of SCA are difficult to establish, and treatment is guided by symptoms and the presence or severity of atherosclerosis stenosis or the occurrence of acute coronary syndrome. The prognosis of patients ranges from a good normal life expectancy to sudden death.

## 4. Summary

According to the clinical symptoms and examination results of the patient, we believe that the patient's SCA was benign and was not the cause of his DCM. Another case of absence of the RCA with coexistence of the *SCN5A* gene mutation was previously reported by Katsaras D et al. (106). In this case, a patient with familial Brugada syndrome with absence of the RCA tested positive for a *SCN5A* gene C.664C. > T variant and presented with patent foramen ovale. In contrast, our case did not show Brugada syndrome rather than demonstrating DCM. As we discussed above, the *SCN5A* gene variant is associated with a spectrum of clinical diseases, and loss of sodium channel function has a critical role in the development of cardiomyopathy (48). The proband and his children carried the same three mutations. His children refused echocardiography but currently have no symptoms of DCM. The genetic background of patients presenting with DCM is complex; some studies have shown that more than 38% have a compound heterozygous or combined mutation, and 12.8% have three or more mutations (43–45). It is unclear whether the *SCN5A* gene mutation correlated with RCA absence and DCM in the present case. Meanwhile, the proband and his children carried an *APOA5* gene mutation, which is considered a suspected pathogenic variant resulting in hypertriglyceridemia or hyperlipoproteinemia independent of the *SCN5A* mutation.

## 5. Conclusion

This is the first case of SCA combined with DCM of the *SCN5A* C.1858C > T (P.arg620Cys) mutation, which is the cause of DCM and Brugada syndrome. We should carefully identify and reduce life-threatening events in clinical practice to improve the survival rate. As with many other coronary artery abnormalities, coronary angiography is the gold standard method of diagnosis; importantly, CTCA plays a crucial role in diagnosis when considered as a noninvasive operation. Moreover, the underlying aetiological and pathological link between SCA and DCM remains to be explored.
