# Supplementary material for: Single coronary artery presenting dilated cardiomyopathy and hyperlipidemia with the SCN5A and APOA5 gene mutation: A case report and review of the literature
Source: Front Cardiovasc Med. 2023 May 22;10:1113886. doi: 10.3389/fcvm.2023.1113886 (PMC10242075; doi:10.3389/fcvm.2023.1113886)
Supplement: Supplementary file 1 [file Table1.docx]

**SUPPLEMENTARY TABLE 1** NGS data of the patient

| **Sample ID** | | **#Chr** | **Start** | | **Stop** | | **Gene Symbol** | **Transcript** | **cHGVS** | **pHGVS** | **ExIn ID** | **Zygosity** | **Function** | **rsID** | **dbSNP Allele Freq** | **1000G AF** | **ESP6500 AF** | **GnomAD AF** |
| --- | --- | --- | --- | --- | --- | --- | --- | --- | --- | --- | --- | --- | --- | --- | --- | --- | --- | --- |
| 22B02836804 | | chr1 | 1168188 | | 1168188 | | B3GALT6 | NM_080605.3 | c.530G>C | p.Arg177Pro | EX1E | Het | missense |  |  |  |  |  |
| 22B02836804 | | chr1 | 1447702 | | 1447702 | | ATAD3A | NM_018188.3 | c.54G>C | p.(Pro18=) | EX1 | Het | coding-synon |  |  |  |  |  |
| 22B02836804 | | chr1 | 1447873 | | 1447877 | | ATAD3A | NM_018188.3 | c.205+20_205+24delGCGGC |  | IVS1 | Het | splice+20 | rs761661834 | 0.004647 |  |  | 0.009069 |
| 22B02836804 | | chr1 | 2160390 | | 2160391 | | SKI | NM_003036.3 | c.189_194dupGGTGCC | p.Pro65_Ala66insValPro | EX1 | Het | cds-ins |  |  |  |  |  |
| 22B02836804 | | chr1 | 5993313 | | 5993313 | | NPHP4 | NM_015102.3 | c.1196A>G | p.Glu399Gly | EX10 | Het | missense | rs117898549 | 0.000826 | 0.002596 |  | 0.000744 |
| 22B02836804 | | chr1 | 6508836 | | 6508836 | | ESPN | NM_031475.2 | c.1600C>A | p.Arg534Ser | EX8 | Het | missense |  |  |  |  |  |
| 22B02836804 | | chr1 | 11856378 | | 11856378 | | MTHFR | NM_005957.4 | c.665C>T | p.Ala222Val | EX5 | Hom | missense | rs1801133 | 0.301359 | 0.245407 | 0.270567 | 0.314859 |
| 22B02836804 | | chr1 | 19204117 | | 19204117 | | ALDH4A1 | NM_170726.2 | c.941-11G>C |  | IVS9 | Het | splice-20 |  |  |  |  |  |
| 22B02836804 | | chr1 | 27240266 | | 27240275 | | NR0B2 | NM_021969.2 | c.157_166delCATCGCACCT | p.His53Alafs*50 | EX1 | Het | frameshift | rs540387719 | 0.000557 | 0.000799 |  | 0.000523 |
| 22B02836804 | | chr1 | 29323765 | | 29323765 | | EPB41 | NM_203342.2 | c.93T>C | p.(Cys31=) | EX5 | Het | coding-synon | rs756996105 | 0.000115 |  |  | 0.000099 |
| 22B02836804 | | chr1 | 31347439 | | 31347439 | | SDC3 | NM_014654.3 | c.871-4G>T |  | IVS3 | Het | splice-10 | rs188640187 | 0.005523 | 0.001398 | 0.000923 | 0.006507 |
| 22B02836804 | | chr1 | 31349557 | | 31349557 | | SDC3 | NM_014654.3 | c.712G>C | p.Val238Leu | EX3 | Het | missense | rs200515984 | 0.000308 | 0.000399 |  | 0.000235 |
| 22B02836804 | | chr1 | 33478900 | | 33478900 | | AK2 | NM_013411.4 | c.602A>T | p.Tyr201Phe | EX6 | Het | missense | rs113711467 | 0.00972 |  |  | 0.000215 |
| 22B02836804 | | chr1 | 35250901 | | 35250901 | | GJB3 | NM_024009.2 | c.538C>T | p.Arg180* | EX2E | Het | nonsense | rs74315319 | 0.000087 | 0.0002 |  | 0.000088 |
| 22B02836804 | | chr1 | 40557075 | | 40557075 | | PPT1 | NM_000310.3 | c.363-4G>A |  | IVS3 | Het | splice-10 | rs117284255 | 0.009286 | 0.009585 | 0.001076 | 0.010325 |
| 22B02836804 | | chr1 | 41284190 | | 41284190 | | KCNQ4 | NM_004700.3 | c.546C>G | p.Phe182Leu | EX4 | Het | missense | rs80358273 | 0.00034 | 0.000599 |  | 0.000329 |
| 22B02836804 | | chr1 | 47882174 | | 47882174 | | FOXE3 | NM_012186.2 | c.187C>G | p.Arg63Gly | EX1E | Het | missense |  |  |  |  |  |
| 22B02836804 | | chr1 | 47882177 | | 47882177 | | FOXE3 | NM_012186.2 | c.190C>G | p.Arg64Gly | EX1E | Het | missense |  |  |  |  |  |
| 22B02836804 | | chr1 | 47882180 | | 47882180 | | FOXE3 | NM_012186.2 | c.193C>G | p.Arg65Gly | EX1E | Het | missense |  |  |  |  |  |
| 22B02836804 | | chr1 | 61872215 | | 61872216 | | NFIA | NM_001145511.1 | c.1231-19_1231-18insC |  | IVS8 | Het | splice-20 | rs759758399 | 0.073717 |  |  | 0.001845 |
| **Sample ID** | | **#Chr** | **Start** | | **Stop** | | **Gene Symbol** | **Transcript** | **cHGVS** | **pHGVS** | **ExIn ID** | **Zygosity** | **Function** | **rsID** | **dbSNP Allele Freq** | **1000G AF** | **ESP6500 AF** | **GnomAD AF** |
| 22B02836804 | | chr1 | 68904775 | | 68904775 | | RPE65 | NM_000329.2 | c.859-11C>A |  | IVS8 | Het | splice-20 |  |  |  | 0 |  |
| 22B02836804 | | chr1 | 92446180 | | 92446180 | | BRDT | NM_001242806.1 | c.1300-20G>A |  | IVS8 | Het | splice-20 | rs201604833 | 0.000819 | 0.001398 |  | 0.00072 |
| 22B02836804 | | chr1 | 152276761 | | 152276761 | | FLG | NM_002016.1 | c.10601A>G | p.Asn3534Ser | EX3E | Het | missense | rs567405841 | 0.000396 | 0.004593 |  | 0.000214 |
| 22B02836804 | | chr1 | 152276762 | | 152276762 | | FLG | NM_002016.1 | c.10600A>C | p.Asn3534His | EX3E | Het | missense | rs12732870 | 0.000095 | 0.001797 |  | 0.000012 |
| 22B02836804 | | chr1 | 152278636 | | 152278636 | | FLG | NM_002016.1 | c.8726G>T | p.Gly2909Val | EX3E | Het | missense | rs542663583 | 0.000032 | 0.0002 |  | 0.000012 |
| 22B02836804 | | chr1 | 152281635 | | 152281635 | | FLG | NM_002016.1 | c.5727G>T | p.Arg1909Ser | EX3E | Het | missense | rs112627337 | 0.000008 |  | 0 | 0.000008 |
| 22B02836804 | | chr1 | 152284576 | | 152284576 | | FLG | NM_002016.1 | c.2786G>T | p.Gly929Val | EX3E | Het | missense | rs143382793 | 0.002949 |  |  | 0.000246 |
| 22B02836804 | | chr1 | 152284597 | | 152284597 | | FLG | NM_002016.1 | c.2765T>G | p.Ile922Ser | EX3E | Het | missense | rs201928359 | 0.001064 |  |  | 0.000004 |
| 22B02836804 | | chr1 | 152284603 | | 152284604 | | FLG | NM_002016.1 | c.2758_2759insGG | p.Ala920Glyfs*203 | EX3E | Het | frameshift | rs770547637 | 0.00098 |  |  | 0.000004 |
| 22B02836804 | | chr1 | 152284606 | | 152284607 | | FLG | NM_002016.1 | c.2755_2756delCA | p.His919Cysfs*5 | EX3E | Het | frameshift | rs759142251 | 0.000956 |  |  | 0.000004 |
| 22B02836804 | | chr1 | 165182873 | | 165182873 | | LMX1A | NM_177398.3 | c.669+5A>G |  | IVS5 | Het | splice+10 | rs144209134 | 0.000656 | 0.000799 |  | 0.000746 |
| 22B02836804 | | chr1 | 167802253 | | 167802253 | | ADCY10 | NM_018417.4 | c.3565C>A | p.(Arg1189=) | EX25 | Het | coding-synon | rs141732535 | 0.000633 | 0.002596 |  | 0.000525 |
| 22B02836804 | | chr1 | 176671882 | | 176671885 | | PAPPA2 | NM_020318.2 | c.3365+11_3365+14delTGTG |  | IVS9 | Het | splice+20 |  |  |  |  |  |
| 22B02836804 | | chr1 | 183086559 | | 183086559 | | LAMC1 | NM_002293.3 | c.1669C>T | p.Arg557Trp | EX9 | Het | missense | rs150392886 | 0.001963 | 0.005391 | 0.000077 | 0.001808 |
| 22B02836804 | | chr1 | 197059126 | | 197059126 | | ASPM | NM_018136.4 | c.9918T>C | p.(Cys3306=) | EX25 | Het | coding-synon |  |  |  |  |  |
| 22B02836804 | | chr1 | 200572995 | | 200572995 | | KIF14 | NM_014875.2 | c.1835C>T | p.Thr612Met | EX9 | Het | missense | rs747430628 | 0.000074 |  |  | 0.000111 |
| 22B02836804 | | chr1 | 207644254 | | 207644254 | | CR2 | NM_001006658.2 | c.1395A>G | p.(Gln465=) | EX7 | Het | coding-synon | rs188078143 | 0.000635 | 0.001597 | 0.000077 | 0.000626 |
| 22B02836804 | | chr1 | 207718903 | | 207718903 | | CR1 | NM_000651.4 | c.2414-18G>A |  | IVS14 | Hom | splice-20 | rs150543427 | 0.8 |  |  | 0 |
| 22B02836804 | | chr1 | 210573930 | | 210573930 | | HHAT | NM_018194.4 | c.392G>C | p.Arg131Pro | EX5 | Het | missense | rs370012146 | 0.000404 | 0.0002 | 0 | 0.000251 |
| 22B02836804 | | chr1 | 220275746 | | 220275746 | | IARS2 | NM_018060.3 | c.741G>A | p.(Pro247=) | EX5 | Het | coding-synon | rs199601961 | 0.000468 | 0.001198 |  | 0.000395 |
| 22B02836804 | | chr1 | 224581555 | | 224581555 | | WDR26 | NM_025160.6 | c.1935T>C | p.(Pro645=) | EX13 | Het | coding-synon | rs142326612 | 0.000518 | 0.000399 |  | 0.000495 |
| **Sample ID** | | **#Chr** | **Start** | | **Stop** | | **Gene Symbol** | **Transcript** | **cHGVS** | **pHGVS** | **ExIn ID** | **Zygosity** | **Function** | **rsID** | **dbSNP Allele Freq** | **1000G AF** | **ESP6500 AF** | **GnomAD AF** |
| 22B02836804 | | chr1 | 226027067 | | 226027068 | | EPHX1 | NM_001136018.2 | c.722+20_722+21insGTGT |  | IVS5 | Het | splice+20 | rs766429870 | 0 |  |  |  |
| 22B02836804 | | chr1 | 234743616 | | 234743616 | | IRF2BP2 | NM_182972.2 | c.1049-18A>G |  | IVS1 | Het | splice-20 | rs142257661 | 0.000378 | 0.002196 |  | 0.000309 |
| 22B02836804 | | chr1 | 241663903 | | 241663904 | | FH | NM_000143.3 | c.1237-14_1237-13delTC |  | IVS8 | Het | splice-20 |  |  |  |  |  |
| 22B02836804 | | chr10 | 94005 | | 94005 | | TUBB8 | NM_177987.2 | c.327C>T | p.(Gly109=) | EX4E | Het | coding-synon | rs374716232 | 0.002129 |  |  | 0.000277 |
| 22B02836804 | | chr10 | 94006 | | 94006 | | TUBB8 | NM_177987.2 | c.326G>T | p.Gly109Val | EX4E | Het | missense | rs368995010 | 0.001731 |  |  | 0.000008 |
| 22B02836804 | | chr10 | 94011 | | 94011 | | TUBB8 | NM_177987.2 | c.321C>A | p.(Thr107=) | EX4E | Het | coding-synon | rs782307404 | 0.007036 |  |  | 0.001519 |
| 22B02836804 | | chr10 | 13330389 | | 13330389 | | PHYH | NM_006214.3 | c.649C>G | p.Leu217Val | EX6 | Het | missense | rs200627042 | 0.000056 | 0.0002 |  | 0.000032 |
| 22B02836804 | | chr10 | 55626631 | | 55626631 | | PCDH15 | NM_033056.3 | c.3502-14delT |  | IVS26 | Het | splice-20 | rs530072775 | 0.004433 | 0.003594 | 0.007909 | 0.001891 |
| 22B02836804 | | chr10 | 71683573 | | 71683573 | | COL13A1 | NM_001130103.1 | c.1213G>T | p.Asp405Tyr | EX23 | Het | missense | rs117194484 | 0.000786 | 0.002196 | 0.000081 | 0.000608 |
| 22B02836804 | | chr10 | 72643718 | | 72643719 | | PCBD1 | NM_000281.2 | c.303_304delGT | p.Ser102Hisfs*10 | EX4E | Het | frameshift |  |  |  |  |  |
| 22B02836804 | | chr10 | 73466667 | | 73466667 | | CDH23 | NM_022124.5 | c.2967C>T | p.(Asn989=) | EX26 | Het | coding-synon | rs745617102 | 0.000038 |  |  | 0.000018 |
| 22B02836804 | | chr10 | 102789821 | | 102789821 | | PDZD7 | NM_001195263.1 | c.156C>T | p.(Asn52=) | EX2 | Het | coding-synon | rs150917752 | 0.001524 | 0.001997 | 0.000231 | 0.001324 |
| 22B02836804 | | chr10 | 104835985 | | 104835985 | | CNNM2 | NM_017649.4 | c.2376C>T | p.(Pro792=) | EX7 | Het | coding-synon | rs573050677 | 0.000228 | 0.000599 |  | 0.000122 |
| 22B02836804 | | chr10 | 114925413 | | 114925413 | | TCF7L2 | NM_030756.4 | c.1473G>C | p.(Pro491=) | EX14E | Het | coding-synon |  |  |  |  | 0.00008 |
| 22B02836804 | | chr10 | 114925414 | | 114925414 | | TCF7L2 | NM_030756.4 | c.1474A>C | p.Asn492His | EX14E | Het | missense |  |  |  |  | 0 |
| 22B02836804 | | chr10 | 118306934 | | 118306934 | | PNLIP | NM_000936.2 | c.175A>G | p.Thr59Ala | EX3 | Het | missense | rs147154871 | 0.000406 | 0.001797 |  | 0.000375 |
| 22B02836804 | | chr10 | 124810705 | | 124810705 | | ACADSB | NM_001609.3 | c.1128+3delA |  | IVS9 | Het | splice+10 |  |  |  |  |  |
| 22B02836804 | | chr10 | 131665499 | | 131665499 | | EBF3 | NM_001005463.2 | c.918C>T | p.(Thr306=) | EX10 | Het | coding-synon | rs76892753 | 0.000144 | 0.0002 |  | 0.000143 |
| 22B02836804 | | chr10 | 135103311 | | 135103312 | | TUBGCP2 | NM_001256617.1 | c.1444+16_1444+17insAGGGACTTGGCGTCCGCGGCGCAGCGGGC |  | IVS10 | Hom | splice+20 |  |  |  |  |  |
| 22B02836804 | | chr11 | 613363 | | 613363 | | IRF7 | NM_001572.3 | c.1080G>A | p.(Glu360=) | EX9 | Het | coding-synon | rs766831508 | 0.000018 |  |  | 0.000025 |
| **Sample ID** | | **#Chr** | **Start** | | **Stop** | | **Gene Symbol** | **Transcript** | **cHGVS** | **pHGVS** | **ExIn ID** | **Zygosity** | **Function** | **rsID** | **dbSNP Allele Freq** | **1000G AF** | **ESP6500 AF** | **GnomAD AF** |
| 22B02836804 | | chr11 | 639650 | | 639651 | | DRD4 | NM_000797.3 | c.417_418insGCCGTG | p.Val139_Pro140insAlaVal | EX3 | Het | cds-ins | rs751106635 | 0.998977 |  |  | 0.000952 |
| 22B02836804 | | chr11 | 1260145 | | 1260145 | | MUC5B | NM_002458.2 | c.3342T>C | p.(Cys1114=) | EX26 | Het | coding-synon | rs75799984 | 0.5 |  |  |  |
| 22B02836804 | | chr11 | 1260157 | | 1260157 | | MUC5B | NM_002458.2 | c.3354G>C | p.(Ser1118=) | EX26 | Het | coding-synon |  |  |  |  |  |
| 22B02836804 | | chr11 | 1260160 | | 1260160 | | MUC5B | NM_002458.2 | c.3357T>G | p.(Gly1119=) | EX26 | Het | coding-synon |  |  |  |  |  |
| 22B02836804 | | chr11 | 1260163 | | 1260163 | | MUC5B | NM_002458.2 | c.3360C>T | p.(Gly1120=) | EX26 | Het | coding-synon | rs79220674 | 0.000266 | 0.000399 |  | 0.000053 |
| 22B02836804 | | chr11 | 1260175 | | 1260175 | | MUC5B | NM_002458.2 | c.3372T>C | p.(Cys1124=) | EX26 | Het | coding-synon | rs75760167 | 1 |  |  |  |
| 22B02836804 | | chr11 | 1260193 | | 1260193 | | MUC5B | NM_002458.2 | c.3390T>C | p.(Ala1130=) | EX26 | Het | coding-synon |  |  |  |  |  |
| 22B02836804 | | chr11 | 1260214 | | 1260214 | | MUC5B | NM_002458.2 | c.3411C>T | p.(His1137=) | EX26 | Het | coding-synon | rs79585387 | 0.5 |  |  |  |
| 22B02836804 | | chr11 | 1260217 | | 1260217 | | MUC5B | NM_002458.2 | c.3414C>A | p.Asp1138Glu | EX26 | Het | missense |  |  |  |  | 0 |
| 22B02836804 | | chr11 | 1260219 | | 1260219 | | MUC5B | NM_002458.2 | c.3416C>T | p.Ala1139Val | EX26 | Het | missense | rs75935363 | 0.000041 |  |  | 0.000056 |
| 22B02836804 | | chr11 | 1260220 | | 1260220 | | MUC5B | NM_002458.2 | c.3417G>A | p.(Ala1139=) | EX26 | Het | coding-synon | rs61733358 | 0.000286 |  | 0 | 0.000126 |
| 22B02836804 | | chr11 | 1260244 | | 1260244 | | MUC5B | NM_002458.2 | c.3441T>C | p.(Thr1147=) | EX26 | Het | coding-synon | rs76590120 | 0.5 |  |  |  |
| 22B02836804 | | chr11 | 1260248 | | 1260248 | | MUC5B | NM_002458.2 | c.3445G>A | p.Asp1149Asn | EX26 | Het | missense |  |  |  |  |  |
| 22B02836804 | | chr11 | 1260249 | | 1260249 | | MUC5B | NM_002458.2 | c.3446A>G | p.Asp1149Gly | EX26 | Het | missense | rs77875446 | 0.5 |  |  |  |
| 22B02836804 | | chr11 | 1260252 | | 1260252 | | MUC5B | NM_002458.2 | c.3449C>T | p.Thr1150Ile | EX26 | Het | missense | rs78681629 | 0.5 |  |  |  |
| 22B02836804 | | chr11 | 1275978 | | 1275978 | | MUC5B | NM_002458.2 | c.15532G>C | p.Val5178Leu | EX35 | Het | missense | rs371813527 | 0.000097 | 0.000399 | 0.000078 | 0.000065 |
| 22B02836804 | | chr11 | 20622736 | | 20622736 | | SLC6A5 | NM_004211.3 | c.65C>G | p.Ala22Gly | EX2 | Het | missense |  |  |  |  |  |
| 22B02836804 | | chr11 | 20673907 | | 20673907 | | SLC6A5 | NM_004211.3 | c.2143A>G | p.Met715Val | EX15 | Het | missense | rs140461634 | 0.000008 |  | 0.000077 | 0.000004 |
| 22B02836804 | | chr11 | 47296401 | | 47296401 | | MADD | NM_003682.3 | c.350G>A | p.Arg117His | EX3 | Het | missense | rs77637447 | 0.000143 | 0.000599 |  | 0.000167 |
| 22B02836804 | | chr11 | 61725599 | | 61725600 | | BEST1 | NM_004183.3 | c.715-19_715-18insTCCTCCTCC |  | IVS6 | Hom | splice-20 | rs113492158 | 0.5 |  |  |  |
| 22B02836804 | | chr11 | 67763203 | | 67763203 | | UNC93B1 | NM_030930.2 | c.1242G>C | p.(Leu414=) | EX9 | Het | coding-synon |  |  |  |  | 0.000026 |
| **Sample ID** | | **#Chr** | **Start** | | **Stop** | | **Gene Symbol** | **Transcript** | **cHGVS** | **pHGVS** | **ExIn ID** | **Zygosity** | **Function** | **rsID** | **dbSNP Allele Freq** | **1000G AF** | **ESP6500 AF** | **GnomAD AF** |
| 22B02836804 | | chr11 | 67763229 | | 67763229 | | UNC93B1 | NM_030930.2 | c.1216G>A | p.Val406Met | EX9 | Het | missense | rs778453022 | 0 |  |  | 0.000007 |
| 22B02836804 | | chr11 | 67763230 | | 67763230 | | UNC93B1 | NM_030930.2 | c.1215G>C | p.(Leu405=) | EX9 | Het | coding-synon | rs1055846 | 0 |  |  | 0.000007 |
| 22B02836804 | | chr11 | 67811694 | | 67811694 | | TCIRG1 | NM_006019.3 | c.903C>T | p.(Ala301=) | EX9 | Het | coding-synon | rs773632286 | 0.000229 |  |  | 0.000063 |
| 22B02836804 | | chr11 | 68207394 | | 68207394 | | LRP5 | NM_002335.2 | c.4488+10G>A |  | IVS21 | Het | splice+10 | rs202206612 | 0.000849 | 0.000998 |  | 0.000402 |
| 22B02836804 | | chr11 | 68566672 | | 68566672 | | CPT1A | NM_001031847.2 | c.693+14C>T |  | IVS6 | Het | splice+20 | rs202064999 | 0.000016 | 0.0002 |  | 0.000024 |
| 22B02836804 | | chr11 | 68566797 | | 68566797 | | CPT1A | NM_001031847.2 | c.582G>T | p.Met194Ile | EX6 | Het | missense |  |  |  |  |  |
| 22B02836804 | | chr11 | 70858365 | | 70858365 | | SHANK2 | NM_012309.3 | c.8G>A | p.Arg3His | EX2 | Het | missense | rs369450251 | 0.00043 | 0.001398 | 0.000219 | 0.000278 |
| 22B02836804 | | chr11 | 78516320 | | 78516321 | | TENM4 | NM_001098816.2 | c.2179+16_2179+17insGAAGGGTGGGTGGAGGAGGAGTGGGGGTGGGGAAGAATGAGGGGTGGGGTGTGGAGGGTGGGGAG |  | IVS15 | Hom | splice+20 |  |  |  |  |  |
| 22B02836804 | | chr11 | 84865604 | | 84865606 | | DLG2 | NM_001142699.1 | c.276_278delGAC | p.Thr94del | EX5 | Het | cds-del |  |  |  |  | 0.000013 |
| 22B02836804 | | chr11 | 88045646 | | 88045646 | | CTSC | NM_001814.4 | c.395G>A | p.Arg132Gln | EX3 | Het | missense | rs575727793 | 0.000134 | 0.0002 |  | 0.000131 |
| 22B02836804 | | chr11 | 108181031 | | 108181031 | | ATM | NM_000051.3 | c.5907T>G | p.Asp1969Glu | EX39 | Het | missense |  |  |  |  |  |
| 22B02836804 | | chr11 | 112099026 | | 112099026 | | PTS | NM_000317.2 | c.84-291A>G |  | IVS1 | Het | intron |  |  |  |  |  |
| 22B02836804 | | chr11 | 116660952 | | 116660955 | | APOA5 | NM_052968.4 | c.990_993delAACA | p.Asp332Valfs*5 | EX4E | Het | frameshift | rs774150500 | 0.000008 |  |  | 0.000008 |
| 22B02836804 | | chr11 | 116660952 | | 116660955 | | ZNF259 | NM_003904.3 | c.-2249_-2246delAACA |  |  | Het | promoter | rs774150500 | 0.000008 |  |  | 0.000008 |
| 22B02836804 | | chr11 | 122954390 | | 122954390 | | CLMP | NM_024769.2 | c.554T>C | p.Ile185Thr | EX4 | Het | missense | rs537923955 | 0.000032 | 0.0002 |  | 0.000028 |
| 22B02836804 | | chr11 | 124745919 | | 124745919 | | ROBO3 | NM_022370.3 | c.2491A>G | p.Met831Val | EX16 | Het | missense |  |  |  |  |  |
| 22B02836804 | | chr12 | 6167131 | | 6167131 | | VWF | NM_000552.3 | c.1613C>T | p.Pro538Leu | EX14 | Het | missense | rs139196998 | 0.000386 | 0.000599 | 0.000077 | 0.000395 |
| 22B02836804 | | chr12 | 6701717 | | 6701717 | | CHD4 | NM_001273.2 | c.2790T>C | p.(Phe930=) | EX19 | Het | coding-synon |  |  |  |  | 0.000004 |
| 22B02836804 | | chr12 | 7045891 | | 7045907 | | ATN1 | NM_001007026.1 | c.1508_1509insGCAGCA | p.Gln502_His503insGlnGln | EX5 | Het | cds-ins |  |  |  |  |  |
| **Sample ID** | | **#Chr** | **Start** | | **Stop** | | **Gene Symbol** | **Transcript** | **cHGVS** | **pHGVS** | **ExIn ID** | **Zygosity** | **Function** | **rsID** | **dbSNP Allele Freq** | **1000G AF** | **ESP6500 AF** | **GnomAD AF** |
| 22B02836804 | | chr12 | 7045892 | | 7045906 | | ATN1 | NM_001007026.1 | c.1494_1508delGCAGCAGCAGCAGCA | p.Gln498_Gln502del | EX5 | Het | cds-del | rs377147612 | 0 |  |  |  |
| 22B02836804 | | chr12 | 14829843 | | 14829843 | | GUCY2C | NM_004963.3 | c.893C>T | p.Thr298Met | EX7 | Het | missense | rs148946391 | 0.001151 | 0.001398 | 0.000692 | 0.001047 |
| 22B02836804 | | chr12 | 14994015 | | 14994015 | | ART4 | NM_021071.2 | c.217G>A | p.Val73Ile | EX2 | Het | missense | rs182981694 | 0.000737 | 0.000998 | 0.000077 | 0.000709 |
| 22B02836804 | | chr12 | 31255366 | | 31255367 | | DDX11 | NM_030653.3 | c.2285_2296dupAGAGAGGCCAGG | p.Gln765_Val766insGluArgGlyGln | EX23 | Het | cds-ins |  |  |  |  |  |
| 22B02836804 | | chr12 | 40677699 | | 40677699 | | LRRK2 | NM_198578.3 | c.2264C>T | p.Pro755Leu | EX19 | Het | missense | rs34410987 | 0.000729 | 0.001597 |  | 0.000741 |
| 22B02836804 | | chr12 | 46245593 | | 46245593 | | ARID2 | NM_152641.2 | c.3687A>G | p.(Ser1229=) | EX15 | Het | coding-synon | rs76884235 | 0.000048 | 0.000399 |  | 0.00002 |
| 22B02836804 | | chr12 | 52710240 | | 52710240 | | KRT83 | NM_002282.3 | c.1041+12C>T |  | IVS6 | Het | splice+20 | rs201756344 | 0.000222 | 0.000399 | 0.000077 | 0.000259 |
| 22B02836804 | | chr12 | 52908879 | | 52908879 | | KRT5 | NM_000424.3 | c.1620C>T | p.(Val540=) | EX9E | Het | coding-synon | rs202197926 | 0.000308 | 0.000399 |  | 0.000375 |
| 22B02836804 | | chr12 | 56137184 | | 56137185 | | GDF11 | NM_005811.3 | c.116_117insGGC | p.Ala41_Gly42insAla | EX1 | Het | cds-ins | rs759951553 | 0 |  |  | 0 |
| 22B02836804 | | chr12 | 56743250 | | 56743250 | | STAT2 | NM_005419.3 | c.1301C>T | p.Thr434Met | EX15 | Het | missense | rs146115536 | 0.000049 |  | 0.000154 | 0.00008 |
| 22B02836804 | | chr12 | 109924386 | | 109924386 | | UBE3B | NM_130466.3 | c.447+6delA |  | IVS6 | Het | splice+10 |  |  |  |  |  |
| 22B02836804 | | chr13 | 25467010 | | 25467011 | | CENPJ | NM_018451.4 | c.2992-6_2992-5insTT |  | IVS9 | Het | splice-10 | rs746136069 | 0.782814 |  |  | 0.001601 |
| 22B02836804 | | chr13 | 31835236 | | 31835236 | | B3GLCT | NM_194318.3 | c.596+17A>T |  | IVS7 | Het | splice+20 | rs751928458 | 0.000008 |  |  | 0.001905 |
| 22B02836804 | | chr13 | 33590493 | | 33590597 | | KL | NM_004795.3 | c.-86_19del |  | .-EX1 | Het | span |  |  |  |  |  |
| 22B02836804 | | chr13 | 35733806 | | 35733806 | | NBEA | NM_015678.4 | c.3498C>T | p.(His1166=) | EX22 | Het | coding-synon | rs146937597 | 0.000095 | 0.000599 |  | 0.000049 |
| 22B02836804 | | chr13 | 52511626 | | 52511626 | | ATP7B | NM_000053.3 | c.3889G>A | p.Val1297Ile | EX18 | Het | missense | rs148399850 | 0.002298 | 0.003395 | 0.000471 | 0.002397 |
| 22B02836804 | | chr13 | 78492599 | | 78492599 | | EDNRB | NM_000115.3 | c.110C>T | p.Thr37Ile | EX2 | Het | missense |  |  |  |  | 0.000005 |
| 22B02836804 | | chr14 | 35476472 | | 35476472 | | SRP54 | NM_003136.3 | c.256-17T>A |  | IVS4 | Het | splice-20 | rs770515104 | 0.000025 |  |  | 0.000017 |
| 22B02836804 | | chr14 | 35497285 | | 35497286 | | SRP54 | NM_003136.3 | c.1328-9_1328-8insTC |  | IVS14 | Het | splice-10 | rs761221697 | 0.000602 |  |  | 0.000465 |
| 22B02836804 | | chr14 | 39560784 | | 39560784 | | SEC23A | NM_006364.2 | c.500T>C | p.Ile167Thr | EX5 | Het | missense | rs144765020 | 0.000704 | 0.0002 | 0.000308 | 0.000947 |
| **Sample ID** | | **#Chr** | **Start** | | **Stop** | | **Gene Symbol** | **Transcript** | **cHGVS** | **pHGVS** | **ExIn ID** | **Zygosity** | **Function** | **rsID** | **dbSNP Allele Freq** | **1000G AF** | **ESP6500 AF** | **GnomAD AF** |
| 22B02836804 | | chr14 | 52495481 | | 52495481 | | NID2 | NM_007361.3 | c.2489G>A | p.Arg830Gln | EX11 | Het | missense | rs7144523 | 0.008759 | 0.017772 | 0.011226 | 0.008731 |
| 22B02836804 | | chr14 | 64467412 | | 64467412 | | SYNE2 | NM_182914.2 | c.3613G>A | p.Glu1205Lys | EX28 | Het | missense | rs375217349 | 0.000088 | 0.0002 | 0.000085 | 0.000064 |
| 22B02836804 | | chr14 | 66082743 | | 66082743 | | FUT8 | NM_178155.2 | c.251G>A | p.Arg84His | EX4 | Het | missense | rs757041953 | 0.000042 |  |  | 0.000032 |
| 22B02836804 | | chr14 | 70420190 | | 70420190 | | SMOC1 | NM_022137.5 | c.319A>C | p.Lys107Gln | EX3 | Het | missense | rs149628528 | 0.000269 | 0.000399 |  | 0.000263 |
| 22B02836804 | | chr14 | 89034383 | | 89034383 | | ZC3H14 | NM_024824.4 | c.80A>G | p.Asp27Gly | EX3 | Het | missense |  |  |  |  |  |
| 22B02836804 | | chr14 | 99641650 | | 99641650 | | BCL11B | NM_138576.2 | c.1523A>G | p.Glu508Gly | EX4E | Het | missense |  |  |  |  |  |
| 22B02836804 | | chr15 | 28228553 | | 28228553 | | OCA2 | NM_000275.2 | c.1441G>A | p.Ala481Thr | EX14 | Het | missense | rs74653330 | 0.00776 | 0.007987 | 0.001307 | 0.008382 |
| 22B02836804 | | chr15 | 37329154 | | 37329154 | | MEIS2 | NM_002399.3 | c.722G>T | p.Gly241Val | EX8 | Het | missense | rs777260011 | 0.000033 |  |  | 0.00005 |
| 22B02836804 | | chr15 | 43045166 | | 43045166 | | TTBK2 | NM_173500.3 | c.2278C>T | p.Pro760Ser | EX14 | Het | missense | rs117382379 | 0.000095 | 0.000399 |  | 0.000112 |
| 22B02836804 | | chr15 | 43903199 | | 43903200 | | STRC | NM_153700.2 | c.3307-18_3307-17delCA |  | IVS13 | Het | splice-20 | rs796838028 | 0 |  |  |  |
| 22B02836804 | | chr15 | 44858159 | | 44858159 | | SPG11 | NM_025137.3 | c.6892A>G | p.Ile2298Val | EX38 | Het | missense | rs147962000 | 0.001086 | 0.002196 |  | 0.001003 |
| 22B02836804 | | chr15 | 48786463 | | 48786463 | | FBN1 | NM_000138.4 | c.2678-12T>C |  | IVS22 | Het | splice-20 | rs200368037 | 0.00057 | 0.000399 |  | 0.000727 |
| 22B02836804 | | chr15 | 65490706 | | 65490706 | | CILP | NM_003613.3 | c.1918A>G | p.Thr640Ala | EX9E | Het | missense |  |  |  |  |  |
| 22B02836804 | | chr15 | 72260385 | | 72260385 | | MYO9A | NM_006901.3 | c.1926C>T | p.(Ala642=) | EX13 | Het | coding-synon | rs2306487 | 0.000317 | 0.000998 | 0.000154 | 0.000279 |
| 22B02836804 | | chr15 | 74473160 | | 74473160 | | STRA6 | NM_001142617.1 | c.1803C>T | p.(Ala601=) | EX18 | Het | coding-synon | rs2277607 | 0.000293 | 0.0002 |  | 0.000303 |
| 22B02836804 | | chr15 | 77329671 | | 77329673 | | PSTPIP1 | NM_003978.3 | c.*155_*169delAAAAAAAAAAAAAAA |  | EX15E-. | Het | span | rs147238110 | 0.871805 |  |  |  |
| 22B02836804 | | chr16 | 1204054 | | 1204054 | | CACNA1H | NM_021098.2 | c.299+18C>G |  | IVS2 | Het | splice+20 |  |  |  |  |  |
| 22B02836804 | | chr16 | 1547104 | | 1547104 | | TELO2 | NM_016111.3 | c.681G>C | p.Gln227His | EX4 | Het | missense | rs139365746 | 0.00042 | 0.000399 | 0 | 0.000367 |
| 22B02836804 | | chr16 | 2153484 | | 2153484 | | PKD1 | NM_001009944.2 | c.8574C>G | p.(Gly2858=) | EX23 | Het | coding-synon | rs545527733 | 0.000153 | 0.000599 |  | 0.00011 |
| 22B02836804 | | chr16 | 9002233 | | 9002233 | | USP7 | NM_003470.2 | c.1236C>T | p.(Asp412=) | EX12 | Het | coding-synon | rs759732382 | 0.000008 |  |  | 0.000004 |
| 22B02836804 | | chr16 | 11375054 | | 11375054 | | PRM1 | NM_002761.2 | c.42A>G | p.(Arg14=) | EX1 | Het | coding-synon | rs187174862 | 0.00034 | 0.001198 |  | 0.000239 |
| **Sample ID** | | **#Chr** | **Start** | | **Stop** | | **Gene Symbol** | **Transcript** | **cHGVS** | **pHGVS** | **ExIn ID** | **Zygosity** | **Function** | **rsID** | **dbSNP Allele Freq** | **1000G AF** | **ESP6500 AF** | **GnomAD AF** |
| 22B02836804 | | chr16 | 21214437 | | 21214437 | | ZP2 | NM_003460.1 | c.1099+9C>A |  | IVS10 | Het | splice+10 | rs187716957 | 0.000462 | 0.001198 |  | 0.000328 |
| 22B02836804 | | chr16 | 21747639 | | 21747639 | | OTOA | NM_144672.3 | c.2359G>T | p.Glu787* | EX21 | Het | nonsense | rs200988634 | 0.00122 |  |  | 0.000308 |
| 22B02836804 | | chr16 | 23197787 | | 23197787 | | SCNN1G | NM_001039.3 | c.195C>T | p.(Ala65=) | EX2 | Het | coding-synon |  |  |  |  |  |
| 22B02836804 | | chr16 | 23632742 | | 23632742 | | PALB2 | NM_024675.3 | c.3054G>C | p.Glu1018Asp | EX10 | Het | missense | rs183489969 | 0.000324 | 0.000799 |  | 0.000382 |
| 22B02836804 | | chr16 | 31120690 | | 31120690 | | BCKDK | NM_005881.2 | c.146C>A | p.Thr49Asn | EX2 | Het | missense |  |  |  |  |  |
| 22B02836804 | | chr16 | 48258198 | | 48258198 | | ABCC11 | NM_032583.3 | c.538G>A | p.Gly180Arg | EX5 | Hom | missense | rs17822931 | 0.228117 | 0.300919 | 0.098 | 0.223953 |
| 22B02836804 | | chr16 | 55513411 | | 55513411 | | MMP2 | NM_004530.4 | c.20G>A | p.Arg7Gln | EX1 | Het | missense | rs746268212 | 0.000074 |  |  | 0.000025 |
| 22B02836804 | | chr16 | 67867761 | | 67867761 | | CENPT | NM_025082.3 | c.2T>C | p.0? | EX4 | Het | init-loss |  |  |  |  | 0.000012 |
| 22B02836804 | | chr16 | 85952421 | | 85952421 | | IRF8 | NM_002163.2 | c.988+12C>T |  | IVS7 | Het | splice+20 | rs577957168 | 0.000089 | 0.0002 |  | 0.000111 |
| 22B02836804 | | chr16 | 85953846 | | 85953849 | | IRF8 | NM_002163.2 | c.1104+16_1104+19delTTTT |  | IVS8 | Het | splice+20 | rs749732730 | 0.000912 |  |  |  |
| 22B02836804 | | chr16 | 89986025 | | 89986025 | | TUBB3 | NM_001197181.1 | c.-2788T>C |  |  | Het | promoter | rs33932559 | 0.003499 | 0.008387 | 0.000154 | 0.003137 |
| 22B02836804 | | chr16 | 89986025 | | 89986025 | | MC1R | NM_002386.3 | c.359T>C | p.Ile120Thr | EX1E | Het | missense | rs33932559 | 0.003499 | 0.008387 | 0.000154 | 0.003137 |
| 22B02836804 | | chr17 | 5463148 | | 5463148 | | NLRP1 | NM_001033053.2 | c.868A>G | p.Arg290Gly | EX4 | Het | missense | rs201548869 | 0.000095 | 0.0002 |  | 0.000091 |
| 22B02836804 | | chr17 | 8790536 | | 8790536 | | PIK3R5 | NM_001142633.2 | c.1782G>A | p.(Glu594=) | EX12 | Het | coding-synon | rs200935575 | 0.000414 | 0.000998 |  | 0.000471 |
| 22B02836804 | | chr17 | 16842773 | | 16842775 | | TBC1D27 | XM_003846282.1 | c.-6957_-6955delTGA |  |  | Hom | promoter | rs150068036 | 0.5 |  |  |  |
| 22B02836804 | | chr17 | 16842773 | | 16842775 | | TNFRSF13B | NM_012452.2 | c.*86_*88delTGA |  | EX5E | Hom | utr-3 | rs150068036 | 0.5 |  |  |  |
| 22B02836804 | | chr17 | 18034132 | | 18034133 | | MYO15A | NM_016239.3 | c.4038+8_4038+9delGT |  | IVS8 | Het | splice+10 | rs529930336 | 0.234146 |  |  |  |
| 22B02836804 | | chr17 | 18077164 | | 18077164 | | MYO15A | NM_016239.3 | c.10420A>G | p.Ser3474Gly | EX65 | Het | missense | rs150181830 | 0.000534 | 0.000998 |  | 0.000508 |
| 22B02836804 | | chr17 | 19568258 | | 19568258 | | ALDH3A2 | NM_000382.2 | c.1108-3C>T |  | IVS7 | Het | splice-10 | rs148944691 | 0.000555 | 0.000799 |  | 0.000621 |
| 22B02836804 | | chr17 | 26727703 | | 26727703 | | SLC46A1 | NM_080669.4 | c.1245A>T | p.(Pro415=) | EX4 | Het | coding-synon | rs782287467 | 0.00001 |  |  | 0.000004 |
| 22B02836804 | | chr17 | 29677247 | | 29677247 | | NF1 | NM_001042492.2 | c.7368A>G | p.(Lys2456=) | EX50 | Het | coding-synon | rs201287021 | 0.000411 | 0.000399 |  | 0.000497 |
| **Sample ID** | | **#Chr** | **Start** | | **Stop** | | **Gene Symbol** | **Transcript** | **cHGVS** | **pHGVS** | **ExIn ID** | **Zygosity** | **Function** | **rsID** | **dbSNP Allele Freq** | **1000G AF** | **ESP6500 AF** | **GnomAD AF** |
| 22B02836804 | | chr17 | 29677247 | | 29677247 | | NF1 | NM_000267.3 | c.7305A>G | p.(Lys2435=) | EX49 | Het | coding-synon | rs201287021 | 0.000411 | 0.000399 |  | 0.000497 |
| 22B02836804 | | chr17 | 38487505 | | 38487505 | | RARA | NM_000964.3 | c.35G>A | p.Gly12Glu | EX2 | Het | missense |  |  |  | 0 |  |
| 22B02836804 | | chr17 | 41251778 | | 41251778 | | BRCA1 | NM_007294.3 | c.547+14delG |  | IVS7 | Het | splice+20 | rs273902771 | 0.000124 |  |  | 0.000088 |
| 22B02836804 | | chr17 | 42284747 | | 42284747 | | UBTF | NM_001076683.1 | c.2059-12C>T |  | IVS19 | Het | splice-20 |  |  |  |  | 0.000004 |
| 22B02836804 | | chr17 | 42340018 | | 42340018 | | SLC4A1 | NM_000342.3 | c.92T>C | p.Met31Thr | EX3 | Het | missense | rs55773290 | 0.000357 | 0.001398 |  | 0.000246 |
| 22B02836804 | | chr17 | 61497906 | | 61497906 | | TANC2 | NM_025185.3 | c.4563G>A | p.(Val1521=) | EX25E | Het | coding-synon | rs201592811 | 0.000016 | 0.0002 |  | 0 |
| 22B02836804 | | chr17 | 61972500 | | 61972500 | | CSH1 | NM_001317.5 | c.536A>G | p.His179Arg | EX5E | Het | missense | rs570114664 | 0.00004 | 0.000399 |  | 0.00002 |
| 22B02836804 | | chr17 | 71196850 | | 71196850 | | COG1 | NM_018714.2 | c.1216C>G | p.Leu406Val | EX6 | Het | missense |  |  |  |  |  |
| 22B02836804 | | chr17 | 73975026 | | 73975026 | | ACOX1 | NM_004035.6 | c.109+20C>G |  | IVS1 | Het | splice+20 | rs750088491 | 0.000017 |  |  | 0.00002 |
| 22B02836804 | | chr17 | 76457692 | | 76457692 | | DNAH17 | NM_173628.3 | c.9273C>T | p.(Ala3091=) | EX58 | Het | coding-synon | rs548700594 | 0.000358 | 0.000599 |  | 0.000216 |
| 22B02836804 | | chr17 | 78298891 | | 78298891 | | RNF213 | NM_001256071.1 | c.3086T>C | p.Leu1029Ser | EX18 | Het | missense | rs753208141 | 0.000114 |  |  | 0.000049 |
| 22B02836804 | | chr17 | 80863817 | | 80863817 | | TBCD | NM_005993.4 | c.1810C>T | p.Pro604Ser | EX20 | Het | missense | rs575721383 | 0.00073 | 0.000998 |  | 0.000507 |
| 22B02836804 | | chr18 | 10677880 | | 10677881 | | PIEZO2 | NM_022068.2 | c.7614-9_7614-8insT |  | IVS48 | Het | splice-10 | rs771100722 | 0.99063 | 0.004593 |  | 0.007677 |
| 22B02836804 | | chr18 | 21124907 | | 21124908 | | NPC1 | NM_000271.4 | c.1947+16_1947+17insGGGGGG |  | IVS12 | Het | splice+20 |  |  |  |  |  |
| 22B02836804 | | chr18 | 34297913 | | 34297913 | | FHOD3 | NM_025135.2 | c.2127T>C | p.(Asp709=) | EX16 | Het | coding-synon | rs3809993 | 0.000116 |  | 0.000077 | 0.000103 |
| 22B02836804 | | chr18 | 55247336 | | 55247336 | | FECH | NM_001012515.2 | c.163G>T | p.Gly55Cys | EX2 | Het | missense | rs3848519 | 0.02237 | 0.026957 | 0.018299 | 0.022062 |
| 22B02836804 | | chr18 | 77475368 | | 77475368 | | CTDP1 | NM_004715.4 | c.1908C>T | p.(Asp636=) | EX8 | Het | coding-synon | rs143177926 | 0.002675 | 0.007188 | 0.00592 | 0.001274 |
| 22B02836804 | | chr19 | 1218407 | | 1218407 | | STK11 | NM_000455.4 | c.291-9C>G |  | IVS1 | Het | splice-10 |  |  |  |  |  |
| 22B02836804 | | chr19 | 4365494 | | 4365494 | | SH3GL1 | NM_003025.3 | c.316G>A | p.Gly106Ser | EX4 | Het | missense | rs539292999 | 0.000127 | 0.0002 |  | 0.000155 |
| 22B02836804 | | chr19 | 5131352 | | 5131352 | | KDM4B | NM_015015.2 | c.1581C>T | p.(Pro527=) | EX12 | Het | coding-synon | rs779398920 | 0.000017 |  |  | 0.000008 |
| 22B02836804 | | chr19 | 6707259 | | 6707259 | | C3 | NM_000064.2 | c.2073C>G | p.(Arg691=) | EX17 | Het | coding-synon |  |  |  |  |  |
| **Sample ID** | | **#Chr** | **Start** | | **Stop** | | **Gene Symbol** | **Transcript** | **cHGVS** | **pHGVS** | **ExIn ID** | **Zygosity** | **Function** | **rsID** | **dbSNP Allele Freq** | **1000G AF** | **ESP6500 AF** | **GnomAD AF** |
| 22B02836804 | | chr19 | 8609201 | | 8609201 | | MYO1F | NM_012335.3 | c.1504A>G | p.Ile502Val | EX14 | Het | missense | rs200797032 | 0.000509 | 0.000799 |  | 0.000467 |
| 22B02836804 | | chr19 | 12775790 | | 12775790 | | MAN2B1 | NM_000528.3 | c.446A>C | p.Glu149Ala | EX4 | Het | missense |  |  |  |  |  |
| 22B02836804 | | chr19 | 13211778 | | 13211778 | | LYL1 | NM_005583.4 | c.208C>G | p.Pro70Ala | EX2 | Het | missense | rs552041622 | 0.000428 | 0.000399 |  | 0.000138 |
| 22B02836804 | | chr19 | 18271265 | | 18271266 | | PIK3R2 | NM_005027.2 | c.323-16_323-15delCT |  | IVS2 | Het | splice-20 | rs374033727 | 0.00025 | 0.000599 | 0.001757 | 0.000269 |
| 22B02836804 | | chr19 | 18705138 | | 18705138 | | CRLF1 | NM_004750.4 | c.1131C>A | p.His377Gln | EX7 | Het | missense |  |  |  |  |  |
| 22B02836804 | | chr19 | 19309985 | | 19309985 | | RFXANK | NM_003721.2 | c.654C>T | p.(Thr218=) | EX9 | Het | coding-synon | rs8862 | 0.000183 | 0.000599 | 0.000077 | 0.000167 |
| 22B02836804 | | chr19 | 33321587 | | 33321587 | | SLC7A9 | NM_014270.4 | c.1403C>T | p.Pro468Leu | EX13E | Het | missense | rs80283711 | 0.002794 | 0.004193 | 0.000154 | 0.003169 |
| 22B02836804 | | chr19 | 38991613 | | 38991613 | | RYR1 | NM_000540.2 | c.7597G>C | p.Ala2533Pro | EX47 | Het | missense |  |  |  |  |  |
| 22B02836804 | | chr19 | 39055854 | | 39055854 | | RYR1 | NM_000540.2 | c.12880A>G | p.Thr4294Ala | EX91 | Het | missense |  |  |  |  |  |
| 22B02836804 | | chr19 | 41354198 | | 41354198 | | CYP2A6 | NM_000762.5 | c.580A>G | p.Lys194Glu | EX4 | Het | missense | rs199916117 | 0.000808 | 0.002995 |  | 0.000617 |
| 22B02836804 | | chr19 | 44278567 | | 44278567 | | KCNN4 | NM_002250.2 | c.460C>T | p.(Leu154=) | EX3 | Het | coding-synon | rs564823653 | 0.000192 | 0.0002 |  | 0.000145 |
| 22B02836804 | | chr19 | 50100038 | | 50100038 | | PRR12 | NM_020719.1 | c.2446G>A | p.Ala816Thr | EX4 | Het | missense | rs200922630 | 0.000528 | 0.000399 |  | 0.000442 |
| 22B02836804 | | chr19 | 56539105 | | 56539105 | | NLRP5 | NM_153447.4 | c.1506C>T | p.(His502=) | EX7 | Het | coding-synon | rs144686764 | 0.000615 | 0.001597 |  | 0.000562 |
| 22B02836804 | | chr2 | 10582042 | | 10582042 | | ODC1 | NM_002539.1 | c.927G>A | p.(Ser309=) | EX10 | Het | coding-synon | rs778262198 | 0.000016 |  |  | 0.000012 |
| 22B02836804 | | chr2 | 26637210 | | 26637210 | | DRC1 | NM_145038.2 | c.156-2A>C |  | IVS1 | Het | splice-3 |  |  |  |  |  |
| 22B02836804 | | chr2 | 26697449 | | 26697449 | | OTOF | NM_194248.2 | c.3220G>A | p.Glu1074Lys | EX26 | Het | missense | rs768889857 | 0.000025 |  |  | 0.00004 |
| 22B02836804 | | chr2 | 33500037 | | 33500037 | | LTBP1 | NM_206943.2 | c.2749G>C | p.Asp917His | EX17 | Het | missense | rs142520374 | 0.000008 |  | 0.000077 | 0.000004 |
| 22B02836804 | | chr2 | 44099395 | | 44099395 | | ABCG8 | NM_022437.2 | c.1161G>A | p.(Pro387=) | EX8 | Het | coding-synon |  |  |  |  |  |
| 22B02836804 | | chr2 | 47641560 | | 47641563 | | MSH2 | NM_000251.2 | c.942+5_942+6delAA |  | IVS5 | Het | splice+10 |  |  |  |  |  |
| 22B02836804 | | chr2 | 86276118 | | 86276118 | | POLR1A | NM_015425.3 | c.2523G>A | p.(Glu841=) | EX18 | Het | coding-synon | rs557807677 | 0.000087 | 0.0002 |  | 0.000092 |
| 22B02836804 | | chr2 | 113509849 | | 113509849 | | CKAP2L | NM_152515.3 | c.1597G>A | p.Glu533Lys | EX5 | Het | missense | rs759793090 | 0.000016 |  |  | 0.000012 |
| **Sample ID** | | **#Chr** | **Start** | | **Stop** | | **Gene Symbol** | **Transcript** | **cHGVS** | **pHGVS** | **ExIn ID** | **Zygosity** | **Function** | **rsID** | **dbSNP Allele Freq** | **1000G AF** | **ESP6500 AF** | **GnomAD AF** |
| 22B02836804 | | chr2 | 170042195 | | 170042195 | | LRP2 | NM_004525.2 | c.9663C>T | p.(Leu3221=) | EX50 | Het | coding-synon | rs755099344 | 0.000008 |  |  | 0.00002 |
| 22B02836804 | | chr2 | 172314470 | | 172314470 | | DCAF17 | NM_025000.3 | c.628-11C>A |  | IVS6 | Het | splice-20 | rs375412538 | 0 |  |  | 0.000016 |
| 22B02836804 | | chr2 | 174223423 | | 174223423 | | CDCA7 | NM_031942.4 | c.22-17G>T |  | IVS1 | Het | splice-20 | rs113611019 | 0.006623 | 0.007388 | 0.003537 | 0.006182 |
| 22B02836804 | | chr2 | 176957650 | | 176957650 | | HOXD12 | NM_021193.3 | c.-6880G>C |  |  | Het | promoter | rs536639583 | 0.002208 | 0.002596 |  | 0.000185 |
| 22B02836804 | | chr2 | 176957650 | | 176957650 | | HOXD13 | NM_000523.3 | c.32G>C | p.Gly11Ala | EX1 | Het | missense | rs536639583 | 0.002208 | 0.002596 |  | 0.000185 |
| 22B02836804 | | chr2 | 179592487 | | 179592487 | | TTN | NM_133378.4 | c.16086A>G | p.(Lys5362=) | EX65 | Het | coding-synon | rs397517492 | 0.000167 | 0.000799 |  | 0.00008 |
| 22B02836804 | | chr2 | 179592487 | | 179592487 | | TTN | NM_001267550.1 | c.19818A>G | p.(Lys6606=) | EX68 | Het | coding-synon | rs397517492 | 0.000167 | 0.000799 |  | 0.00008 |
| 22B02836804 | | chr2 | 202625990 | | 202625990 | | ALS2 | NM_020919.3 | c.727A>T | p.Thr243Ser | EX4 | Het | missense |  |  |  |  |  |
| 22B02836804 | | chr2 | 219029628 | | 219029628 | | CXCR1 | NM_000634.2 | c.307T>C | p.Trp103Arg | EX2E | Het | missense | rs191545072 | 0.000237 | 0.001398 |  | 0.000247 |
| 22B02836804 | | chr2 | 219506741 | | 219506741 | | ZNF142 | NM_001105537.1 | c.4488+10G>C |  | IVS8 | Het | splice+10 | rs576599067 | 0.000314 | 0.000399 |  | 0.00032 |
| 22B02836804 | | chr2 | 219527284 | | 219527284 | | BCS1L | NM_004328.4 | c.771G>A | p.(Thr257=) | EX7 | Het | coding-synon | rs148302981 | 0.000066 |  | 0.000077 | 0.000048 |
| 22B02836804 | | chr2 | 219746929 | | 219746929 | | WNT10A | NM_025216.2 | c.160G>A | p.Val54Met | EX2 | Het | missense | rs375821607 | 0.000016 |  | 0.000077 | 0.000012 |
| 22B02836804 | | chr2 | 220421159 | | 220421159 | | OBSL1 | NM_015311.2 | c.4336+17G>A |  | IVS13 | Het | splice+20 | rs753310153 | 0.000134 |  |  | 0.000154 |
| 22B02836804 | | chr2 | 220435666 | | 220435666 | | OBSL1 | NM_015311.2 | c.289G>C | p.Ala97Pro | EX1 | Het | missense |  |  |  |  |  |
| 22B02836804 | | chr2 | 234665659 | | 234665659 | | UGT1A1 | NM_000463.2 | c.-3275T>G |  |  | Het | promoter | rs4124874 | 0.588059 | 0.588059 |  |  |
| 22B02836804 | | chr2 | 234669144 | | 234669144 | | UGT1A1 | NM_000463.2 | c.211G>A | p.Gly71Arg | EX1 | Het | missense | rs4148323 | 0.021206 | 0.034345 | 0.001307 | 0.022348 |
| 22B02836804 | | chr2 | 234676872 | | 234676872 | | UGT1A3 | NM_019093.2 | c.1094C>T | p.Pro365Leu | EX4 | Het | missense | rs34946978 | 0.001282 | 0.002196 | 0.000077 | 0.001241 |
| 22B02836804 | | chr2 | 234676872 | | 234676872 | | UGT1A4 | NM_007120.2 | c.1094C>T | p.Pro365Leu | EX4 | Het | missense | rs34946978 | 0.001282 | 0.002196 | 0.000077 | 0.001241 |
| 22B02836804 | | chr2 | 234676872 | | 234676872 | | UGT1A9 | NM_021027.2 | c.1082C>T | p.Pro361Leu | EX4 | Het | missense | rs34946978 | 0.001282 | 0.002196 | 0.000077 | 0.001241 |
| 22B02836804 | | chr2 | 234676872 | | 234676872 | | UGT1A5 | NM_019078.1 | c.1094C>T | p.Pro365Leu | EX4 | Het | missense | rs34946978 | 0.001282 | 0.002196 | 0.000077 | 0.001241 |
| 22B02836804 | | chr2 | 234676872 | | 234676872 | | UGT1A6 | NM_001072.3 | c.1088C>T | p.Pro363Leu | EX4 | Het | missense | rs34946978 | 0.001282 | 0.002196 | 0.000077 | 0.001241 |
| **Sample ID** | | **#Chr** | **Start** | | **Stop** | | **Gene Symbol** | **Transcript** | **cHGVS** | **pHGVS** | **ExIn ID** | **Zygosity** | **Function** | **rsID** | **dbSNP Allele Freq** | **1000G AF** | **ESP6500 AF** | **GnomAD AF** |
| 22B02836804 | | chr2 | 234676872 | | 234676872 | | UGT1A8 | NM_019076.4 | c.1082C>T | p.Pro361Leu | EX4 | Het | missense | rs34946978 | 0.001282 | 0.002196 | 0.000077 | 0.001241 |
| 22B02836804 | | chr2 | 234676872 | | 234676872 | | UGT1A1 | NM_000463.2 | c.1091C>T | p.Pro364Leu | EX4 | Het | missense | rs34946978 | 0.001282 | 0.002196 | 0.000077 | 0.001241 |
| 22B02836804 | | chr2 | 234676872 | | 234676872 | | UGT1A7 | NM_019077.2 | c.1082C>T | p.Pro361Leu | EX4 | Het | missense | rs34946978 | 0.001282 | 0.002196 | 0.000077 | 0.001241 |
| 22B02836804 | | chr2 | 234676872 | | 234676872 | | UGT1A10 | NM_019075.2 | c.1082C>T | p.Pro361Leu | EX4 | Het | missense | rs34946978 | 0.001282 | 0.002196 | 0.000077 | 0.001241 |
| 22B02836804 | | chr20 | 2841527 | | 2841527 | | VPS16 | NM_022575.2 | c.630+18G>A |  | IVS6 | Het | splice+20 | rs200732515 | 0.001125 | 0.000599 | 0.000231 | 0.001524 |
| 22B02836804 | | chr20 | 6750899 | | 6750899 | | BMP2 | NM_001200.2 | c.126A>C | p.(Ser42=) | EX2 | Het | coding-synon |  |  |  |  |  |
| 22B02836804 | | chr20 | 10621489 | | 10621489 | | JAG1 | NM_000214.2 | c.3141G>A | p.(Ser1047=) | EX25 | Het | coding-synon | rs202075581 | 0.000134 | 0.000799 |  | 0.000123 |
| 22B02836804 | | chr20 | 23346186 | | 23346186 | | GZF1 | NM_022482.3 | c.1166A>G | p.Lys389Arg | EX1 | Het | missense |  |  |  |  |  |
| 22B02836804 | | chr20 | 31374293 | | 31374293 | | DNMT3B | NM_006892.3 | c.307-15C>G |  | IVS4 | Het | splice-20 | rs150718732 | 0.000215 | 0.000399 |  | 0.000183 |
| 22B02836804 | | chr20 | 33330534 | | 33330534 | | NCOA6 | NM_014071.3 | c.3526A>G | p.Thr1176Ala | EX11 | Het | missense | rs186687743 | 0.000316 | 0.001198 | 0.000077 | 0.00031 |
| 22B02836804 | | chr20 | 47569233 | | 47569233 | | ARFGEF2 | NM_006420.2 | c.424-9C>A |  | IVS4 | Het | splice-10 |  |  |  |  |  |
| 22B02836804 | | chr20 | 48491352 | | 48491352 | | SLC9A8 | NM_001260491.1 | c.1117T>C | p.(Leu373=) | EX11 | Het | coding-synon | rs768601411 | 0.000082 |  |  | 0.000044 |
| 22B02836804 | | chr20 | 60892066 | | 60892066 | | LAMA5 | NM_005560.3 | c.7525G>T | p.Val2509Phe | EX56 | Het | missense | rs371152952 | 0.00002 |  | 0 | 0.000021 |
| 22B02836804 | | chr20 | 61451333 | | 61451333 | | COL9A3 | NM_001853.3 | c.308G>A | p.Arg103Gln | EX5 | Het | missense | rs142639450 | 0.01459 | 0.014577 | 0.009457 | 0.0136 |
| 22B02836804 | | chr20 | 62680168 | | 62680168 | | SOX18 | NM_018419.2 | c.506A>G | p.Glu169Gly | EX2E | Het | missense |  |  |  |  |  |
| 22B02836804 | | chr20 | 62680494 | | 62680494 | | SOX18 | NM_018419.2 | c.358+18G>A |  | IVS1 | Het | splice+20 | rs568521473 | 0.000091 | 0.000399 |  | 0.000039 |
| 22B02836804 | | chr21 | 28337776 | | 28337776 | | ADAMTS5 | NM_007038.3 | c.935G>T | p.Arg312Leu | EX1 | Het | missense | rs543666424 | 0.000222 | 0.000399 |  | 0.000255 |
| 22B02836804 | | chr22 | 19195771 | | 19195771 | | CLTCL1 | NM_007098.3 | c.3493C>T | p.Arg1165Cys | EX22 | Het | missense | rs190351859 | 0.005517 | 0.00639 | 0.001181 | 0.007414 |
| 22B02836804 | | chr22 | 19748523 | | 19748525 | | TBX1 | NM_080647.1 | c.143_145delCGC | p.Pro48del | EX3 | Het | cds-del |  |  |  |  | 0 |
| 22B02836804 | | chr22 | 20785078 | | 20785078 | | SCARF2 | NM_153334.4 | c.972C>A | p.(Gly324=) | EX5 | Het | coding-synon | rs77962729 | 0.000878 | 0.001797 |  | 0.000862 |
| 22B02836804 | | chr22 | 27012178 | | 27012178 | | CRYBB1 | NM_001887.3 | c.106G>A | p.Gly36Ser | EX2 | Het | missense | rs144659909 | 0.000079 | 0.000399 | 0.000231 | 0.000056 |
| **Sample ID** | | **#Chr** | **Start** | | **Stop** | | **Gene Symbol** | **Transcript** | **cHGVS** | **pHGVS** | **ExIn ID** | **Zygosity** | **Function** | **rsID** | **dbSNP Allele Freq** | **1000G AF** | **ESP6500 AF** | **GnomAD AF** |
| 22B02836804 | | chr22 | 50893151 | | 50893151 | | SBF1 | NM_002972.2 | c.4833C>T | p.(Asn1611=) | EX36 | Het | coding-synon | rs199573140 | 0.003046 | 0.001997 | 0.000746 | 0.003441 |
| 22B02836804 | | chr3 | 10417249 | | 10417249 | | ATP2B2 | NM_001001331.2 | c.1281G>A | p.(Pro427=) | EX11 | Het | coding-synon | rs113465029 | 0.000108 |  |  | 0.000108 |
| 22B02836804 | | chr3 | 14708427 | | 14708427 | | CCDC174 | NM_016474.4 | c.697G>A | p.Val233Ile | EX7 | Het | missense | rs145478230 | 0.000422 | 0.0002 | 0.000077 | 0.000433 |
| 22B02836804 | | chr3 | 15677019 | | 15677019 | | BTD | NM_000060.2 | c.133G>A | p.Gly45Arg | EX2 | Het | missense | rs34885143 | 0.009706 | 0.003794 | 0.011149 | 0.010182 |
| 22B02836804 | | chr3 | 38645235 | | 38645235 | | SCN5A | NM_198056.2 | c.1858C>T | p.Arg620Cys | EX12 | Het | missense | rs199473577 | 0.000009 |  |  | 0.000031 |
| 22B02836804 | | chr3 | 38648292 | | 38648292 | | SCN5A | NM_198056.2 | c.1008G>A | p.(Pro336=) | EX9 | Het | coding-synon | rs200285003 | 0.000252 | 0.000399 | 0.000241 | 0.00025 |
| 22B02836804 | | chr3 | 45583436 | | 45583436 | | LARS2 | NM_015340.3 | c.2520G>C | p.Gln840His | EX21 | Het | missense |  |  |  |  |  |
| 22B02836804 | | chr3 | 48621510 | | 48621510 | | COL7A1 | NM_000094.3 | c.4198-16G>A |  | IVS36 | Het | splice-20 | rs370515794 | 0.000143 | 0.0002 | 0.000154 | 0.000123 |
| 22B02836804 | | chr3 | 49136429 | | 49136435 | | QARS1 | NM_005051.1 | c.1759-13_1759-7delTCTCCTG |  | IVS18 | Het | splice-20 | rs781635166 | 0.000181 |  | 0.00024 | 0.000179 |
| 22B02836804 | | chr3 | 52378540 | | 52378540 | | DNAH1 | NM_015512.4 | c.1321G>C | p.Val441Leu | EX9 | Het | missense | rs13060192 | 0.03573 | 0.030551 | 0.03564 | 0.036747 |
| 22B02836804 | | chr3 | 52406116 | | 52406116 | | DNAH1 | NM_015512.4 | c.6666+14C>G |  | IVS42 | Het | splice+20 | rs147159692 | 0.001464 | 0.003195 | 0.000248 | 0.001282 |
| 22B02836804 | | chr3 | 52426833 | | 52426838 | | DNAH1 | NM_015512.4 | c.10279-13_10279-8delACTACA |  | IVS64 | Het | splice-20 | rs768374132 | 0.008297 |  |  | 0.003034 |
| 22B02836804 | | chr3 | 52429665 | | 52429665 | | DNAH1 | NM_015512.4 | c.11230C>T | p.Arg3744Cys | EX70 | Het | missense | rs419752 | 0.037775 | 0.029952 | 0.039314 | 0.037217 |
| 22B02836804 | | chr3 | 57132075 | | 57132075 | | IL17RD | NM_017563.3 | c.1656T>C | p.(Phe552=) | EX12 | Het | coding-synon | rs116882985 | 0.000459 | 0.001997 |  | 0.00047 |
| 22B02836804 | | chr3 | 58107156 | | 58107156 | | FLNB | NM_001457.3 | c.3052G>A | p.Val1018Met | EX20 | Het | missense | rs2276742 | 0.000325 | 0.000998 | 0.000615 | 0.000263 |
| 22B02836804 | | chr3 | 93845129 | | 93845129 | | NSUN3 | NM_022072.3 | c.818A>T | p.Gln273Leu | EX6E | Het | missense | rs779695970 | 0.00005 |  |  | 0.000044 |
| 22B02836804 | | chr3 | 119458151 | | 119458151 | | MAATS1 | NM_033364.3 | c.1511G>A | p.Arg504Gln | EX12 | Het | missense | rs375455508 | 0.000025 |  | 0.000077 | 0.000012 |
| 22B02836804 | | chr3 | 130289976 | | 130289976 | | COL6A6 | NM_001102608.1 | c.2716C>T | p.Arg906Cys | EX6 | Het | missense | rs200963433 | 0.000714 | 0.000799 |  | 0.000781 |
| 22B02836804 | | chr3 | 139063005 | | 139063005 | | MRPS22 | NM_020191.2 | c.137T>G | p.Met46Arg | EX1 | Het | missense |  |  |  |  |  |
| 22B02836804 | | chr3 | 148857868 | | 148857868 | | HPS3 | NM_032383.3 | c.295A>G | p.Thr99Ala | EX2 | Het | missense |  |  |  |  |  |
| 22B02836804 | | chr3 | 148899784 | | 148899784 | | CP | NM_000096.3 | c.2554+8C>G |  | IVS14 | Het | splice+10 | rs749646388 | 0.001121 |  |  | 0.000144 |
| **Sample ID** | | **#Chr** | **Start** | | **Stop** | | **Gene Symbol** | **Transcript** | **cHGVS** | **pHGVS** | **ExIn ID** | **Zygosity** | **Function** | **rsID** | **dbSNP Allele Freq** | **1000G AF** | **ESP6500 AF** | **GnomAD AF** |
| 22B02836804 | | chr3 | 170201230 | | 170201230 | | SLC7A14 | NM_020949.2 | c.988G>A | p.Gly330Arg | EX6 | Het | missense | rs2276717 | 0.001962 | 0.003395 | 0.000077 | 0.001726 |
| 22B02836804 | | chr3 | 184953161 | | 184953161 | | EHHADH | NM_001966.3 | c.268G>A | p.Val90Met | EX3 | Het | missense | rs56292788 | 0.000542 | 0.000998 | 0.000077 | 0.000518 |
| 22B02836804 | | chr3 | 186302391 | | 186302392 | | DNAJB11 | NM_016306.4 | c.1012+13_1012+14insTTGTGTGTGTGTGTGT |  | IVS9 | Het | splice+20 |  |  |  |  |  |
| 22B02836804 | | chr3 | 186302391 | | 186302392 | | DNAJB11 | NM_016306.4 | c.1012+13_1012+14insTTGTGTGTGTGTGT |  | IVS9 | Het | splice+20 |  |  |  |  |  |
| 22B02836804 | | chr3 | 186572419 | | 186572419 | | ADIPOQ | NM_001177800.1 | c.661C>A | p.Arg221Ser | EX4E | Het | missense | rs138773406 | 0.000239 | 0.000799 |  | 0.000207 |
| 22B02836804 | | chr4 | 5642254 | | 5642254 | | EVC2 | NM_147127.4 | c.1457G>T | p.Arg486Leu | EX10 | Het | missense |  |  |  | 0 |  |
| 22B02836804 | | chr4 | 10105534 | | 10105534 | | WDR1 | NM_017491.3 | c.215A>G | p.Tyr72Cys | EX3 | Het | missense |  |  |  |  | 0.000005 |
| 22B02836804 | | chr4 | 54966987 | | 54966987 | | GSX2 | NM_133267.2 | c.476C>G | p.Ala159Gly | EX1 | Het | missense |  |  |  |  |  |
| 22B02836804 | | chr4 | 56819340 | | 56819340 | | CEP135 | NM_025009.4 | c.203T>C | p.Leu68Ser | EX3 | Het | missense | rs147697562 | 0.000295 | 0.000799 |  | 0.000273 |
| 22B02836804 | | chr4 | 74315866 | | 74315866 | | AFP | NM_001134.1 | c.1289+16C>T |  | IVS10 | Het | splice+20 | rs201034665 | 0.00002 | 0.0002 |  | 0.000029 |
| 22B02836804 | | chr4 | 79428614 | | 79428614 | | FRAS1 | NM_025074.6 | c.9356A>G | p.Asn3119Ser | EX62 | Het | missense | rs191105001 | 0.000386 | 0.000599 | 0.000163 | 0.000327 |
| 22B02836804 | | chr4 | 96052611 | | 96052611 | | BMPR1B | NM_001203.2 | c.1024A>G | p.Lys342Glu | EX10 | Het | missense | rs748524936 | 0.000017 |  |  | 0.00002 |
| 22B02836804 | | chr4 | 100477318 | | 100477318 | | TRMT10A | NM_001134665.1 | c.480A>G | p.(Gly160=) | EX5 | Het | coding-synon |  |  |  |  |  |
| 22B02836804 | | chr4 | 122590897 | | 122590898 | | ANXA5 | NM_001154.3 | c.781-19_781-18insC |  | IVS11 | Het | splice-20 | rs781520875 | 0.000219 |  | 0.003459 | 0.000158 |
| 22B02836804 | | chr4 | 159601706 | | 159601706 | | ETFDH | NM_004453.2 | c.122G>A | p.Arg41Gln | EX2 | Het | missense | rs150105001 | 0.000058 |  | 0.000384 | 0.000064 |
| 22B02836804 | | chr4 | 185580578 | | 185580578 | | PRIMPOL | NM_152683.2 | c.265T>G | p.Tyr89Asp | EX4 | Het | missense | rs200857997 | 0.000467 | 0.001797 |  | 0.000442 |
| 22B02836804 | | chr5 | 256435 | | 256436 | | SDHA | NM_004168.2 | c.1909-14_1909-13delCT |  | IVS14 | Het | splice-20 | rs372662724 | 0.000008 |  |  | 0.000004 |
| 22B02836804 | | chr5 | 13919338 | | 13919338 | | DNAH5 | NM_001369.2 | c.922G>C | p.Asp308His | EX7 | Het | missense | rs553024984 | 0 | 0 |  | 0.000004 |
| 22B02836804 | | chr5 | 37019478 | | 37019478 | | NIPBL | NM_015384.4 | c.4986C>T | p.(Asn1662=) | EX25 | Het | coding-synon | rs754312670 | 0.000041 |  |  | 0.000068 |
| 22B02836804 | | chr5 | 38490321 | | 38490321 | | LIFR | NM_002310.5 | c.2138G>A | p.Arg713His | EX15 | Het | missense | rs766002119 | 0.000033 |  |  | 0.000036 |
| 22B02836804 | | chr5 | 70238373 | | 70238373 | | SMN1 | NM_000344.3 | c.462A>G | p.(Gln154=) | EX4 | Het | coding-synon | rs4915 | 0.805 |  |  |  |
| **Sample ID** | | **#Chr** | **Start** | | **Stop** | | **Gene Symbol** | **Transcript** | **cHGVS** | **pHGVS** | **ExIn ID** | **Zygosity** | **Function** | **rsID** | **dbSNP Allele Freq** | **1000G AF** | **ESP6500 AF** | **GnomAD AF** |
| 22B02836804 | | chr5 | 70247773 | | 70247773 | | SMN1 | NM_000344.3 | c.840C>T | p.(Phe280=) | EX8 | Het | coding-synon |  |  |  |  |  |
| 22B02836804 | | chr5 | 94872825 | | 94872825 | | TTC37 | NM_014639.3 | c.564T>C | p.(Asn188=) | EX9 | Het | coding-synon |  |  |  |  |  |
| 22B02836804 | | chr5 | 131539822 | | 131539822 | | P4HA2 | NM_004199.2 | c.1104T>C | p.(Asp368=) | EX9 | Het | coding-synon |  |  |  |  |  |
| 22B02836804 | | chr5 | 140026858 | | 140026858 | | NDUFA2 | NM_002488.4 | c.191A>G | p.Lys64Arg | EX2 | Het | missense | rs79526416 | 0.000155 | 0.000799 |  | 0.000138 |
| 22B02836804 | | chr5 | 145719315 | | 145719315 | | POU4F3 | NM_002700.2 | c.325C>T | p.His109Tyr | EX2E | Het | missense | rs754773365 | 0.000017 |  |  | 0.000012 |
| 22B02836804 | | chr5 | 150696654 | | 150696654 | | SLC36A2 | NM_181776.2 | c.1181-5T>C |  | IVS9 | Het | splice-10 | rs190486100 | 0.000572 | 0.001997 |  | 0.000512 |
| 22B02836804 | | chr5 | 150889748 | | 150889748 | | FAT2 | NM_001447.2 | c.11906-13C>A |  | IVS20 | Het | splice-20 | rs190441226 | 0.000474 | 0.001198 |  | 0.000454 |
| 22B02836804 | | chr5 | 167945076 | | 167945077 | | RARS1 | NM_002887.3 | c.1873+9_1873+10insTTTTTTTTTTTTT |  | IVS14 | Hom | splice+10 |  |  |  |  |  |
| 22B02836804 | | chr6 | 16327915 | | 16327915 | | ATXN1 | NM_000332.3 | c.627T>G | p.His209Gln | EX8 | Hom | missense | rs11969612 | 0.428571 |  |  |  |
| 22B02836804 | | chr6 | 24495286 | | 24495286 | | ALDH5A1 | NM_170740.1 | c.62G>A | p.Gly21Asp | EX1 | Het | missense | rs371923295 | 0.000423 |  |  | 0.00022 |
| 22B02836804 | | chr6 | 31238897 | | 31238897 | | HLA-C | NM_002117.5 | c.572G>C | p.Trp191Ser | EX3 | Het | missense | rs150127748 | 0.030456 |  |  | 0.014526 |
| 22B02836804 | | chr6 | 32557490 | | 32557490 | | HLA-DRB1 | NM_002124.3 | c.30C>T | p.(Ser10=) | EX1 | Het | coding-synon |  |  |  |  | 0 |
| 22B02836804 | | chr6 | 32557502 | | 32557502 | | HLA-DRB1 | NM_002124.3 | c.18C>G | p.(Leu6=) | EX1 | Het | coding-synon |  |  |  | 0 |  |
| 22B02836804 | | chr6 | 32557508 | | 32557508 | | HLA-DRB1 | NM_002124.3 | c.12G>C | p.(Leu4=) | EX1 | Het | coding-synon |  |  |  |  | 0 |
| 22B02836804 | | chr6 | 32629242 | | 32629243 | | HLA-DQB1 | NM_001243961.1 | c.662-9_662-8insTA |  | IVS3 | Het | splice-10 |  |  |  |  | 0 |
| 22B02836804 | | chr6 | 33419634 | | 33419634 | | SYNGAP1 | NM_006772.2 | c.3983G>C | p.Arg1328Pro | EX19E | Het | missense |  |  |  |  | 0.0019 |
| 22B02836804 | | chr6 | 33663494 | | 33663494 | | ITPR3 | NM_002224.3 | c.7953G>A | p.(Thr2651=) | EX58E | Het | coding-synon | rs147021135 | 0.000626 | 0.001198 | 0.000077 | 0.000585 |
| 22B02836804 | | chr6 | 45390510 | | 45390510 | | RUNX2 | NM_001024630.3 | c.239C>G | p.Ala80Gly | EX3 | Het | missense |  |  |  |  |  |
| 22B02836804 | | chr6 | 45390513 | | 45390513 | | RUNX2 | NM_001024630.3 | c.242C>G | p.Ala81Gly | EX3 | Het | missense |  |  |  |  |  |
| 22B02836804 | | chr6 | 45390516 | | 45390516 | | RUNX2 | NM_001024630.3 | c.245C>G | p.Ala82Gly | EX3 | Het | missense |  |  |  |  |  |
| 22B02836804 | | chr6 | 51918923 | | 51918923 | | PKHD1 | NM_138694.3 | c.1877A>G | p.Lys626Arg | EX20 | Het | missense | rs117122807 | 0.003252 | 0.008586 |  | 0.002837 |
| **Sample ID** | | **#Chr** | **Start** | | **Stop** | | **Gene Symbol** | **Transcript** | **cHGVS** | **pHGVS** | **ExIn ID** | **Zygosity** | **Function** | **rsID** | **dbSNP Allele Freq** | **1000G AF** | **ESP6500 AF** | **GnomAD AF** |
| 22B02836804 | | chr6 | 71003892 | | 71003892 | | COL9A1 | NM_001851.4 | c.674A>T | p.Asp225Val | EX5 | Het | missense | rs186444567 | 0.000277 | 0.000399 |  | 0.000255 |
| 22B02836804 | | chr6 | 108197875 | | 108197875 | | SEC63 | NM_007214.4 | c.1936-9delT |  | IVS18 | Het | splice-10 |  |  |  |  |  |
| 22B02836804 | | chr6 | 129511331 | | 129511331 | | LAMA2 | NM_000426.3 | c.1468-19T>C |  | IVS10 | Het | splice-20 | rs200241408 | 0.000309 | 0.001198 |  | 0.0003 |
| 22B02836804 | | chr6 | 142691874 | | 142691874 | | ADGRG6 | NM_020455.5 | c.1013A>T | p.Asn338Ile | EX4 | Het | missense | rs200437948 | 0.000303 | 0.000399 | 0.000084 | 0.000315 |
| 22B02836804 | | chr6 | 148835595 | | 148835596 | | SASH1 | NM_015278.3 | c.862+3_862+4delAA |  | IVS9 | Het | splice+10 |  |  |  |  | 0.005312 |
| 22B02836804 | | chr6 | 152708453 | | 152708453 | | SYNE1 | NM_033071.3 | c.8262G>C | p.Gln2754His | EX54 | Het | missense | rs200658991 | 0.000174 | 0.0002 |  | 0.000223 |
| 22B02836804 | | chr6 | 161807897 | | 161807897 | | PRKN | NM_004562.2 | c.1096C>T | p.Arg366Trp | EX10 | Het | missense | rs56092260 | 0.000269 | 0.001198 | 0.000308 | 0.000282 |
| 22B02836804 | | chr7 | 2962848 | | 2962848 | | CARD11 | NM_032415.4 | c.2060C>T | p.Ala687Val | EX16 | Het | missense | rs41493047 | 0.00101 | 0.000998 | 0.000846 | 0.00111 |
| 22B02836804 | | chr7 | 4827294 | | 4827294 | | AP5Z1 | NM_014855.2 | c.1341C>T | p.(Thr447=) | EX11 | Het | coding-synon | rs60284677 | 0.00047 | 0.0002 |  | 0.000334 |
| 22B02836804 | | chr7 | 8009069 | | 8009069 | | GLCCI1 | NM_138426.3 | c.88T>C | p.Ser30Pro | EX1 | Het | missense |  |  |  |  |  |
| 22B02836804 | | chr7 | 39991317 | | 39991331 | | CDK13 | NM_003718.4 | c.1077_1091delGAGCCCCTACAGTCG | p.Ser360_Arg364del | EX1 | Het | cds-del |  |  |  |  |  |
| 22B02836804 | | chr7 | 39991337 | | 39991339 | | CDK13 | NM_003718.4 | c.1097_1099delGCT | p.Arg366_Ser367delinsPro | EX1 | Het | cds-indel |  |  |  |  |  |
| 22B02836804 | | chr7 | 40234653 | | 40234653 | | SUGCT | NM_001193311.1 | c.499A>C | p.Ile167Leu | EX6 | Het | missense | rs138102615 | 0.001614 | 0.003594 |  | 0.001536 |
| 22B02836804 | | chr7 | 89938605 | | 89938605 | | CFAP69 | NM_001039706.2 | c.2579T>C | p.Ile860Thr | EX22 | Het | missense | rs758466834 | 0.000017 |  |  | 0.00004 |
| 22B02836804 | | chr7 | 117188684 | | 117188684 | | CFTR | NM_000492.3 | c.1210-11T>G |  | IVS9 | Het | splice-20 | rs73715573 | 0.010721 | 0.010383 | 0.003314 | 0.00861 |
| 22B02836804 | | chr7 | 127983715 | | 127983715 | | RBM28 | NM_018077.2 | c.118+15G>A |  | IVS1 | Het | splice+20 | rs200459017 | 0.000391 | 0.000998 |  | 0.000304 |
| 22B02836804 | | chr7 | 142457343 | | 142457343 | | PRSS1 | NM_002769.4 | c.8C>T | p.Pro3Leu | EX1 | Het | missense | rs374597855 | 0.000025 |  |  | 0 |
| 22B02836804 | | chr7 | 142457347 | | 142457347 | | PRSS1 | NM_002769.4 | c.12C>T | p.(Leu4=) | EX1 | Het | coding-synon | rs749968829 | 0.000025 |  |  | 0 |
| 22B02836804 | | chr7 | 142457365 | | 142457365 | | PRSS1 | NM_002769.4 | c.30G>T | p.(Val10=) | EX1 | Het | coding-synon | rs779260304 | 0.000008 |  |  |  |
| 22B02836804 | | chr7 | 142457375 | | 142457375 | | PRSS1 | NM_002769.4 | c.40C>G | p.Leu14Val | EX1 | Het | missense | rs747228052 | 0.000008 |  |  | 0.000004 |
| 22B02836804 | | chr7 | 142457382 | | 142457382 | | PRSS1 | NM_002769.4 | c.40+7A>T |  | IVS1 | Het | splice+10 | rs796173487 | 0 |  |  |  |
| **Sample ID** | | **#Chr** | **Start** | | **Stop** | | **Gene Symbol** | **Transcript** | **cHGVS** | **pHGVS** | **ExIn ID** | **Zygosity** | **Function** | **rsID** | **dbSNP Allele Freq** | **1000G AF** | **ESP6500 AF** | **GnomAD AF** |
| 22B02836804 | | chr7 | 142457386 | | 142457386 | | PRSS1 | NM_002769.4 | c.40+11T>C |  | IVS1 | Het | splice+20 | rs761465973 | 0.000008 |  |  | 0.000004 |
| 22B02836804 | | chr7 | 142458412 | | 142458412 | | PRSS1 | NM_002769.4 | c.47C>T | p.Ala16Val | EX2 | Het | missense | rs202003805 | 0.016039 |  |  | 0.00009 |
| 22B02836804 | | chr7 | 142458486 | | 142458486 | | PRSS1 | NM_002769.4 | c.121C>T | p.(Leu41=) | EX2 | Het | coding-synon | rs369646357 | 0.003751 |  |  | 0.000115 |
| 22B02836804 | | chr7 | 142459894 | | 142459894 | | PRSS1 | NM_002769.4 | c.454+16A>T |  | IVS3 | Het | splice+20 | rs377570765 | 0.000133 |  |  | 0.00002 |
| 22B02836804 | | chr7 | 142460379 | | 142460379 | | PRSS1 | NM_002769.4 | c.552C>T | p.(Phe184=) | EX4 | Het | coding-synon | rs767583768 | 0.007919 |  |  | 0 |
| 22B02836804 | | chr7 | 142460388 | | 142460388 | | PRSS1 | NM_002769.4 | c.561C>T | p.(Gly187=) | EX4 | Het | coding-synon | rs1804561 | 0.009654 |  |  | 0.000012 |
| 22B02836804 | | chr7 | 142460764 | | 142460764 | | PRSS1 | NM_002769.4 | c.637G>A | p.Val213Ile | EX5E | Het | missense | rs200902389 | 0.003234 |  |  | 0.000016 |
| 22B02836804 | | chr7 | 142460778 | | 142460778 | | PRSS1 | NM_002769.4 | c.651T>C | p.(Gly217=) | EX5E | Het | coding-synon | rs562372415 | 0.000079 | 0.0002 |  | 0.00004 |
| 22B02836804 | | chr7 | 142460779 | | 142460779 | | PRSS1 | NM_002769.4 | c.652G>T | p.Asp218Tyr | EX5E | Het | missense | rs574391339 | 0.000079 | 0.0002 |  | 0.00004 |
| 22B02836804 | | chr7 | 144095496 | | 144095496 | | NOBOX | NM_001080413.3 | c.1653T>C | p.(Leu551=) | EX9 | Het | coding-synon |  |  |  |  | 0.000035 |
| 22B02836804 | | chr7 | 150883497 | | 150883497 | | ASB10 | NM_080871.3 | c.521G>A | p.Arg174Gln | EX2 | Het | missense |  |  |  |  | 0.00001 |
| 22B02836804 | | chr7 | 151478406 | | 151478406 | | PRKAG2 | NM_016203.3 | c.298G>A | p.Gly100Ser | EX3 | Het | missense | rs79474211 | 0.008392 | 0.014577 | 0.000846 | 0.00736 |
| 22B02836804 | | chr7 | 151875096 | | 151875097 | | KMT2C | NM_170606.2 | c.7443-2_7443-1insA |  | IVS37 | Het | splice-3 | rs753425356 | 0.985332 |  |  | 0.000016 |
| 22B02836804 | | chr7 | 151875100 | | 151875101 | | KMT2C | NM_170606.2 | c.7443-6_7443-5insTTTTTTTTTA |  | IVS37 | Het | splice-10 |  |  |  |  |  |
| 22B02836804 | | chr7 | 151945228 | | 151945228 | | KMT2C | NM_170606.2 | c.2291C>T | p.Ser764Phe | EX14 | Het | missense | rs200184971 | 0.001076 |  |  | 0.000032 |
| 22B02836804 | | chr7 | 156802643 | | 156802644 | | MNX1 | NM_005515.3 | c.401_402insCGCCGCCGC | p.Ala134_Gly135insAlaAlaAla | EX1 | Hom | cds-ins |  |  |  |  |  |
| 22B02836804 | | chr8 | 1873490 | | 1873490 | | ARHGEF10 | NM_014629.2 | c.2530G>A | p.Gly844Arg | EX22 | Het | missense |  |  |  |  |  |
| 22B02836804 | | chr8 | 6302457 | | 6302457 | | MCPH1 | NM_024596.3 | c.1214T>C | p.Leu405Pro | EX8 | Het | missense | rs556803400 | 0.000589 | 0.000799 |  | 0.000665 |
| 22B02836804 | | chr8 | 8749306 | | 8749306 | | MFHAS1 | NM_004225.2 | c.1263A>G | p.(Gly421=) | EX1 | Het | coding-synon | rs746106122 | 0.000008 |  |  | 0.000012 |
| 22B02836804 | | chr8 | 10466476 | | 10466476 | | RP1L1 | NM_178857.5 | c.5132G>C | p.Gly1711Ala | EX4E | Het | missense | rs200635063 | 0.000517 | 0.001198 |  | 0.000493 |
| **Sample ID** | | **#Chr** | **Start** | | **Stop** | | **Gene Symbol** | **Transcript** | **cHGVS** | **pHGVS** | **ExIn ID** | **Zygosity** | **Function** | **rsID** | **dbSNP Allele Freq** | **1000G AF** | **ESP6500 AF** | **GnomAD AF** |
| 22B02836804 | | chr8 | 10467589 | | 10467590 | | RP1L1 | NM_178857.5 | c.4018_4019insGGACTAAAGTAATAGAAGGGCTGCAAGAAGAGAGGGTGCAGTTAGAGG | p.Glu1339_Glu1340insGlyThrLysValIleGluGlyLeuGlnGluGluArgValGlnLeuGlu | EX4E | Het | cds-ins |  |  |  |  |  |
| 22B02836804 | | chr8 | 10467637 | | 10467637 | | RP1L1 | NM_178857.5 | c.3971A>G | p.Glu1324Gly | EX4E | Het | missense |  |  |  |  |  |
| 22B02836804 | | chr8 | 10469386 | | 10469386 | | RP1L1 | NM_178857.5 | c.2222C>A | p.Thr741Asn | EX4E | Het | missense |  |  |  |  | 0.000004 |
| 22B02836804 | | chr8 | 10470838 | | 10470838 | | RP1L1 | NM_178857.5 | c.770C>G | p.Thr257Ser | EX4E | Het | missense |  |  |  |  | 0.000004 |
| 22B02836804 | | chr8 | 15978126 | | 15978134 | | MSR1 | NM_138715.2 | c.1034-12_1034-11delTT |  | IVS8 | Het | splice-20 |  |  |  |  |  |
| 22B02836804 | | chr8 | 20068093 | | 20068093 | | ATP6V1B2 | NM_001693.3 | c.399T>C | p.(Asn133=) | EX5 | Het | coding-synon |  |  |  |  |  |
| 22B02836804 | | chr8 | 21984575 | | 21984575 | | HR | NM_005144.4 | c.1380C>T | p.(Asp460=) | EX3 | Het | coding-synon | rs147308644 | 0.004607 | 0.006989 | 0.000236 | 0.005214 |
| 22B02836804 | | chr8 | 30700014 | | 30700014 | | TEX15 | NM_031271.3 | c.6520A>G | p.Lys2174Glu | EX1 | Het | missense | rs142315341 | 0.001806 | 0.003994 | 0.000077 | 0.002379 |
| 22B02836804 | | chr8 | 30700983 | | 30700983 | | TEX15 | NM_031271.3 | c.5551A>G | p.Lys1851Glu | EX1 | Het | missense |  |  |  |  |  |
| 22B02836804 | | chr8 | 48691059 | | 48691059 | | PRKDC | NM_006904.6 | c.11814C>T | p.(Ile3938=) | EX83 | Het | coding-synon | rs750761283 | 0.000018 |  |  | 0.000016 |
| 22B02836804 | | chr8 | 87588047 | | 87588047 | | CNGB3 | NM_019098.4 | c.2415A>C | p.Glu805Asp | EX18E | Het | missense | rs186448979 | 0.001158 | 0.003594 |  | 0.001075 |
| 22B02836804 | | chr8 | 87641127 | | 87641127 | | CNGB3 | NM_019098.4 | c.1480+20G>A |  | IVS12 | Het | splice+20 | rs117375929 | 0.000222 | 0.001597 |  | 0.000203 |
| 22B02836804 | | chr8 | 118825226 | | 118825226 | | EXT1 | NM_000127.2 | c.1633-26C>A |  | IVS7 | Het | intron | rs188609829 | 0.003338 | 0.004193 | 0.000154 | 0.002856 |
| 22B02836804 | | chr8 | 133877623 | | 133877623 | | TG | NM_003235.4 | c.-1623A>G |  |  | Het | promoter | rs180195 | 0.592452 | 0.592452 |  |  |
| 22B02836804 | | chr8 | 144990353 | | 144990353 | | PLEC | NM_000445.3 | c.13717G>A | p.Val4573Met | EX33E | Het | missense | rs573424409 | 0.000599 | 0.000599 |  | 0.000178 |
| 22B02836804 | | chr8 | 144994778 | | 144994778 | | PLEC | NM_000445.3 | c.9292G>A | p.Ala3098Thr | EX33E | Het | missense | rs782624968 | 0.000018 |  |  | 0.000008 |
| 22B02836804 | | chr8 | 145009203 | | 145009203 | | PLEC | NM_000445.3 | c.882C>T | p.(Asp294=) | EX9 | Het | coding-synon | rs202218097 | 0.000549 | 0.001198 | 0.000159 | 0.000597 |
| 22B02836804 | | chr9 | 2719083 | | 2719083 | | KCNV2 | NM_133497.3 | c.1344G>C | p.Trp448Cys | EX1 | Het | missense | rs143382624 | 0.000383 | 0.000799 |  | 0.000357 |
| 22B02836804 | | chr9 | 13176290 | | 13176290 | | MPDZ | NM_003829.4 | c.2776G>A | p.Ala926Thr | EX20 | Het | missense | rs144992780 | 0.000363 | 0.000998 |  | 0.000202 |
| **Sample ID** | | **#Chr** | **Start** | | **Stop** | | **Gene Symbol** | **Transcript** | **cHGVS** | **pHGVS** | **ExIn ID** | **Zygosity** | **Function** | **rsID** | **dbSNP Allele Freq** | **1000G AF** | **ESP6500 AF** | **GnomAD AF** |
| 22B02836804 | | chr9 | 16436068 | | 16436068 | | BNC2 | NM_017637.5 | c.2124C>T | p.(Ala708=) | EX6 | Het | coding-synon | rs145645768 | 0.000309 | 0.000998 |  | 0.000326 |
| 22B02836804 | | chr9 | 34512388 | | 34512388 | | DNAI1 | NM_012144.2 | c.1455G>A | p.(Thr485=) | EX15 | Het | coding-synon | rs200766993 | 0.000072 | 0.0002 |  | 0.000044 |
| 22B02836804 | | chr9 | 35068323 | | 35068323 | | VCP | NM_007126.3 | c.54A>G | p.(Lys18=) | EX2 | Het | coding-synon | rs766042571 | 0.00014 |  |  | 0.000159 |
| 22B02836804 | | chr9 | 35077114 | | 35077114 | | FANCG | NM_004629.1 | c.647-16C>A |  | IVS5 | Het | splice-20 | rs200107462 | 0.001171 | 0.001797 | 0.000077 | 0.001006 |
| 22B02836804 | | chr9 | 95237025 | | 95237030 | | ASPN | NM_017680.4 | c.147_152delTGATGA | p.Asp49_Asp50del | EX2 | Het | cds-del |  |  |  |  |  |
| 22B02836804 | | chr9 | 133556993 | | 133557007 | | PRDM12 | NM_021619.2 | c.1062_1076delCGCCGCCGCCGCCGC | p.Ala355_Ala359del | EX5E | Het | cds-del |  |  |  |  | 0 |
| 22B02836804 | | chr9 | 138589474 | | 138589474 | | SOHLH1 | NM_001012415.2 | c.346-1G>A |  | IVS3 | Het | splice-3 | rs140132974 | 0.003279 | 0.002995 | 0.000308 | 0.002897 |
| 22B02836804 | | chr9 | 138589474 | | 138589474 | | KCNT1 | NM_020822.2 | c.-4631C>T |  |  | Het | promoter | rs140132974 | 0.003279 | 0.002995 | 0.000308 | 0.002897 |
| 22B02836804 | | chr9 | 139311556 | | 139311556 | | PMPCA | NM_015160.1 | c.787C>G | p.Leu263Val | EX7 | Het | missense |  |  |  |  |  |
| 22B02836804 | | chr9 | 139329234 | | 139329234 | | INPP5E | NM_019892.4 | c.894C>T | p.(Asn298=) | EX2 | Het | coding-synon |  |  |  |  | 0.000032 |
| 22B02836804 | | chr9 | 139905532 | | 139905532 | | ABCA2 | NM_212533.2 | c.6039G>T | p.(Pro2013=) | EX39 | Het | coding-synon | rs200252232 | 0.000704 | 0.000399 | 0.000163 | 0.00061 |
| 22B02836804 | | chrX | 9656196 | | 9656196 | | TBL1X | NM_001139466.1 | c.497C>G | p.Ala166Gly | EX7 | Het | missense |  |  |  |  |  |
| 22B02836804 | | chrX | 38145910 | | 38145910 | | RPGR | NM_001034853.1 | c.2342C>G | p.Ala781Gly | EX15E | Het | missense |  |  |  | 0 |  |
| 22B02836804 | | chrX | 54275207 | | 54275207 | | WNK3 | NM_020922.4 | c.3574A>G | p.Ser1192Gly | EX17 | Hemi | missense |  |  |  |  |  |
| 22B02836804 | | chrX | 55051235 | | 55051235 | | ALAS2 | NM_000032.4 | c.220C>T | p.(Leu74=) | EX3 | Hemi | coding-synon | rs200307584 | 0.000404 | 0.00053 |  | 0.000414 |
| 22B02836804 | | chrX | 73811761 | | 73811761 | | RLIM | NM_016120.3 | c.1389C>A | p.(Ser463=) | EX4E | Het | coding-synon | rs773922290 | 0 |  |  | 0.000202 |
| 22B02836804 | | chrX | 74376092 | | 74376092 | | ABCB7 | NM_004299.3 | c.16A>G | p.Met6Val | EX1 | Hemi | missense |  |  |  |  | 0.000006 |
| 22B02836804 | | chrX | 76939281 | | 76939281 | | ATRX | NM_000489.3 | c.1467C>T | p.(Thr489=) | EX9 | Hemi | coding-synon | rs199929884 | 0.00024 | 0.000265 |  | 0.000312 |
| 22B02836804 | | chrX | 118985711 | | 118985712 | | UPF3B | NM_080632.2 | c.263+18_263+19insAAAAAAAAAA |  | IVS2 | Het | splice+20 |  |  |  |  |  |
| 22B02836804 | | chrX | 119077459 | | 119077459 | | NKAP | NM_024528.3 | c.110C>T | p.Pro37Leu | EX1 | Hemi | missense |  |  |  |  | 0.000006 |
| 22B02836804 | | chrX | 153008434 | | 153008434 | | ABCD1 | NM_000033.3 | c.1781-7C>T |  | IVS7 | Het | splice-10 | rs79915675 | 0.000166 |  |  | 0.000021 |
| **Sample ID** | | **#Chr** | **Start** | | **Stop** | | **Gene Symbol** | **Transcript** | **cHGVS** | **pHGVS** | **ExIn ID** | **Zygosity** | **Function** | **rsID** | **dbSNP Allele Freq** | **1000G AF** | **ESP6500 AF** | **GnomAD AF** |
| 22B02836804 | | chrX | 153008476 | | 153008476 | | ABCD1 | NM_000033.3 | c.1816T>C | p.Ser606Pro | EX8 | Het | missense | rs201774661 | 0.008522 |  |  | 0.000381 |
| 22B02836804 | | chrX | 153008483 | | 153008483 | | ABCD1 | NM_000033.3 | c.1823G>A | p.Gly608Asp | EX8 | Het | missense | rs78993751 | 0.008355 |  |  | 0.000409 |
| 22B02836804 | | chrX | 153008537 | | 153008537 | | ABCD1 | NM_000033.3 | c.1865+12G>A |  | IVS8 | Het | splice+20 | rs373638861 | 0.000279 |  |  | 0.000059 |
| 22B02836804 | | chrX | 153008542 | | 153008542 | | ABCD1 | NM_000033.3 | c.1865+17G>A |  | IVS8 | Het | splice+20 | rs377149542 | 0.00025 |  |  | 0.000022 |
| 22B02836804 | chr1 | | 1168188 | 1168188 | | B3GALT6 | | NM_080605.3 | c.530G>C | p.Arg177Pro | EX1E | Het | missense |  |  |  |  |  |
| 22B02836804 | chr1 | | 1447702 | 1447702 | | ATAD3A | | NM_018188.3 | c.54G>C | p.(Pro18=) | EX1 | Het | coding-synon |  |  |  |  |  |
| 22B02836804 | chr1 | | 1447873 | 1447877 | | ATAD3A | | NM_018188.3 | c.205+20_205+24delGCGGC |  | IVS1 | Het | splice+20 | rs761661834 | 0.004647 |  |  | 0.009069 |
| 22B02836804 | chr1 | | 2160390 | 2160391 | | SKI | | NM_003036.3 | c.189_194dupGGTGCC | p.Pro65_Ala66insValPro | EX1 | Het | cds-ins |  |  |  |  |  |
| 22B02836804 | chr1 | | 5993313 | 5993313 | | NPHP4 | | NM_015102.3 | c.1196A>G | p.Glu399Gly | EX10 | Het | missense | rs117898549 | 0.000826 | 0.002596 |  | 0.000744 |
| 22B02836804 | chr1 | | 6508836 | 6508836 | | ESPN | | NM_031475.2 | c.1600C>A | p.Arg534Ser | EX8 | Het | missense |  |  |  |  |  |
| 22B02836804 | chr1 | | 11856378 | 11856378 | | MTHFR | | NM_005957.4 | c.665C>T | p.Ala222Val | EX5 | Hom | missense | rs1801133 | 0.301359 | 0.245407 | 0.270567 | 0.314859 |
| 22B02836804 | chr1 | | 19204117 | 19204117 | | ALDH4A1 | | NM_170726.2 | c.941-11G>C |  | IVS9 | Het | splice-20 |  |  |  |  |  |
| 22B02836804 | chr1 | | 27240266 | 27240275 | | NR0B2 | | NM_021969.2 | c.157_166delCATCGCACCT | p.His53Alafs*50 | EX1 | Het | frameshift | rs540387719 | 0.000557 | 0.000799 |  | 0.000523 |
| 22B02836804 | chr1 | | 29323765 | 29323765 | | EPB41 | | NM_203342.2 | c.93T>C | p.(Cys31=) | EX5 | Het | coding-synon | rs756996105 | 0.000115 |  |  | 0.000099 |
| 22B02836804 | chr1 | | 31347439 | 31347439 | | SDC3 | | NM_014654.3 | c.871-4G>T |  | IVS3 | Het | splice-10 | rs188640187 | 0.005523 | 0.001398 | 0.000923 | 0.006507 |
| 22B02836804 | chr1 | | 31349557 | 31349557 | | SDC3 | | NM_014654.3 | c.712G>C | p.Val238Leu | EX3 | Het | missense | rs200515984 | 0.000308 | 0.000399 |  | 0.000235 |
| 22B02836804 | chr1 | | 33478900 | 33478900 | | AK2 | | NM_013411.4 | c.602A>T | p.Tyr201Phe | EX6 | Het | missense | rs113711467 | 0.00972 |  |  | 0.000215 |
| 22B02836804 | chr1 | | 35250901 | 35250901 | | GJB3 | | NM_024009.2 | c.538C>T | p.Arg180* | EX2E | Het | nonsense | rs74315319 | 0.000087 | 0.0002 |  | 0.000088 |
| 22B02836804 | chr1 | | 40557075 | 40557075 | | PPT1 | | NM_000310.3 | c.363-4G>A |  | IVS3 | Het | splice-10 | rs117284255 | 0.009286 | 0.009585 | 0.001076 | 0.010325 |
| 22B02836804 | chr1 | | 41284190 | 41284190 | | KCNQ4 | | NM_004700.3 | c.546C>G | p.Phe182Leu | EX4 | Het | missense | rs80358273 | 0.00034 | 0.000599 |  | 0.000329 |
| 22B02836804 | chr1 | | 47882174 | 47882174 | | FOXE3 | | NM_012186.2 | c.187C>G | p.Arg63Gly | EX1E | Het | missense |  |  |  |  |  |
| **Sample ID** | | **#Chr** | **Start** | | **Stop** | | **Gene Symbol** | **Transcript** | **cHGVS** | **pHGVS** | **ExIn ID** | **Zygosity** | **Function** | **rsID** | **dbSNP Allele Freq** | **1000G AF** | **ESP6500 AF** | **GnomAD AF** |
| 22B02836804 | chr1 | | 47882177 | 47882177 | | FOXE3 | | NM_012186.2 | c.190C>G | p.Arg64Gly | EX1E | Het | missense |  |  |  |  |  |
| 22B02836804 | chr1 | | 47882180 | 47882180 | | FOXE3 | | NM_012186.2 | c.193C>G | p.Arg65Gly | EX1E | Het | missense |  |  |  |  |  |
| 22B02836804 | chr1 | | 61872215 | 61872216 | | NFIA | | NM_001145511.1 | c.1231-19_1231-18insC |  | IVS8 | Het | splice-20 | rs759758399 | 0.073717 |  |  | 0.001845 |
| 22B02836804 | chr1 | | 68904775 | 68904775 | | RPE65 | | NM_000329.2 | c.859-11C>A |  | IVS8 | Het | splice-20 |  |  |  | 0 |  |
| 22B02836804 | chr1 | | 92446180 | 92446180 | | BRDT | | NM_001242806.1 | c.1300-20G>A |  | IVS8 | Het | splice-20 | rs201604833 | 0.000819 | 0.001398 |  | 0.00072 |
| 22B02836804 | chr1 | | 152276761 | 152276761 | | FLG | | NM_002016.1 | c.10601A>G | p.Asn3534Ser | EX3E | Het | missense | rs567405841 | 0.000396 | 0.004593 |  | 0.000214 |
| 22B02836804 | chr1 | | 152276762 | 152276762 | | FLG | | NM_002016.1 | c.10600A>C | p.Asn3534His | EX3E | Het | missense | rs12732870 | 0.000095 | 0.001797 |  | 0.000012 |
| 22B02836804 | chr1 | | 152278636 | 152278636 | | FLG | | NM_002016.1 | c.8726G>T | p.Gly2909Val | EX3E | Het | missense | rs542663583 | 0.000032 | 0.0002 |  | 0.000012 |
| 22B02836804 | chr1 | | 152281635 | 152281635 | | FLG | | NM_002016.1 | c.5727G>T | p.Arg1909Ser | EX3E | Het | missense | rs112627337 | 0.000008 |  | 0 | 0.000008 |
| 22B02836804 | chr1 | | 152284576 | 152284576 | | FLG | | NM_002016.1 | c.2786G>T | p.Gly929Val | EX3E | Het | missense | rs143382793 | 0.002949 |  |  | 0.000246 |
| 22B02836804 | chr1 | | 152284597 | 152284597 | | FLG | | NM_002016.1 | c.2765T>G | p.Ile922Ser | EX3E | Het | missense | rs201928359 | 0.001064 |  |  | 0.000004 |
| 22B02836804 | chr1 | | 152284603 | 152284604 | | FLG | | NM_002016.1 | c.2758_2759insGG | p.Ala920Glyfs*203 | EX3E | Het | frameshift | rs770547637 | 0.00098 |  |  | 0.000004 |
| 22B02836804 | chr1 | | 152284606 | 152284607 | | FLG | | NM_002016.1 | c.2755_2756delCA | p.His919Cysfs*5 | EX3E | Het | frameshift | rs759142251 | 0.000956 |  |  | 0.000004 |
| 22B02836804 | chr1 | | 165182873 | 165182873 | | LMX1A | | NM_177398.3 | c.669+5A>G |  | IVS5 | Het | splice+10 | rs144209134 | 0.000656 | 0.000799 |  | 0.000746 |
| 22B02836804 | chr1 | | 167802253 | 167802253 | | ADCY10 | | NM_018417.4 | c.3565C>A | p.(Arg1189=) | EX25 | Het | coding-synon | rs141732535 | 0.000633 | 0.002596 |  | 0.000525 |
| 22B02836804 | chr1 | | 176671882 | 176671885 | | PAPPA2 | | NM_020318.2 | c.3365+11_3365+14delTGTG |  | IVS9 | Het | splice+20 |  |  |  |  |  |
| 22B02836804 | chr1 | | 183086559 | 183086559 | | LAMC1 | | NM_002293.3 | c.1669C>T | p.Arg557Trp | EX9 | Het | missense | rs150392886 | 0.001963 | 0.005391 | 0.000077 | 0.001808 |
| 22B02836804 | chr1 | | 197059126 | 197059126 | | ASPM | | NM_018136.4 | c.9918T>C | p.(Cys3306=) | EX25 | Het | coding-synon |  |  |  |  |  |
| 22B02836804 | chr1 | | 200572995 | 200572995 | | KIF14 | | NM_014875.2 | c.1835C>T | p.Thr612Met | EX9 | Het | missense | rs747430628 | 0.000074 |  |  | 0.000111 |
| 22B02836804 | chr1 | | 207644254 | 207644254 | | CR2 | | NM_001006658.2 | c.1395A>G | p.(Gln465=) | EX7 | Het | coding-synon | rs188078143 | 0.000635 | 0.001597 | 0.000077 | 0.000626 |
| 22B02836804 | chr1 | | 207718903 | 207718903 | | CR1 | | NM_000651.4 | c.2414-18G>A |  | IVS14 | Hom | splice-20 | rs150543427 | 0.8 |  |  | 0 |
| **Sample ID** | | **#Chr** | **Start** | | **Stop** | | **Gene Symbol** | **Transcript** | **cHGVS** | **pHGVS** | **ExIn ID** | **Zygosity** | **Function** | **rsID** | **dbSNP Allele Freq** | **1000G AF** | **ESP6500 AF** | **GnomAD AF** |
| 22B02836804 | chr1 | | 210573930 | 210573930 | | HHAT | | NM_018194.4 | c.392G>C | p.Arg131Pro | EX5 | Het | missense | rs370012146 | 0.000404 | 0.0002 | 0 | 0.000251 |
| 22B02836804 | chr1 | | 220275746 | 220275746 | | IARS2 | | NM_018060.3 | c.741G>A | p.(Pro247=) | EX5 | Het | coding-synon | rs199601961 | 0.000468 | 0.001198 |  | 0.000395 |
| 22B02836804 | chr1 | | 224581555 | 224581555 | | WDR26 | | NM_025160.6 | c.1935T>C | p.(Pro645=) | EX13 | Het | coding-synon | rs142326612 | 0.000518 | 0.000399 |  | 0.000495 |
| 22B02836804 | chr1 | | 226027067 | 226027068 | | EPHX1 | | NM_001136018.2 | c.722+20_722+21insGTGT |  | IVS5 | Het | splice+20 | rs766429870 | 0 |  |  |  |
| 22B02836804 | chr1 | | 234743616 | 234743616 | | IRF2BP2 | | NM_182972.2 | c.1049-18A>G |  | IVS1 | Het | splice-20 | rs142257661 | 0.000378 | 0.002196 |  | 0.000309 |
| 22B02836804 | chr1 | | 241663903 | 241663904 | | FH | | NM_000143.3 | c.1237-14_1237-13delTC |  | IVS8 | Het | splice-20 |  |  |  |  |  |
| 22B02836804 | chr10 | | 94005 | 94005 | | TUBB8 | | NM_177987.2 | c.327C>T | p.(Gly109=) | EX4E | Het | coding-synon | rs374716232 | 0.002129 |  |  | 0.000277 |
| 22B02836804 | chr10 | | 94006 | 94006 | | TUBB8 | | NM_177987.2 | c.326G>T | p.Gly109Val | EX4E | Het | missense | rs368995010 | 0.001731 |  |  | 0.000008 |
| 22B02836804 | chr10 | | 94011 | 94011 | | TUBB8 | | NM_177987.2 | c.321C>A | p.(Thr107=) | EX4E | Het | coding-synon | rs782307404 | 0.007036 |  |  | 0.001519 |
| 22B02836804 | chr10 | | 13330389 | 13330389 | | PHYH | | NM_006214.3 | c.649C>G | p.Leu217Val | EX6 | Het | missense | rs200627042 | 0.000056 | 0.0002 |  | 0.000032 |
| 22B02836804 | chr10 | | 55626631 | 55626631 | | PCDH15 | | NM_033056.3 | c.3502-14delT |  | IVS26 | Het | splice-20 | rs530072775 | 0.004433 | 0.003594 | 0.007909 | 0.001891 |
| 22B02836804 | chr10 | | 71683573 | 71683573 | | COL13A1 | | NM_001130103.1 | c.1213G>T | p.Asp405Tyr | EX23 | Het | missense | rs117194484 | 0.000786 | 0.002196 | 0.000081 | 0.000608 |
| 22B02836804 | chr10 | | 72643718 | 72643719 | | PCBD1 | | NM_000281.2 | c.303_304delGT | p.Ser102Hisfs*10 | EX4E | Het | frameshift |  |  |  |  |  |
| 22B02836804 | chr10 | | 73466667 | 73466667 | | CDH23 | | NM_022124.5 | c.2967C>T | p.(Asn989=) | EX26 | Het | coding-synon | rs745617102 | 0.000038 |  |  | 0.000018 |
| 22B02836804 | chr10 | | 102789821 | 102789821 | | PDZD7 | | NM_001195263.1 | c.156C>T | p.(Asn52=) | EX2 | Het | coding-synon | rs150917752 | 0.001524 | 0.001997 | 0.000231 | 0.001324 |
| 22B02836804 | chr10 | | 104835985 | 104835985 | | CNNM2 | | NM_017649.4 | c.2376C>T | p.(Pro792=) | EX7 | Het | coding-synon | rs573050677 | 0.000228 | 0.000599 |  | 0.000122 |
| 22B02836804 | chr10 | | 114925413 | 114925413 | | TCF7L2 | | NM_030756.4 | c.1473G>C | p.(Pro491=) | EX14E | Het | coding-synon |  |  |  |  | 0.00008 |
| 22B02836804 | chr10 | | 114925414 | 114925414 | | TCF7L2 | | NM_030756.4 | c.1474A>C | p.Asn492His | EX14E | Het | missense |  |  |  |  | 0 |
| 22B02836804 | chr10 | | 118306934 | 118306934 | | PNLIP | | NM_000936.2 | c.175A>G | p.Thr59Ala | EX3 | Het | missense | rs147154871 | 0.000406 | 0.001797 |  | 0.000375 |
| 22B02836804 | chr10 | | 124810705 | 124810705 | | ACADSB | | NM_001609.3 | c.1128+3delA |  | IVS9 | Het | splice+10 |  |  |  |  |  |
| 22B02836804 | chr10 | | 131665499 | 131665499 | | EBF3 | | NM_001005463.2 | c.918C>T | p.(Thr306=) | EX10 | Het | coding-synon | rs76892753 | 0.000144 | 0.0002 |  | 0.000143 |
| **Sample ID** | | **#Chr** | **Start** | | **Stop** | | **Gene Symbol** | **Transcript** | **cHGVS** | **pHGVS** | **ExIn ID** | **Zygosity** | **Function** | **rsID** | **dbSNP Allele Freq** | **1000G AF** | **ESP6500 AF** | **GnomAD AF** |
| 22B02836804 | chr10 | | 135103311 | 135103312 | | TUBGCP2 | | NM_001256617.1 | c.1444+16_1444+17insAGGGACTTGGCGTCCGCGGCGCAGCGGGC |  | IVS10 | Hom | splice+20 |  |  |  |  |  |
| 22B02836804 | chr11 | | 613363 | 613363 | | IRF7 | | NM_001572.3 | c.1080G>A | p.(Glu360=) | EX9 | Het | coding-synon | rs766831508 | 0.000018 |  |  | 0.000025 |
| 22B02836804 | chr11 | | 639650 | 639651 | | DRD4 | | NM_000797.3 | c.417_418insGCCGTG | p.Val139_Pro140insAlaVal | EX3 | Het | cds-ins | rs751106635 | 0.998977 |  |  | 0.000952 |
| 22B02836804 | chr11 | | 1260145 | 1260145 | | MUC5B | | NM_002458.2 | c.3342T>C | p.(Cys1114=) | EX26 | Het | coding-synon | rs75799984 | 0.5 |  |  |  |
| 22B02836804 | chr11 | | 1260157 | 1260157 | | MUC5B | | NM_002458.2 | c.3354G>C | p.(Ser1118=) | EX26 | Het | coding-synon |  |  |  |  |  |
| 22B02836804 | chr11 | | 1260160 | 1260160 | | MUC5B | | NM_002458.2 | c.3357T>G | p.(Gly1119=) | EX26 | Het | coding-synon |  |  |  |  |  |
| 22B02836804 | chr11 | | 1260163 | 1260163 | | MUC5B | | NM_002458.2 | c.3360C>T | p.(Gly1120=) | EX26 | Het | coding-synon | rs79220674 | 0.000266 | 0.000399 |  | 0.000053 |
| 22B02836804 | chr11 | | 1260175 | 1260175 | | MUC5B | | NM_002458.2 | c.3372T>C | p.(Cys1124=) | EX26 | Het | coding-synon | rs75760167 | 1 |  |  |  |
| 22B02836804 | chr11 | | 1260193 | 1260193 | | MUC5B | | NM_002458.2 | c.3390T>C | p.(Ala1130=) | EX26 | Het | coding-synon |  |  |  |  |  |
| 22B02836804 | chr11 | | 1260214 | 1260214 | | MUC5B | | NM_002458.2 | c.3411C>T | p.(His1137=) | EX26 | Het | coding-synon | rs79585387 | 0.5 |  |  |  |
| 22B02836804 | chr11 | | 1260217 | 1260217 | | MUC5B | | NM_002458.2 | c.3414C>A | p.Asp1138Glu | EX26 | Het | missense |  |  |  |  | 0 |
| 22B02836804 | chr11 | | 1260219 | 1260219 | | MUC5B | | NM_002458.2 | c.3416C>T | p.Ala1139Val | EX26 | Het | missense | rs75935363 | 0.000041 |  |  | 0.000056 |
| 22B02836804 | chr11 | | 1260220 | 1260220 | | MUC5B | | NM_002458.2 | c.3417G>A | p.(Ala1139=) | EX26 | Het | coding-synon | rs61733358 | 0.000286 |  | 0 | 0.000126 |
| 22B02836804 | chr11 | | 1260244 | 1260244 | | MUC5B | | NM_002458.2 | c.3441T>C | p.(Thr1147=) | EX26 | Het | coding-synon | rs76590120 | 0.5 |  |  |  |
| 22B02836804 | chr11 | | 1260248 | 1260248 | | MUC5B | | NM_002458.2 | c.3445G>A | p.Asp1149Asn | EX26 | Het | missense |  |  |  |  |  |
| 22B02836804 | chr11 | | 1260249 | 1260249 | | MUC5B | | NM_002458.2 | c.3446A>G | p.Asp1149Gly | EX26 | Het | missense | rs77875446 | 0.5 |  |  |  |
| 22B02836804 | chr11 | | 1260252 | 1260252 | | MUC5B | | NM_002458.2 | c.3449C>T | p.Thr1150Ile | EX26 | Het | missense | rs78681629 | 0.5 |  |  |  |
| 22B02836804 | chr11 | | 1275978 | 1275978 | | MUC5B | | NM_002458.2 | c.15532G>C | p.Val5178Leu | EX35 | Het | missense | rs371813527 | 0.000097 | 0.000399 | 0.000078 | 0.000065 |
| 22B02836804 | chr11 | | 20622736 | 20622736 | | SLC6A5 | | NM_004211.3 | c.65C>G | p.Ala22Gly | EX2 | Het | missense |  |  |  |  |  |
| 22B02836804 | chr11 | | 20673907 | 20673907 | | SLC6A5 | | NM_004211.3 | c.2143A>G | p.Met715Val | EX15 | Het | missense | rs140461634 | 0.000008 |  | 0.000077 | 0.000004 |
| **Sample ID** | | **#Chr** | **Start** | | **Stop** | | **Gene Symbol** | **Transcript** | **cHGVS** | **pHGVS** | **ExIn ID** | **Zygosity** | **Function** | **rsID** | **dbSNP Allele Freq** | **1000G AF** | **ESP6500 AF** | **GnomAD AF** |
| 22B02836804 | chr11 | | 47296401 | 47296401 | | MADD | | NM_003682.3 | c.350G>A | p.Arg117His | EX3 | Het | missense | rs77637447 | 0.000143 | 0.000599 |  | 0.000167 |
| 22B02836804 | chr11 | | 61725599 | 61725600 | | BEST1 | | NM_004183.3 | c.715-19_715-18insTCCTCCTCC |  | IVS6 | Hom | splice-20 | rs113492158 | 0.5 |  |  |  |
| 22B02836804 | chr11 | | 67763203 | 67763203 | | UNC93B1 | | NM_030930.2 | c.1242G>C | p.(Leu414=) | EX9 | Het | coding-synon |  |  |  |  | 0.000026 |
| 22B02836804 | chr11 | | 67763229 | 67763229 | | UNC93B1 | | NM_030930.2 | c.1216G>A | p.Val406Met | EX9 | Het | missense | rs778453022 | 0 |  |  | 0.000007 |
| 22B02836804 | chr11 | | 67763230 | 67763230 | | UNC93B1 | | NM_030930.2 | c.1215G>C | p.(Leu405=) | EX9 | Het | coding-synon | rs1055846 | 0 |  |  | 0.000007 |
| 22B02836804 | chr11 | | 67811694 | 67811694 | | TCIRG1 | | NM_006019.3 | c.903C>T | p.(Ala301=) | EX9 | Het | coding-synon | rs773632286 | 0.000229 |  |  | 0.000063 |
| 22B02836804 | chr11 | | 68207394 | 68207394 | | LRP5 | | NM_002335.2 | c.4488+10G>A |  | IVS21 | Het | splice+10 | rs202206612 | 0.000849 | 0.000998 |  | 0.000402 |
| 22B02836804 | chr11 | | 68566672 | 68566672 | | CPT1A | | NM_001031847.2 | c.693+14C>T |  | IVS6 | Het | splice+20 | rs202064999 | 0.000016 | 0.0002 |  | 0.000024 |
| 22B02836804 | chr11 | | 68566797 | 68566797 | | CPT1A | | NM_001031847.2 | c.582G>T | p.Met194Ile | EX6 | Het | missense |  |  |  |  |  |
| 22B02836804 | chr11 | | 70858365 | 70858365 | | SHANK2 | | NM_012309.3 | c.8G>A | p.Arg3His | EX2 | Het | missense | rs369450251 | 0.00043 | 0.001398 | 0.000219 | 0.000278 |
| 22B02836804 | chr11 | | 78516320 | 78516321 | | TENM4 | | NM_001098816.2 | c.2179+16_2179+17insGAAGGGTGGGTGGAGGAGGAGTGGGGGTGGGGAAGAATGAGGGGTGGGGTGTGGAGGGTGGGGAG |  | IVS15 | Hom | splice+20 |  |  |  |  |  |
| 22B02836804 | chr11 | | 84865604 | 84865606 | | DLG2 | | NM_001142699.1 | c.276_278delGAC | p.Thr94del | EX5 | Het | cds-del |  |  |  |  | 0.000013 |
| 22B02836804 | chr11 | | 88045646 | 88045646 | | CTSC | | NM_001814.4 | c.395G>A | p.Arg132Gln | EX3 | Het | missense | rs575727793 | 0.000134 | 0.0002 |  | 0.000131 |
| 22B02836804 | chr11 | | 108181031 | 108181031 | | ATM | | NM_000051.3 | c.5907T>G | p.Asp1969Glu | EX39 | Het | missense |  |  |  |  |  |
| 22B02836804 | chr11 | | 112099026 | 112099026 | | PTS | | NM_000317.2 | c.84-291A>G |  | IVS1 | Het | intron |  |  |  |  |  |
| 22B02836804 | chr11 | | 116660952 | 116660955 | | APOA5 | | NM_052968.4 | c.990_993delAACA | p.Asp332Valfs*5 | EX4E | Het | frameshift | rs774150500 | 0.000008 |  |  | 0.000008 |
| 22B02836804 | chr11 | | 116660952 | 116660955 | | ZNF259 | | NM_003904.3 | c.-2249_-2246delAACA |  |  | Het | promoter | rs774150500 | 0.000008 |  |  | 0.000008 |
| 22B02836804 | chr11 | | 122954390 | 122954390 | | CLMP | | NM_024769.2 | c.554T>C | p.Ile185Thr | EX4 | Het | missense | rs537923955 | 0.000032 | 0.0002 |  | 0.000028 |
| 22B02836804 | chr11 | | 124745919 | 124745919 | | ROBO3 | | NM_022370.3 | c.2491A>G | p.Met831Val | EX16 | Het | missense |  |  |  |  |  |
| **Sample ID** | | **#Chr** | **Start** | | **Stop** | | **Gene Symbol** | **Transcript** | **cHGVS** | **pHGVS** | **ExIn ID** | **Zygosity** | **Function** | **rsID** | **dbSNP Allele Freq** | **1000G AF** | **ESP6500 AF** | **GnomAD AF** |
| 22B02836804 | chr12 | | 6167131 | 6167131 | | VWF | | NM_000552.3 | c.1613C>T | p.Pro538Leu | EX14 | Het | missense | rs139196998 | 0.000386 | 0.000599 | 0.000077 | 0.000395 |
| 22B02836804 | chr12 | | 6701717 | 6701717 | | CHD4 | | NM_001273.2 | c.2790T>C | p.(Phe930=) | EX19 | Het | coding-synon |  |  |  |  | 0.000004 |
| 22B02836804 | chr12 | | 7045891 | 7045907 | | ATN1 | | NM_001007026.1 | c.1508_1509insGCAGCA | p.Gln502_His503insGlnGln | EX5 | Het | cds-ins |  |  |  |  |  |
| 22B02836804 | chr12 | | 7045892 | 7045906 | | ATN1 | | NM_001007026.1 | c.1494_1508delGCAGCAGCAGCAGCA | p.Gln498_Gln502del | EX5 | Het | cds-del | rs377147612 | 0 |  |  |  |
| 22B02836804 | chr12 | | 14829843 | 14829843 | | GUCY2C | | NM_004963.3 | c.893C>T | p.Thr298Met | EX7 | Het | missense | rs148946391 | 0.001151 | 0.001398 | 0.000692 | 0.001047 |
| 22B02836804 | chr12 | | 14994015 | 14994015 | | ART4 | | NM_021071.2 | c.217G>A | p.Val73Ile | EX2 | Het | missense | rs182981694 | 0.000737 | 0.000998 | 0.000077 | 0.000709 |
| 22B02836804 | chr12 | | 31255366 | 31255367 | | DDX11 | | NM_030653.3 | c.2285_2296dupAGAGAGGCCAGG | p.Gln765_Val766insGluArgGlyGln | EX23 | Het | cds-ins |  |  |  |  |  |
| 22B02836804 | chr12 | | 40677699 | 40677699 | | LRRK2 | | NM_198578.3 | c.2264C>T | p.Pro755Leu | EX19 | Het | missense | rs34410987 | 0.000729 | 0.001597 |  | 0.000741 |
| 22B02836804 | chr12 | | 46245593 | 46245593 | | ARID2 | | NM_152641.2 | c.3687A>G | p.(Ser1229=) | EX15 | Het | coding-synon | rs76884235 | 0.000048 | 0.000399 |  | 0.00002 |
| 22B02836804 | chr12 | | 52710240 | 52710240 | | KRT83 | | NM_002282.3 | c.1041+12C>T |  | IVS6 | Het | splice+20 | rs201756344 | 0.000222 | 0.000399 | 0.000077 | 0.000259 |
| 22B02836804 | chr12 | | 52908879 | 52908879 | | KRT5 | | NM_000424.3 | c.1620C>T | p.(Val540=) | EX9E | Het | coding-synon | rs202197926 | 0.000308 | 0.000399 |  | 0.000375 |
| 22B02836804 | chr12 | | 56137184 | 56137185 | | GDF11 | | NM_005811.3 | c.116_117insGGC | p.Ala41_Gly42insAla | EX1 | Het | cds-ins | rs759951553 | 0 |  |  | 0 |
| 22B02836804 | chr12 | | 56743250 | 56743250 | | STAT2 | | NM_005419.3 | c.1301C>T | p.Thr434Met | EX15 | Het | missense | rs146115536 | 0.000049 |  | 0.000154 | 0.00008 |
| 22B02836804 | chr12 | | 109924386 | 109924386 | | UBE3B | | NM_130466.3 | c.447+6delA |  | IVS6 | Het | splice+10 |  |  |  |  |  |
| 22B02836804 | chr13 | | 25467010 | 25467011 | | CENPJ | | NM_018451.4 | c.2992-6_2992-5insTT |  | IVS9 | Het | splice-10 | rs746136069 | 0.782814 |  |  | 0.001601 |
| 22B02836804 | chr13 | | 31835236 | 31835236 | | B3GLCT | | NM_194318.3 | c.596+17A>T |  | IVS7 | Het | splice+20 | rs751928458 | 0.000008 |  |  | 0.001905 |
| 22B02836804 | chr13 | | 33590493 | 33590597 | | KL | | NM_004795.3 | c.-86_19del |  | .-EX1 | Het | span |  |  |  |  |  |
| 22B02836804 | chr13 | | 35733806 | 35733806 | | NBEA | | NM_015678.4 | c.3498C>T | p.(His1166=) | EX22 | Het | coding-synon | rs146937597 | 0.000095 | 0.000599 |  | 0.000049 |
| 22B02836804 | chr13 | | 52511626 | 52511626 | | ATP7B | | NM_000053.3 | c.3889G>A | p.Val1297Ile | EX18 | Het | missense | rs148399850 | 0.002298 | 0.003395 | 0.000471 | 0.002397 |
| 22B02836804 | chr13 | | 78492599 | 78492599 | | EDNRB | | NM_000115.3 | c.110C>T | p.Thr37Ile | EX2 | Het | missense |  |  |  |  | 0.000005 |
| **Sample ID** | | **#Chr** | **Start** | | **Stop** | | **Gene Symbol** | **Transcript** | **cHGVS** | **pHGVS** | **ExIn ID** | **Zygosity** | **Function** | **rsID** | **dbSNP Allele Freq** | **1000G AF** | **ESP6500 AF** | **GnomAD AF** |
| 22B02836804 | chr14 | | 35476472 | 35476472 | | SRP54 | | NM_003136.3 | c.256-17T>A |  | IVS4 | Het | splice-20 | rs770515104 | 0.000025 |  |  | 0.000017 |
| 22B02836804 | chr14 | | 35497285 | 35497286 | | SRP54 | | NM_003136.3 | c.1328-9_1328-8insTC |  | IVS14 | Het | splice-10 | rs761221697 | 0.000602 |  |  | 0.000465 |
| 22B02836804 | chr14 | | 39560784 | 39560784 | | SEC23A | | NM_006364.2 | c.500T>C | p.Ile167Thr | EX5 | Het | missense | rs144765020 | 0.000704 | 0.0002 | 0.000308 | 0.000947 |
| 22B02836804 | chr14 | | 52495481 | 52495481 | | NID2 | | NM_007361.3 | c.2489G>A | p.Arg830Gln | EX11 | Het | missense | rs7144523 | 0.008759 | 0.017772 | 0.011226 | 0.008731 |
| 22B02836804 | chr14 | | 64467412 | 64467412 | | SYNE2 | | NM_182914.2 | c.3613G>A | p.Glu1205Lys | EX28 | Het | missense | rs375217349 | 0.000088 | 0.0002 | 0.000085 | 0.000064 |
| 22B02836804 | chr14 | | 66082743 | 66082743 | | FUT8 | | NM_178155.2 | c.251G>A | p.Arg84His | EX4 | Het | missense | rs757041953 | 0.000042 |  |  | 0.000032 |
| 22B02836804 | chr14 | | 70420190 | 70420190 | | SMOC1 | | NM_022137.5 | c.319A>C | p.Lys107Gln | EX3 | Het | missense | rs149628528 | 0.000269 | 0.000399 |  | 0.000263 |
| 22B02836804 | chr14 | | 89034383 | 89034383 | | ZC3H14 | | NM_024824.4 | c.80A>G | p.Asp27Gly | EX3 | Het | missense |  |  |  |  |  |
| 22B02836804 | chr14 | | 99641650 | 99641650 | | BCL11B | | NM_138576.2 | c.1523A>G | p.Glu508Gly | EX4E | Het | missense |  |  |  |  |  |
| 22B02836804 | chr15 | | 28228553 | 28228553 | | OCA2 | | NM_000275.2 | c.1441G>A | p.Ala481Thr | EX14 | Het | missense | rs74653330 | 0.00776 | 0.007987 | 0.001307 | 0.008382 |
| 22B02836804 | chr15 | | 37329154 | 37329154 | | MEIS2 | | NM_002399.3 | c.722G>T | p.Gly241Val | EX8 | Het | missense | rs777260011 | 0.000033 |  |  | 0.00005 |
| 22B02836804 | chr15 | | 43045166 | 43045166 | | TTBK2 | | NM_173500.3 | c.2278C>T | p.Pro760Ser | EX14 | Het | missense | rs117382379 | 0.000095 | 0.000399 |  | 0.000112 |
| 22B02836804 | chr15 | | 43903199 | 43903200 | | STRC | | NM_153700.2 | c.3307-18_3307-17delCA |  | IVS13 | Het | splice-20 | rs796838028 | 0 |  |  |  |
| 22B02836804 | chr15 | | 44858159 | 44858159 | | SPG11 | | NM_025137.3 | c.6892A>G | p.Ile2298Val | EX38 | Het | missense | rs147962000 | 0.001086 | 0.002196 |  | 0.001003 |
| 22B02836804 | chr15 | | 48786463 | 48786463 | | FBN1 | | NM_000138.4 | c.2678-12T>C |  | IVS22 | Het | splice-20 | rs200368037 | 0.00057 | 0.000399 |  | 0.000727 |
| 22B02836804 | chr15 | | 65490706 | 65490706 | | CILP | | NM_003613.3 | c.1918A>G | p.Thr640Ala | EX9E | Het | missense |  |  |  |  |  |
| 22B02836804 | chr15 | | 72260385 | 72260385 | | MYO9A | | NM_006901.3 | c.1926C>T | p.(Ala642=) | EX13 | Het | coding-synon | rs2306487 | 0.000317 | 0.000998 | 0.000154 | 0.000279 |
| 22B02836804 | chr15 | | 74473160 | 74473160 | | STRA6 | | NM_001142617.1 | c.1803C>T | p.(Ala601=) | EX18 | Het | coding-synon | rs2277607 | 0.000293 | 0.0002 |  | 0.000303 |
| 22B02836804 | chr15 | | 77329671 | 77329673 | | PSTPIP1 | | NM_003978.3 | c.*155_*169delAAAAAAAAAAAAAAA |  | EX15E-. | Het | span | rs147238110 | 0.871805 |  |  |  |
| 22B02836804 | chr16 | | 1204054 | 1204054 | | CACNA1H | | NM_021098.2 | c.299+18C>G |  | IVS2 | Het | splice+20 |  |  |  |  |  |
| 22B02836804 | chr16 | | 1547104 | 1547104 | | TELO2 | | NM_016111.3 | c.681G>C | p.Gln227His | EX4 | Het | missense | rs139365746 | 0.00042 | 0.000399 | 0 | 0.000367 |
| **Sample ID** | | **#Chr** | **Start** | | **Stop** | | **Gene Symbol** | **Transcript** | **cHGVS** | **pHGVS** | **ExIn ID** | **Zygosity** | **Function** | **rsID** | **dbSNP Allele Freq** | **1000G AF** | **ESP6500 AF** | **GnomAD AF** |
| 22B02836804 | chr16 | | 2153484 | 2153484 | | PKD1 | | NM_001009944.2 | c.8574C>G | p.(Gly2858=) | EX23 | Het | coding-synon | rs545527733 | 0.000153 | 0.000599 |  | 0.00011 |
| 22B02836804 | chr16 | | 9002233 | 9002233 | | USP7 | | NM_003470.2 | c.1236C>T | p.(Asp412=) | EX12 | Het | coding-synon | rs759732382 | 0.000008 |  |  | 0.000004 |
| 22B02836804 | chr16 | | 11375054 | 11375054 | | PRM1 | | NM_002761.2 | c.42A>G | p.(Arg14=) | EX1 | Het | coding-synon | rs187174862 | 0.00034 | 0.001198 |  | 0.000239 |
| 22B02836804 | chr16 | | 21214437 | 21214437 | | ZP2 | | NM_003460.1 | c.1099+9C>A |  | IVS10 | Het | splice+10 | rs187716957 | 0.000462 | 0.001198 |  | 0.000328 |
| 22B02836804 | chr16 | | 21747639 | 21747639 | | OTOA | | NM_144672.3 | c.2359G>T | p.Glu787* | EX21 | Het | nonsense | rs200988634 | 0.00122 |  |  | 0.000308 |
| 22B02836804 | chr16 | | 23197787 | 23197787 | | SCNN1G | | NM_001039.3 | c.195C>T | p.(Ala65=) | EX2 | Het | coding-synon |  |  |  |  |  |
| 22B02836804 | chr16 | | 23632742 | 23632742 | | PALB2 | | NM_024675.3 | c.3054G>C | p.Glu1018Asp | EX10 | Het | missense | rs183489969 | 0.000324 | 0.000799 |  | 0.000382 |
| 22B02836804 | chr16 | | 31120690 | 31120690 | | BCKDK | | NM_005881.2 | c.146C>A | p.Thr49Asn | EX2 | Het | missense |  |  |  |  |  |
| 22B02836804 | chr16 | | 48258198 | 48258198 | | ABCC11 | | NM_032583.3 | c.538G>A | p.Gly180Arg | EX5 | Hom | missense | rs17822931 | 0.228117 | 0.300919 | 0.098 | 0.223953 |
| 22B02836804 | chr16 | | 55513411 | 55513411 | | MMP2 | | NM_004530.4 | c.20G>A | p.Arg7Gln | EX1 | Het | missense | rs746268212 | 0.000074 |  |  | 0.000025 |
| 22B02836804 | chr16 | | 67867761 | 67867761 | | CENPT | | NM_025082.3 | c.2T>C | p.0? | EX4 | Het | init-loss |  |  |  |  | 0.000012 |
| 22B02836804 | chr16 | | 85952421 | 85952421 | | IRF8 | | NM_002163.2 | c.988+12C>T |  | IVS7 | Het | splice+20 | rs577957168 | 0.000089 | 0.0002 |  | 0.000111 |
| 22B02836804 | chr16 | | 85953846 | 85953849 | | IRF8 | | NM_002163.2 | c.1104+16_1104+19delTTTT |  | IVS8 | Het | splice+20 | rs749732730 | 0.000912 |  |  |  |
| 22B02836804 | chr16 | | 89986025 | 89986025 | | TUBB3 | | NM_001197181.1 | c.-2788T>C |  |  | Het | promoter | rs33932559 | 0.003499 | 0.008387 | 0.000154 | 0.003137 |
| 22B02836804 | chr16 | | 89986025 | 89986025 | | MC1R | | NM_002386.3 | c.359T>C | p.Ile120Thr | EX1E | Het | missense | rs33932559 | 0.003499 | 0.008387 | 0.000154 | 0.003137 |
| 22B02836804 | chr17 | | 5463148 | 5463148 | | NLRP1 | | NM_001033053.2 | c.868A>G | p.Arg290Gly | EX4 | Het | missense | rs201548869 | 0.000095 | 0.0002 |  | 0.000091 |
| 22B02836804 | chr17 | | 8790536 | 8790536 | | PIK3R5 | | NM_001142633.2 | c.1782G>A | p.(Glu594=) | EX12 | Het | coding-synon | rs200935575 | 0.000414 | 0.000998 |  | 0.000471 |
| 22B02836804 | chr17 | | 16842773 | 16842775 | | TBC1D27 | | XM_003846282.1 | c.-6957_-6955delTGA |  |  | Hom | promoter | rs150068036 | 0.5 |  |  |  |
| 22B02836804 | chr17 | | 16842773 | 16842775 | | TNFRSF13B | | NM_012452.2 | c.*86_*88delTGA |  | EX5E | Hom | utr-3 | rs150068036 | 0.5 |  |  |  |
| 22B02836804 | chr17 | | 18034132 | 18034133 | | MYO15A | | NM_016239.3 | c.4038+8_4038+9delGT |  | IVS8 | Het | splice+10 | rs529930336 | 0.234146 |  |  |  |
| 22B02836804 | chr17 | | 18077164 | 18077164 | | MYO15A | | NM_016239.3 | c.10420A>G | p.Ser3474Gly | EX65 | Het | missense | rs150181830 | 0.000534 | 0.000998 |  | 0.000508 |
| **Sample ID** | | **#Chr** | **Start** | | **Stop** | | **Gene Symbol** | **Transcript** | **cHGVS** | **pHGVS** | **ExIn ID** | **Zygosity** | **Function** | **rsID** | **dbSNP Allele Freq** | **1000G AF** | **ESP6500 AF** | **GnomAD AF** |
| 22B02836804 | chr17 | | 19568258 | 19568258 | | ALDH3A2 | | NM_000382.2 | c.1108-3C>T |  | IVS7 | Het | splice-10 | rs148944691 | 0.000555 | 0.000799 |  | 0.000621 |
| 22B02836804 | chr17 | | 26727703 | 26727703 | | SLC46A1 | | NM_080669.4 | c.1245A>T | p.(Pro415=) | EX4 | Het | coding-synon | rs782287467 | 0.00001 |  |  | 0.000004 |
| 22B02836804 | chr17 | | 29677247 | 29677247 | | NF1 | | NM_001042492.2 | c.7368A>G | p.(Lys2456=) | EX50 | Het | coding-synon | rs201287021 | 0.000411 | 0.000399 |  | 0.000497 |
| 22B02836804 | chr17 | | 29677247 | 29677247 | | NF1 | | NM_000267.3 | c.7305A>G | p.(Lys2435=) | EX49 | Het | coding-synon | rs201287021 | 0.000411 | 0.000399 |  | 0.000497 |
| 22B02836804 | chr17 | | 38487505 | 38487505 | | RARA | | NM_000964.3 | c.35G>A | p.Gly12Glu | EX2 | Het | missense |  |  |  | 0 |  |
| 22B02836804 | chr17 | | 41251778 | 41251778 | | BRCA1 | | NM_007294.3 | c.547+14delG |  | IVS7 | Het | splice+20 | rs273902771 | 0.000124 |  |  | 0.000088 |
| 22B02836804 | chr17 | | 42284747 | 42284747 | | UBTF | | NM_001076683.1 | c.2059-12C>T |  | IVS19 | Het | splice-20 |  |  |  |  | 0.000004 |
| 22B02836804 | chr17 | | 42340018 | 42340018 | | SLC4A1 | | NM_000342.3 | c.92T>C | p.Met31Thr | EX3 | Het | missense | rs55773290 | 0.000357 | 0.001398 |  | 0.000246 |
| 22B02836804 | chr17 | | 61497906 | 61497906 | | TANC2 | | NM_025185.3 | c.4563G>A | p.(Val1521=) | EX25E | Het | coding-synon | rs201592811 | 0.000016 | 0.0002 |  | 0 |
| 22B02836804 | chr17 | | 61972500 | 61972500 | | CSH1 | | NM_001317.5 | c.536A>G | p.His179Arg | EX5E | Het | missense | rs570114664 | 0.00004 | 0.000399 |  | 0.00002 |
| 22B02836804 | chr17 | | 71196850 | 71196850 | | COG1 | | NM_018714.2 | c.1216C>G | p.Leu406Val | EX6 | Het | missense |  |  |  |  |  |
| 22B02836804 | chr17 | | 73975026 | 73975026 | | ACOX1 | | NM_004035.6 | c.109+20C>G |  | IVS1 | Het | splice+20 | rs750088491 | 0.000017 |  |  | 0.00002 |
| 22B02836804 | chr17 | | 76457692 | 76457692 | | DNAH17 | | NM_173628.3 | c.9273C>T | p.(Ala3091=) | EX58 | Het | coding-synon | rs548700594 | 0.000358 | 0.000599 |  | 0.000216 |
| 22B02836804 | chr17 | | 78298891 | 78298891 | | RNF213 | | NM_001256071.1 | c.3086T>C | p.Leu1029Ser | EX18 | Het | missense | rs753208141 | 0.000114 |  |  | 0.000049 |
| 22B02836804 | chr17 | | 80863817 | 80863817 | | TBCD | | NM_005993.4 | c.1810C>T | p.Pro604Ser | EX20 | Het | missense | rs575721383 | 0.00073 | 0.000998 |  | 0.000507 |
| 22B02836804 | chr18 | | 10677880 | 10677881 | | PIEZO2 | | NM_022068.2 | c.7614-9_7614-8insT |  | IVS48 | Het | splice-10 | rs771100722 | 0.99063 | 0.004593 |  | 0.007677 |
| 22B02836804 | chr18 | | 21124907 | 21124908 | | NPC1 | | NM_000271.4 | c.1947+16_1947+17insGGGGGG |  | IVS12 | Het | splice+20 |  |  |  |  |  |
| 22B02836804 | chr18 | | 34297913 | 34297913 | | FHOD3 | | NM_025135.2 | c.2127T>C | p.(Asp709=) | EX16 | Het | coding-synon | rs3809993 | 0.000116 |  | 0.000077 | 0.000103 |
| 22B02836804 | chr18 | | 55247336 | 55247336 | | FECH | | NM_001012515.2 | c.163G>T | p.Gly55Cys | EX2 | Het | missense | rs3848519 | 0.02237 | 0.026957 | 0.018299 | 0.022062 |
| 22B02836804 | chr18 | | 77475368 | 77475368 | | CTDP1 | | NM_004715.4 | c.1908C>T | p.(Asp636=) | EX8 | Het | coding-synon | rs143177926 | 0.002675 | 0.007188 | 0.00592 | 0.001274 |
| 22B02836804 | chr19 | | 1218407 | 1218407 | | STK11 | | NM_000455.4 | c.291-9C>G |  | IVS1 | Het | splice-10 |  |  |  |  |  |
| **Sample ID** | | **#Chr** | **Start** | | **Stop** | | **Gene Symbol** | **Transcript** | **cHGVS** | **pHGVS** | **ExIn ID** | **Zygosity** | **Function** | **rsID** | **dbSNP Allele Freq** | **1000G AF** | **ESP6500 AF** | **GnomAD AF** |
| 22B02836804 | chr19 | | 4365494 | 4365494 | | SH3GL1 | | NM_003025.3 | c.316G>A | p.Gly106Ser | EX4 | Het | missense | rs539292999 | 0.000127 | 0.0002 |  | 0.000155 |
| 22B02836804 | chr19 | | 5131352 | 5131352 | | KDM4B | | NM_015015.2 | c.1581C>T | p.(Pro527=) | EX12 | Het | coding-synon | rs779398920 | 0.000017 |  |  | 0.000008 |
| 22B02836804 | chr19 | | 6707259 | 6707259 | | C3 | | NM_000064.2 | c.2073C>G | p.(Arg691=) | EX17 | Het | coding-synon |  |  |  |  |  |
| 22B02836804 | chr19 | | 8609201 | 8609201 | | MYO1F | | NM_012335.3 | c.1504A>G | p.Ile502Val | EX14 | Het | missense | rs200797032 | 0.000509 | 0.000799 |  | 0.000467 |
| 22B02836804 | chr19 | | 12775790 | 12775790 | | MAN2B1 | | NM_000528.3 | c.446A>C | p.Glu149Ala | EX4 | Het | missense |  |  |  |  |  |
| 22B02836804 | chr19 | | 13211778 | 13211778 | | LYL1 | | NM_005583.4 | c.208C>G | p.Pro70Ala | EX2 | Het | missense | rs552041622 | 0.000428 | 0.000399 |  | 0.000138 |
| 22B02836804 | chr19 | | 18271265 | 18271266 | | PIK3R2 | | NM_005027.2 | c.323-16_323-15delCT |  | IVS2 | Het | splice-20 | rs374033727 | 0.00025 | 0.000599 | 0.001757 | 0.000269 |
| 22B02836804 | chr19 | | 18705138 | 18705138 | | CRLF1 | | NM_004750.4 | c.1131C>A | p.His377Gln | EX7 | Het | missense |  |  |  |  |  |
| 22B02836804 | chr19 | | 19309985 | 19309985 | | RFXANK | | NM_003721.2 | c.654C>T | p.(Thr218=) | EX9 | Het | coding-synon | rs8862 | 0.000183 | 0.000599 | 0.000077 | 0.000167 |
| 22B02836804 | chr19 | | 33321587 | 33321587 | | SLC7A9 | | NM_014270.4 | c.1403C>T | p.Pro468Leu | EX13E | Het | missense | rs80283711 | 0.002794 | 0.004193 | 0.000154 | 0.003169 |
| 22B02836804 | chr19 | | 38991613 | 38991613 | | RYR1 | | NM_000540.2 | c.7597G>C | p.Ala2533Pro | EX47 | Het | missense |  |  |  |  |  |
| 22B02836804 | chr19 | | 39055854 | 39055854 | | RYR1 | | NM_000540.2 | c.12880A>G | p.Thr4294Ala | EX91 | Het | missense |  |  |  |  |  |
| 22B02836804 | chr19 | | 41354198 | 41354198 | | CYP2A6 | | NM_000762.5 | c.580A>G | p.Lys194Glu | EX4 | Het | missense | rs199916117 | 0.000808 | 0.002995 |  | 0.000617 |
| 22B02836804 | chr19 | | 44278567 | 44278567 | | KCNN4 | | NM_002250.2 | c.460C>T | p.(Leu154=) | EX3 | Het | coding-synon | rs564823653 | 0.000192 | 0.0002 |  | 0.000145 |
| 22B02836804 | chr19 | | 50100038 | 50100038 | | PRR12 | | NM_020719.1 | c.2446G>A | p.Ala816Thr | EX4 | Het | missense | rs200922630 | 0.000528 | 0.000399 |  | 0.000442 |
| 22B02836804 | chr19 | | 56539105 | 56539105 | | NLRP5 | | NM_153447.4 | c.1506C>T | p.(His502=) | EX7 | Het | coding-synon | rs144686764 | 0.000615 | 0.001597 |  | 0.000562 |
| 22B02836804 | chr2 | | 10582042 | 10582042 | | ODC1 | | NM_002539.1 | c.927G>A | p.(Ser309=) | EX10 | Het | coding-synon | rs778262198 | 0.000016 |  |  | 0.000012 |
| 22B02836804 | chr2 | | 26637210 | 26637210 | | DRC1 | | NM_145038.2 | c.156-2A>C |  | IVS1 | Het | splice-3 |  |  |  |  |  |
| 22B02836804 | chr2 | | 26697449 | 26697449 | | OTOF | | NM_194248.2 | c.3220G>A | p.Glu1074Lys | EX26 | Het | missense | rs768889857 | 0.000025 |  |  | 0.00004 |
| 22B02836804 | chr2 | | 33500037 | 33500037 | | LTBP1 | | NM_206943.2 | c.2749G>C | p.Asp917His | EX17 | Het | missense | rs142520374 | 0.000008 |  | 0.000077 | 0.000004 |
| 22B02836804 | chr2 | | 44099395 | 44099395 | | ABCG8 | | NM_022437.2 | c.1161G>A | p.(Pro387=) | EX8 | Het | coding-synon |  |  |  |  |  |
| **Sample ID** | | **#Chr** | **Start** | | **Stop** | | **Gene Symbol** | **Transcript** | **cHGVS** | **pHGVS** | **ExIn ID** | **Zygosity** | **Function** | **rsID** | **dbSNP Allele Freq** | **1000G AF** | **ESP6500 AF** | **GnomAD AF** |
| 22B02836804 | chr2 | | 47641560 | 47641563 | | MSH2 | | NM_000251.2 | c.942+5_942+6delAA |  | IVS5 | Het | splice+10 |  |  |  |  |  |
| 22B02836804 | chr2 | | 86276118 | 86276118 | | POLR1A | | NM_015425.3 | c.2523G>A | p.(Glu841=) | EX18 | Het | coding-synon | rs557807677 | 0.000087 | 0.0002 |  | 0.000092 |
| 22B02836804 | chr2 | | 113509849 | 113509849 | | CKAP2L | | NM_152515.3 | c.1597G>A | p.Glu533Lys | EX5 | Het | missense | rs759793090 | 0.000016 |  |  | 0.000012 |
| 22B02836804 | chr2 | | 170042195 | 170042195 | | LRP2 | | NM_004525.2 | c.9663C>T | p.(Leu3221=) | EX50 | Het | coding-synon | rs755099344 | 0.000008 |  |  | 0.00002 |
| 22B02836804 | chr2 | | 172314470 | 172314470 | | DCAF17 | | NM_025000.3 | c.628-11C>A |  | IVS6 | Het | splice-20 | rs375412538 | 0 |  |  | 0.000016 |
| 22B02836804 | chr2 | | 174223423 | 174223423 | | CDCA7 | | NM_031942.4 | c.22-17G>T |  | IVS1 | Het | splice-20 | rs113611019 | 0.006623 | 0.007388 | 0.003537 | 0.006182 |
| 22B02836804 | chr2 | | 176957650 | 176957650 | | HOXD12 | | NM_021193.3 | c.-6880G>C |  |  | Het | promoter | rs536639583 | 0.002208 | 0.002596 |  | 0.000185 |
| 22B02836804 | chr2 | | 176957650 | 176957650 | | HOXD13 | | NM_000523.3 | c.32G>C | p.Gly11Ala | EX1 | Het | missense | rs536639583 | 0.002208 | 0.002596 |  | 0.000185 |
| 22B02836804 | chr2 | | 179592487 | 179592487 | | TTN | | NM_133378.4 | c.16086A>G | p.(Lys5362=) | EX65 | Het | coding-synon | rs397517492 | 0.000167 | 0.000799 |  | 0.00008 |
| 22B02836804 | chr2 | | 179592487 | 179592487 | | TTN | | NM_001267550.1 | c.19818A>G | p.(Lys6606=) | EX68 | Het | coding-synon | rs397517492 | 0.000167 | 0.000799 |  | 0.00008 |
| 22B02836804 | chr2 | | 202625990 | 202625990 | | ALS2 | | NM_020919.3 | c.727A>T | p.Thr243Ser | EX4 | Het | missense |  |  |  |  |  |
| 22B02836804 | chr2 | | 219029628 | 219029628 | | CXCR1 | | NM_000634.2 | c.307T>C | p.Trp103Arg | EX2E | Het | missense | rs191545072 | 0.000237 | 0.001398 |  | 0.000247 |
| 22B02836804 | chr2 | | 219506741 | 219506741 | | ZNF142 | | NM_001105537.1 | c.4488+10G>C |  | IVS8 | Het | splice+10 | rs576599067 | 0.000314 | 0.000399 |  | 0.00032 |
| 22B02836804 | chr2 | | 219527284 | 219527284 | | BCS1L | | NM_004328.4 | c.771G>A | p.(Thr257=) | EX7 | Het | coding-synon | rs148302981 | 0.000066 |  | 0.000077 | 0.000048 |
| 22B02836804 | chr2 | | 219746929 | 219746929 | | WNT10A | | NM_025216.2 | c.160G>A | p.Val54Met | EX2 | Het | missense | rs375821607 | 0.000016 |  | 0.000077 | 0.000012 |
| 22B02836804 | chr2 | | 220421159 | 220421159 | | OBSL1 | | NM_015311.2 | c.4336+17G>A |  | IVS13 | Het | splice+20 | rs753310153 | 0.000134 |  |  | 0.000154 |
| 22B02836804 | chr2 | | 220435666 | 220435666 | | OBSL1 | | NM_015311.2 | c.289G>C | p.Ala97Pro | EX1 | Het | missense |  |  |  |  |  |
| 22B02836804 | chr2 | | 234665659 | 234665659 | | UGT1A1 | | NM_000463.2 | c.-3275T>G |  |  | Het | promoter | rs4124874 | 0.588059 | 0.588059 |  |  |
| 22B02836804 | chr2 | | 234669144 | 234669144 | | UGT1A1 | | NM_000463.2 | c.211G>A | p.Gly71Arg | EX1 | Het | missense | rs4148323 | 0.021206 | 0.034345 | 0.001307 | 0.022348 |
| 22B02836804 | chr2 | | 234676872 | 234676872 | | UGT1A3 | | NM_019093.2 | c.1094C>T | p.Pro365Leu | EX4 | Het | missense | rs34946978 | 0.001282 | 0.002196 | 0.000077 | 0.001241 |
| 22B02836804 | chr2 | | 234676872 | 234676872 | | UGT1A4 | | NM_007120.2 | c.1094C>T | p.Pro365Leu | EX4 | Het | missense | rs34946978 | 0.001282 | 0.002196 | 0.000077 | 0.001241 |
| **Sample ID** | | **#Chr** | **Start** | | **Stop** | | **Gene Symbol** | **Transcript** | **cHGVS** | **pHGVS** | **ExIn ID** | **Zygosity** | **Function** | **rsID** | **dbSNP Allele Freq** | **1000G AF** | **ESP6500 AF** | **GnomAD AF** |
| 22B02836804 | chr2 | | 234676872 | 234676872 | | UGT1A9 | | NM_021027.2 | c.1082C>T | p.Pro361Leu | EX4 | Het | missense | rs34946978 | 0.001282 | 0.002196 | 0.000077 | 0.001241 |
| 22B02836804 | chr2 | | 234676872 | 234676872 | | UGT1A5 | | NM_019078.1 | c.1094C>T | p.Pro365Leu | EX4 | Het | missense | rs34946978 | 0.001282 | 0.002196 | 0.000077 | 0.001241 |
| 22B02836804 | chr2 | | 234676872 | 234676872 | | UGT1A6 | | NM_001072.3 | c.1088C>T | p.Pro363Leu | EX4 | Het | missense | rs34946978 | 0.001282 | 0.002196 | 0.000077 | 0.001241 |
| 22B02836804 | chr2 | | 234676872 | 234676872 | | UGT1A8 | | NM_019076.4 | c.1082C>T | p.Pro361Leu | EX4 | Het | missense | rs34946978 | 0.001282 | 0.002196 | 0.000077 | 0.001241 |
| 22B02836804 | chr2 | | 234676872 | 234676872 | | UGT1A1 | | NM_000463.2 | c.1091C>T | p.Pro364Leu | EX4 | Het | missense | rs34946978 | 0.001282 | 0.002196 | 0.000077 | 0.001241 |
| 22B02836804 | chr2 | | 234676872 | 234676872 | | UGT1A7 | | NM_019077.2 | c.1082C>T | p.Pro361Leu | EX4 | Het | missense | rs34946978 | 0.001282 | 0.002196 | 0.000077 | 0.001241 |
| 22B02836804 | chr2 | | 234676872 | 234676872 | | UGT1A10 | | NM_019075.2 | c.1082C>T | p.Pro361Leu | EX4 | Het | missense | rs34946978 | 0.001282 | 0.002196 | 0.000077 | 0.001241 |
| 22B02836804 | chr20 | | 2841527 | 2841527 | | VPS16 | | NM_022575.2 | c.630+18G>A |  | IVS6 | Het | splice+20 | rs200732515 | 0.001125 | 0.000599 | 0.000231 | 0.001524 |
| 22B02836804 | chr20 | | 6750899 | 6750899 | | BMP2 | | NM_001200.2 | c.126A>C | p.(Ser42=) | EX2 | Het | coding-synon |  |  |  |  |  |
| 22B02836804 | chr20 | | 10621489 | 10621489 | | JAG1 | | NM_000214.2 | c.3141G>A | p.(Ser1047=) | EX25 | Het | coding-synon | rs202075581 | 0.000134 | 0.000799 |  | 0.000123 |
| 22B02836804 | chr20 | | 23346186 | 23346186 | | GZF1 | | NM_022482.3 | c.1166A>G | p.Lys389Arg | EX1 | Het | missense |  |  |  |  |  |
| 22B02836804 | chr20 | | 31374293 | 31374293 | | DNMT3B | | NM_006892.3 | c.307-15C>G |  | IVS4 | Het | splice-20 | rs150718732 | 0.000215 | 0.000399 |  | 0.000183 |
| 22B02836804 | chr20 | | 33330534 | 33330534 | | NCOA6 | | NM_014071.3 | c.3526A>G | p.Thr1176Ala | EX11 | Het | missense | rs186687743 | 0.000316 | 0.001198 | 0.000077 | 0.00031 |
| 22B02836804 | chr20 | | 47569233 | 47569233 | | ARFGEF2 | | NM_006420.2 | c.424-9C>A |  | IVS4 | Het | splice-10 |  |  |  |  |  |
| 22B02836804 | chr20 | | 48491352 | 48491352 | | SLC9A8 | | NM_001260491.1 | c.1117T>C | p.(Leu373=) | EX11 | Het | coding-synon | rs768601411 | 0.000082 |  |  | 0.000044 |
| 22B02836804 | chr20 | | 60892066 | 60892066 | | LAMA5 | | NM_005560.3 | c.7525G>T | p.Val2509Phe | EX56 | Het | missense | rs371152952 | 0.00002 |  | 0 | 0.000021 |
| 22B02836804 | chr20 | | 61451333 | 61451333 | | COL9A3 | | NM_001853.3 | c.308G>A | p.Arg103Gln | EX5 | Het | missense | rs142639450 | 0.01459 | 0.014577 | 0.009457 | 0.0136 |
| 22B02836804 | chr20 | | 62680168 | 62680168 | | SOX18 | | NM_018419.2 | c.506A>G | p.Glu169Gly | EX2E | Het | missense |  |  |  |  |  |
| 22B02836804 | chr20 | | 62680494 | 62680494 | | SOX18 | | NM_018419.2 | c.358+18G>A |  | IVS1 | Het | splice+20 | rs568521473 | 0.000091 | 0.000399 |  | 0.000039 |
| 22B02836804 | chr21 | | 28337776 | 28337776 | | ADAMTS5 | | NM_007038.3 | c.935G>T | p.Arg312Leu | EX1 | Het | missense | rs543666424 | 0.000222 | 0.000399 |  | 0.000255 |
| 22B02836804 | chr22 | | 19195771 | 19195771 | | CLTCL1 | | NM_007098.3 | c.3493C>T | p.Arg1165Cys | EX22 | Het | missense | rs190351859 | 0.005517 | 0.00639 | 0.001181 | 0.007414 |
| **Sample ID** | | **#Chr** | **Start** | | **Stop** | | **Gene Symbol** | **Transcript** | **cHGVS** | **pHGVS** | **ExIn ID** | **Zygosity** | **Function** | **rsID** | **dbSNP Allele Freq** | **1000G AF** | **ESP6500 AF** | **GnomAD AF** |
| 22B02836804 | chr22 | | 19748523 | 19748525 | | TBX1 | | NM_080647.1 | c.143_145delCGC | p.Pro48del | EX3 | Het | cds-del |  |  |  |  | 0 |
| 22B02836804 | chr22 | | 20785078 | 20785078 | | SCARF2 | | NM_153334.4 | c.972C>A | p.(Gly324=) | EX5 | Het | coding-synon | rs77962729 | 0.000878 | 0.001797 |  | 0.000862 |
| 22B02836804 | chr22 | | 27012178 | 27012178 | | CRYBB1 | | NM_001887.3 | c.106G>A | p.Gly36Ser | EX2 | Het | missense | rs144659909 | 0.000079 | 0.000399 | 0.000231 | 0.000056 |
| 22B02836804 | chr22 | | 50893151 | 50893151 | | SBF1 | | NM_002972.2 | c.4833C>T | p.(Asn1611=) | EX36 | Het | coding-synon | rs199573140 | 0.003046 | 0.001997 | 0.000746 | 0.003441 |
| 22B02836804 | chr3 | | 10417249 | 10417249 | | ATP2B2 | | NM_001001331.2 | c.1281G>A | p.(Pro427=) | EX11 | Het | coding-synon | rs113465029 | 0.000108 |  |  | 0.000108 |
| 22B02836804 | chr3 | | 14708427 | 14708427 | | CCDC174 | | NM_016474.4 | c.697G>A | p.Val233Ile | EX7 | Het | missense | rs145478230 | 0.000422 | 0.0002 | 0.000077 | 0.000433 |
| 22B02836804 | chr3 | | 15677019 | 15677019 | | BTD | | NM_000060.2 | c.133G>A | p.Gly45Arg | EX2 | Het | missense | rs34885143 | 0.009706 | 0.003794 | 0.011149 | 0.010182 |
| 22B02836804 | chr3 | | 38645235 | 38645235 | | SCN5A | | NM_198056.2 | c.1858C>T | p.Arg620Cys | EX12 | Het | missense | rs199473577 | 0.000009 |  |  | 0.000031 |
| 22B02836804 | chr3 | | 38648292 | 38648292 | | SCN5A | | NM_198056.2 | c.1008G>A | p.(Pro336=) | EX9 | Het | coding-synon | rs200285003 | 0.000252 | 0.000399 | 0.000241 | 0.00025 |
| 22B02836804 | chr3 | | 45583436 | 45583436 | | LARS2 | | NM_015340.3 | c.2520G>C | p.Gln840His | EX21 | Het | missense |  |  |  |  |  |
| 22B02836804 | chr3 | | 48621510 | 48621510 | | COL7A1 | | NM_000094.3 | c.4198-16G>A |  | IVS36 | Het | splice-20 | rs370515794 | 0.000143 | 0.0002 | 0.000154 | 0.000123 |
| 22B02836804 | chr3 | | 49136429 | 49136435 | | QARS1 | | NM_005051.1 | c.1759-13_1759-7delTCTCCTG |  | IVS18 | Het | splice-20 | rs781635166 | 0.000181 |  | 0.00024 | 0.000179 |
| 22B02836804 | chr3 | | 52378540 | 52378540 | | DNAH1 | | NM_015512.4 | c.1321G>C | p.Val441Leu | EX9 | Het | missense | rs13060192 | 0.03573 | 0.030551 | 0.03564 | 0.036747 |
| 22B02836804 | chr3 | | 52406116 | 52406116 | | DNAH1 | | NM_015512.4 | c.6666+14C>G |  | IVS42 | Het | splice+20 | rs147159692 | 0.001464 | 0.003195 | 0.000248 | 0.001282 |
| 22B02836804 | chr3 | | 52426833 | 52426838 | | DNAH1 | | NM_015512.4 | c.10279-13_10279-8delACTACA |  | IVS64 | Het | splice-20 | rs768374132 | 0.008297 |  |  | 0.003034 |
| 22B02836804 | chr3 | | 52429665 | 52429665 | | DNAH1 | | NM_015512.4 | c.11230C>T | p.Arg3744Cys | EX70 | Het | missense | rs419752 | 0.037775 | 0.029952 | 0.039314 | 0.037217 |
| 22B02836804 | chr3 | | 57132075 | 57132075 | | IL17RD | | NM_017563.3 | c.1656T>C | p.(Phe552=) | EX12 | Het | coding-synon | rs116882985 | 0.000459 | 0.001997 |  | 0.00047 |
| 22B02836804 | chr3 | | 58107156 | 58107156 | | FLNB | | NM_001457.3 | c.3052G>A | p.Val1018Met | EX20 | Het | missense | rs2276742 | 0.000325 | 0.000998 | 0.000615 | 0.000263 |
| 22B02836804 | chr3 | | 93845129 | 93845129 | | NSUN3 | | NM_022072.3 | c.818A>T | p.Gln273Leu | EX6E | Het | missense | rs779695970 | 0.00005 |  |  | 0.000044 |
| 22B02836804 | chr3 | | 119458151 | 119458151 | | MAATS1 | | NM_033364.3 | c.1511G>A | p.Arg504Gln | EX12 | Het | missense | rs375455508 | 0.000025 |  | 0.000077 | 0.000012 |
| 22B02836804 | chr3 | | 130289976 | 130289976 | | COL6A6 | | NM_001102608.1 | c.2716C>T | p.Arg906Cys | EX6 | Het | missense | rs200963433 | 0.000714 | 0.000799 |  | 0.000781 |
| **Sample ID** | | **#Chr** | **Start** | | **Stop** | | **Gene Symbol** | **Transcript** | **cHGVS** | **pHGVS** | **ExIn ID** | **Zygosity** | **Function** | **rsID** | **dbSNP Allele Freq** | **1000G AF** | **ESP6500 AF** | **GnomAD AF** |
| 22B02836804 | chr3 | | 139063005 | 139063005 | | MRPS22 | | NM_020191.2 | c.137T>G | p.Met46Arg | EX1 | Het | missense |  |  |  |  |  |
| 22B02836804 | chr3 | | 148857868 | 148857868 | | HPS3 | | NM_032383.3 | c.295A>G | p.Thr99Ala | EX2 | Het | missense |  |  |  |  |  |
| 22B02836804 | chr3 | | 148899784 | 148899784 | | CP | | NM_000096.3 | c.2554+8C>G |  | IVS14 | Het | splice+10 | rs749646388 | 0.001121 |  |  | 0.000144 |
| 22B02836804 | chr3 | | 170201230 | 170201230 | | SLC7A14 | | NM_020949.2 | c.988G>A | p.Gly330Arg | EX6 | Het | missense | rs2276717 | 0.001962 | 0.003395 | 0.000077 | 0.001726 |
| 22B02836804 | chr3 | | 184953161 | 184953161 | | EHHADH | | NM_001966.3 | c.268G>A | p.Val90Met | EX3 | Het | missense | rs56292788 | 0.000542 | 0.000998 | 0.000077 | 0.000518 |
| 22B02836804 | chr3 | | 186302391 | 186302392 | | DNAJB11 | | NM_016306.4 | c.1012+13_1012+14insTTGTGTGTGTGTGTGT |  | IVS9 | Het | splice+20 |  |  |  |  |  |
| 22B02836804 | chr3 | | 186302391 | 186302392 | | DNAJB11 | | NM_016306.4 | c.1012+13_1012+14insTTGTGTGTGTGTGT |  | IVS9 | Het | splice+20 |  |  |  |  |  |
| 22B02836804 | chr3 | | 186572419 | 186572419 | | ADIPOQ | | NM_001177800.1 | c.661C>A | p.Arg221Ser | EX4E | Het | missense | rs138773406 | 0.000239 | 0.000799 |  | 0.000207 |
| 22B02836804 | chr4 | | 5642254 | 5642254 | | EVC2 | | NM_147127.4 | c.1457G>T | p.Arg486Leu | EX10 | Het | missense |  |  |  | 0 |  |
| 22B02836804 | chr4 | | 10105534 | 10105534 | | WDR1 | | NM_017491.3 | c.215A>G | p.Tyr72Cys | EX3 | Het | missense |  |  |  |  | 0.000005 |
| 22B02836804 | chr4 | | 54966987 | 54966987 | | GSX2 | | NM_133267.2 | c.476C>G | p.Ala159Gly | EX1 | Het | missense |  |  |  |  |  |
| 22B02836804 | chr4 | | 56819340 | 56819340 | | CEP135 | | NM_025009.4 | c.203T>C | p.Leu68Ser | EX3 | Het | missense | rs147697562 | 0.000295 | 0.000799 |  | 0.000273 |
| 22B02836804 | chr4 | | 74315866 | 74315866 | | AFP | | NM_001134.1 | c.1289+16C>T |  | IVS10 | Het | splice+20 | rs201034665 | 0.00002 | 0.0002 |  | 0.000029 |
| 22B02836804 | chr4 | | 79428614 | 79428614 | | FRAS1 | | NM_025074.6 | c.9356A>G | p.Asn3119Ser | EX62 | Het | missense | rs191105001 | 0.000386 | 0.000599 | 0.000163 | 0.000327 |
| 22B02836804 | chr4 | | 96052611 | 96052611 | | BMPR1B | | NM_001203.2 | c.1024A>G | p.Lys342Glu | EX10 | Het | missense | rs748524936 | 0.000017 |  |  | 0.00002 |
| 22B02836804 | chr4 | | 100477318 | 100477318 | | TRMT10A | | NM_001134665.1 | c.480A>G | p.(Gly160=) | EX5 | Het | coding-synon |  |  |  |  |  |
| 22B02836804 | chr4 | | 122590897 | 122590898 | | ANXA5 | | NM_001154.3 | c.781-19_781-18insC |  | IVS11 | Het | splice-20 | rs781520875 | 0.000219 |  | 0.003459 | 0.000158 |
| 22B02836804 | chr4 | | 159601706 | 159601706 | | ETFDH | | NM_004453.2 | c.122G>A | p.Arg41Gln | EX2 | Het | missense | rs150105001 | 0.000058 |  | 0.000384 | 0.000064 |
| 22B02836804 | chr4 | | 185580578 | 185580578 | | PRIMPOL | | NM_152683.2 | c.265T>G | p.Tyr89Asp | EX4 | Het | missense | rs200857997 | 0.000467 | 0.001797 |  | 0.000442 |
| 22B02836804 | chr5 | | 256435 | 256436 | | SDHA | | NM_004168.2 | c.1909-14_1909-13delCT |  | IVS14 | Het | splice-20 | rs372662724 | 0.000008 |  |  | 0.000004 |
| 22B02836804 | chr5 | | 13919338 | 13919338 | | DNAH5 | | NM_001369.2 | c.922G>C | p.Asp308His | EX7 | Het | missense | rs553024984 | 0 | 0 |  | 0.000004 |
| **Sample ID** | | **#Chr** | **Start** | | **Stop** | | **Gene Symbol** | **Transcript** | **cHGVS** | **pHGVS** | **ExIn ID** | **Zygosity** | **Function** | **rsID** | **dbSNP Allele Freq** | **1000G AF** | **ESP6500 AF** | **GnomAD AF** |
| 22B02836804 | chr5 | | 37019478 | 37019478 | | NIPBL | | NM_015384.4 | c.4986C>T | p.(Asn1662=) | EX25 | Het | coding-synon | rs754312670 | 0.000041 |  |  | 0.000068 |
| 22B02836804 | chr5 | | 38490321 | 38490321 | | LIFR | | NM_002310.5 | c.2138G>A | p.Arg713His | EX15 | Het | missense | rs766002119 | 0.000033 |  |  | 0.000036 |
| 22B02836804 | chr5 | | 70238373 | 70238373 | | SMN1 | | NM_000344.3 | c.462A>G | p.(Gln154=) | EX4 | Het | coding-synon | rs4915 | 0.805 |  |  |  |
| 22B02836804 | chr5 | | 70247773 | 70247773 | | SMN1 | | NM_000344.3 | c.840C>T | p.(Phe280=) | EX8 | Het | coding-synon |  |  |  |  |  |
| 22B02836804 | chr5 | | 94872825 | 94872825 | | TTC37 | | NM_014639.3 | c.564T>C | p.(Asn188=) | EX9 | Het | coding-synon |  |  |  |  |  |
| 22B02836804 | chr5 | | 131539822 | 131539822 | | P4HA2 | | NM_004199.2 | c.1104T>C | p.(Asp368=) | EX9 | Het | coding-synon |  |  |  |  |  |
| 22B02836804 | chr5 | | 140026858 | 140026858 | | NDUFA2 | | NM_002488.4 | c.191A>G | p.Lys64Arg | EX2 | Het | missense | rs79526416 | 0.000155 | 0.000799 |  | 0.000138 |
| 22B02836804 | chr5 | | 145719315 | 145719315 | | POU4F3 | | NM_002700.2 | c.325C>T | p.His109Tyr | EX2E | Het | missense | rs754773365 | 0.000017 |  |  | 0.000012 |
| 22B02836804 | chr5 | | 150696654 | 150696654 | | SLC36A2 | | NM_181776.2 | c.1181-5T>C |  | IVS9 | Het | splice-10 | rs190486100 | 0.000572 | 0.001997 |  | 0.000512 |
| 22B02836804 | chr5 | | 150889748 | 150889748 | | FAT2 | | NM_001447.2 | c.11906-13C>A |  | IVS20 | Het | splice-20 | rs190441226 | 0.000474 | 0.001198 |  | 0.000454 |
| 22B02836804 | chr5 | | 167945076 | 167945077 | | RARS1 | | NM_002887.3 | c.1873+9_1873+10insTTTTTTTTTTTTT |  | IVS14 | Hom | splice+10 |  |  |  |  |  |
| 22B02836804 | chr6 | | 16327915 | 16327915 | | ATXN1 | | NM_000332.3 | c.627T>G | p.His209Gln | EX8 | Hom | missense | rs11969612 | 0.428571 |  |  |  |
| 22B02836804 | chr6 | | 24495286 | 24495286 | | ALDH5A1 | | NM_170740.1 | c.62G>A | p.Gly21Asp | EX1 | Het | missense | rs371923295 | 0.000423 |  |  | 0.00022 |
| 22B02836804 | chr6 | | 31238897 | 31238897 | | HLA-C | | NM_002117.5 | c.572G>C | p.Trp191Ser | EX3 | Het | missense | rs150127748 | 0.030456 |  |  | 0.014526 |
| 22B02836804 | chr6 | | 32557490 | 32557490 | | HLA-DRB1 | | NM_002124.3 | c.30C>T | p.(Ser10=) | EX1 | Het | coding-synon |  |  |  |  | 0 |
| 22B02836804 | chr6 | | 32557502 | 32557502 | | HLA-DRB1 | | NM_002124.3 | c.18C>G | p.(Leu6=) | EX1 | Het | coding-synon |  |  |  | 0 |  |
| 22B02836804 | chr6 | | 32557508 | 32557508 | | HLA-DRB1 | | NM_002124.3 | c.12G>C | p.(Leu4=) | EX1 | Het | coding-synon |  |  |  |  | 0 |
| 22B02836804 | chr6 | | 32629242 | 32629243 | | HLA-DQB1 | | NM_001243961.1 | c.662-9_662-8insTA |  | IVS3 | Het | splice-10 |  |  |  |  | 0 |
| 22B02836804 | chr6 | | 33419634 | 33419634 | | SYNGAP1 | | NM_006772.2 | c.3983G>C | p.Arg1328Pro | EX19E | Het | missense |  |  |  |  | 0.0019 |
| 22B02836804 | chr6 | | 33663494 | 33663494 | | ITPR3 | | NM_002224.3 | c.7953G>A | p.(Thr2651=) | EX58E | Het | coding-synon | rs147021135 | 0.000626 | 0.001198 | 0.000077 | 0.000585 |
| 22B02836804 | chr6 | | 45390510 | 45390510 | | RUNX2 | | NM_001024630.3 | c.239C>G | p.Ala80Gly | EX3 | Het | missense |  |  |  |  |  |
| **Sample ID** | | **#Chr** | **Start** | | **Stop** | | **Gene Symbol** | **Transcript** | **cHGVS** | **pHGVS** | **ExIn ID** | **Zygosity** | **Function** | **rsID** | **dbSNP Allele Freq** | **1000G AF** | **ESP6500 AF** | **GnomAD AF** |
| 22B02836804 | chr6 | | 45390513 | 45390513 | | RUNX2 | | NM_001024630.3 | c.242C>G | p.Ala81Gly | EX3 | Het | missense |  |  |  |  |  |
| 22B02836804 | chr6 | | 45390516 | 45390516 | | RUNX2 | | NM_001024630.3 | c.245C>G | p.Ala82Gly | EX3 | Het | missense |  |  |  |  |  |
| 22B02836804 | chr6 | | 51918923 | 51918923 | | PKHD1 | | NM_138694.3 | c.1877A>G | p.Lys626Arg | EX20 | Het | missense | rs117122807 | 0.003252 | 0.008586 |  | 0.002837 |
| 22B02836804 | chr6 | | 71003892 | 71003892 | | COL9A1 | | NM_001851.4 | c.674A>T | p.Asp225Val | EX5 | Het | missense | rs186444567 | 0.000277 | 0.000399 |  | 0.000255 |
| 22B02836804 | chr6 | | 108197875 | 108197875 | | SEC63 | | NM_007214.4 | c.1936-9delT |  | IVS18 | Het | splice-10 |  |  |  |  |  |
| 22B02836804 | chr6 | | 129511331 | 129511331 | | LAMA2 | | NM_000426.3 | c.1468-19T>C |  | IVS10 | Het | splice-20 | rs200241408 | 0.000309 | 0.001198 |  | 0.0003 |
| 22B02836804 | chr6 | | 142691874 | 142691874 | | ADGRG6 | | NM_020455.5 | c.1013A>T | p.Asn338Ile | EX4 | Het | missense | rs200437948 | 0.000303 | 0.000399 | 0.000084 | 0.000315 |
| 22B02836804 | chr6 | | 148835595 | 148835596 | | SASH1 | | NM_015278.3 | c.862+3_862+4delAA |  | IVS9 | Het | splice+10 |  |  |  |  | 0.005312 |
| 22B02836804 | chr6 | | 152708453 | 152708453 | | SYNE1 | | NM_033071.3 | c.8262G>C | p.Gln2754His | EX54 | Het | missense | rs200658991 | 0.000174 | 0.0002 |  | 0.000223 |
| 22B02836804 | chr6 | | 161807897 | 161807897 | | PRKN | | NM_004562.2 | c.1096C>T | p.Arg366Trp | EX10 | Het | missense | rs56092260 | 0.000269 | 0.001198 | 0.000308 | 0.000282 |
| 22B02836804 | chr7 | | 2962848 | 2962848 | | CARD11 | | NM_032415.4 | c.2060C>T | p.Ala687Val | EX16 | Het | missense | rs41493047 | 0.00101 | 0.000998 | 0.000846 | 0.00111 |
| 22B02836804 | chr7 | | 4827294 | 4827294 | | AP5Z1 | | NM_014855.2 | c.1341C>T | p.(Thr447=) | EX11 | Het | coding-synon | rs60284677 | 0.00047 | 0.0002 |  | 0.000334 |
| 22B02836804 | chr7 | | 8009069 | 8009069 | | GLCCI1 | | NM_138426.3 | c.88T>C | p.Ser30Pro | EX1 | Het | missense |  |  |  |  |  |
| 22B02836804 | chr7 | | 39991317 | 39991331 | | CDK13 | | NM_003718.4 | c.1077_1091delGAGCCCCTACAGTCG | p.Ser360_Arg364del | EX1 | Het | cds-del |  |  |  |  |  |
| 22B02836804 | chr7 | | 39991337 | 39991339 | | CDK13 | | NM_003718.4 | c.1097_1099delGCT | p.Arg366_Ser367delinsPro | EX1 | Het | cds-indel |  |  |  |  |  |
| 22B02836804 | chr7 | | 40234653 | 40234653 | | SUGCT | | NM_001193311.1 | c.499A>C | p.Ile167Leu | EX6 | Het | missense | rs138102615 | 0.001614 | 0.003594 |  | 0.001536 |
| 22B02836804 | chr7 | | 89938605 | 89938605 | | CFAP69 | | NM_001039706.2 | c.2579T>C | p.Ile860Thr | EX22 | Het | missense | rs758466834 | 0.000017 |  |  | 0.00004 |
| 22B02836804 | chr7 | | 117188684 | 117188684 | | CFTR | | NM_000492.3 | c.1210-11T>G |  | IVS9 | Het | splice-20 | rs73715573 | 0.010721 | 0.010383 | 0.003314 | 0.00861 |
| 22B02836804 | chr7 | | 127983715 | 127983715 | | RBM28 | | NM_018077.2 | c.118+15G>A |  | IVS1 | Het | splice+20 | rs200459017 | 0.000391 | 0.000998 |  | 0.000304 |
| 22B02836804 | chr7 | | 142457343 | 142457343 | | PRSS1 | | NM_002769.4 | c.8C>T | p.Pro3Leu | EX1 | Het | missense | rs374597855 | 0.000025 |  |  | 0 |
| 22B02836804 | chr7 | | 142457347 | 142457347 | | PRSS1 | | NM_002769.4 | c.12C>T | p.(Leu4=) | EX1 | Het | coding-synon | rs749968829 | 0.000025 |  |  | 0 |
| **Sample ID** | | **#Chr** | **Start** | | **Stop** | | **Gene Symbol** | **Transcript** | **cHGVS** | **pHGVS** | **ExIn ID** | **Zygosity** | **Function** | **rsID** | **dbSNP Allele Freq** | **1000G AF** | **ESP6500 AF** | **GnomAD AF** |
| 22B02836804 | chr7 | | 142457365 | 142457365 | | PRSS1 | | NM_002769.4 | c.30G>T | p.(Val10=) | EX1 | Het | coding-synon | rs779260304 | 0.000008 |  |  |  |
| 22B02836804 | chr7 | | 142457375 | 142457375 | | PRSS1 | | NM_002769.4 | c.40C>G | p.Leu14Val | EX1 | Het | missense | rs747228052 | 0.000008 |  |  | 0.000004 |
| 22B02836804 | chr7 | | 142457382 | 142457382 | | PRSS1 | | NM_002769.4 | c.40+7A>T |  | IVS1 | Het | splice+10 | rs796173487 | 0 |  |  |  |
| 22B02836804 | chr7 | | 142457386 | 142457386 | | PRSS1 | | NM_002769.4 | c.40+11T>C |  | IVS1 | Het | splice+20 | rs761465973 | 0.000008 |  |  | 0.000004 |
| 22B02836804 | chr7 | | 142458412 | 142458412 | | PRSS1 | | NM_002769.4 | c.47C>T | p.Ala16Val | EX2 | Het | missense | rs202003805 | 0.016039 |  |  | 0.00009 |
| 22B02836804 | chr7 | | 142458486 | 142458486 | | PRSS1 | | NM_002769.4 | c.121C>T | p.(Leu41=) | EX2 | Het | coding-synon | rs369646357 | 0.003751 |  |  | 0.000115 |
| 22B02836804 | chr7 | | 142459894 | 142459894 | | PRSS1 | | NM_002769.4 | c.454+16A>T |  | IVS3 | Het | splice+20 | rs377570765 | 0.000133 |  |  | 0.00002 |
| 22B02836804 | chr7 | | 142460379 | 142460379 | | PRSS1 | | NM_002769.4 | c.552C>T | p.(Phe184=) | EX4 | Het | coding-synon | rs767583768 | 0.007919 |  |  | 0 |
| 22B02836804 | chr7 | | 142460388 | 142460388 | | PRSS1 | | NM_002769.4 | c.561C>T | p.(Gly187=) | EX4 | Het | coding-synon | rs1804561 | 0.009654 |  |  | 0.000012 |
| 22B02836804 | chr7 | | 142460764 | 142460764 | | PRSS1 | | NM_002769.4 | c.637G>A | p.Val213Ile | EX5E | Het | missense | rs200902389 | 0.003234 |  |  | 0.000016 |
| 22B02836804 | chr7 | | 142460778 | 142460778 | | PRSS1 | | NM_002769.4 | c.651T>C | p.(Gly217=) | EX5E | Het | coding-synon | rs562372415 | 0.000079 | 0.0002 |  | 0.00004 |
| 22B02836804 | chr7 | | 142460779 | 142460779 | | PRSS1 | | NM_002769.4 | c.652G>T | p.Asp218Tyr | EX5E | Het | missense | rs574391339 | 0.000079 | 0.0002 |  | 0.00004 |
| 22B02836804 | chr7 | | 144095496 | 144095496 | | NOBOX | | NM_001080413.3 | c.1653T>C | p.(Leu551=) | EX9 | Het | coding-synon |  |  |  |  | 0.000035 |
| 22B02836804 | chr7 | | 150883497 | 150883497 | | ASB10 | | NM_080871.3 | c.521G>A | p.Arg174Gln | EX2 | Het | missense |  |  |  |  | 0.00001 |
| 22B02836804 | chr7 | | 151478406 | 151478406 | | PRKAG2 | | NM_016203.3 | c.298G>A | p.Gly100Ser | EX3 | Het | missense | rs79474211 | 0.008392 | 0.014577 | 0.000846 | 0.00736 |
| 22B02836804 | chr7 | | 151875096 | 151875097 | | KMT2C | | NM_170606.2 | c.7443-2_7443-1insA |  | IVS37 | Het | splice-3 | rs753425356 | 0.985332 |  |  | 0.000016 |
| 22B02836804 | chr7 | | 151875100 | 151875101 | | KMT2C | | NM_170606.2 | c.7443-6_7443-5insTTTTTTTTTA |  | IVS37 | Het | splice-10 |  |  |  |  |  |
| 22B02836804 | chr7 | | 151945228 | 151945228 | | KMT2C | | NM_170606.2 | c.2291C>T | p.Ser764Phe | EX14 | Het | missense | rs200184971 | 0.001076 |  |  | 0.000032 |
| 22B02836804 | chr7 | | 156802643 | 156802644 | | MNX1 | | NM_005515.3 | c.401_402insCGCCGCCGC | p.Ala134_Gly135insAlaAlaAla | EX1 | Hom | cds-ins |  |  |  |  |  |
| 22B02836804 | chr8 | | 1873490 | 1873490 | | ARHGEF10 | | NM_014629.2 | c.2530G>A | p.Gly844Arg | EX22 | Het | missense |  |  |  |  |  |
| 22B02836804 | chr8 | | 6302457 | 6302457 | | MCPH1 | | NM_024596.3 | c.1214T>C | p.Leu405Pro | EX8 | Het | missense | rs556803400 | 0.000589 | 0.000799 |  | 0.000665 |
| **Sample ID** | | **#Chr** | **Start** | | **Stop** | | **Gene Symbol** | **Transcript** | **cHGVS** | **pHGVS** | **ExIn ID** | **Zygosity** | **Function** | **rsID** | **dbSNP Allele Freq** | **1000G AF** | **ESP6500 AF** | **GnomAD AF** |
| 22B02836804 | chr8 | | 8749306 | 8749306 | | MFHAS1 | | NM_004225.2 | c.1263A>G | p.(Gly421=) | EX1 | Het | coding-synon | rs746106122 | 0.000008 |  |  | 0.000012 |
| 22B02836804 | chr8 | | 10466476 | 10466476 | | RP1L1 | | NM_178857.5 | c.5132G>C | p.Gly1711Ala | EX4E | Het | missense | rs200635063 | 0.000517 | 0.001198 |  | 0.000493 |
| 22B02836804 | chr8 | | 10467589 | 10467590 | | RP1L1 | | NM_178857.5 | c.4018_4019insGGACTAAAGTAATAGAAGGGCTGCAAGAAGAGAGGGTGCAGTTAGAGG | p.Glu1339_Glu1340insGlyThrLysValIleGluGlyLeuGlnGluGluArgValGlnLeuGlu | EX4E | Het | cds-ins |  |  |  |  |  |
| 22B02836804 | chr8 | | 10467637 | 10467637 | | RP1L1 | | NM_178857.5 | c.3971A>G | p.Glu1324Gly | EX4E | Het | missense |  |  |  |  |  |
| 22B02836804 | chr8 | | 10469386 | 10469386 | | RP1L1 | | NM_178857.5 | c.2222C>A | p.Thr741Asn | EX4E | Het | missense |  |  |  |  | 0.000004 |
| 22B02836804 | chr8 | | 10470838 | 10470838 | | RP1L1 | | NM_178857.5 | c.770C>G | p.Thr257Ser | EX4E | Het | missense |  |  |  |  | 0.000004 |
| 22B02836804 | chr8 | | 15978126 | 15978134 | | MSR1 | | NM_138715.2 | c.1034-12_1034-11delTT |  | IVS8 | Het | splice-20 |  |  |  |  |  |
| 22B02836804 | chr8 | | 20068093 | 20068093 | | ATP6V1B2 | | NM_001693.3 | c.399T>C | p.(Asn133=) | EX5 | Het | coding-synon |  |  |  |  |  |
| 22B02836804 | chr8 | | 21984575 | 21984575 | | HR | | NM_005144.4 | c.1380C>T | p.(Asp460=) | EX3 | Het | coding-synon | rs147308644 | 0.004607 | 0.006989 | 0.000236 | 0.005214 |
| 22B02836804 | chr8 | | 30700014 | 30700014 | | TEX15 | | NM_031271.3 | c.6520A>G | p.Lys2174Glu | EX1 | Het | missense | rs142315341 | 0.001806 | 0.003994 | 0.000077 | 0.002379 |
| 22B02836804 | chr8 | | 30700983 | 30700983 | | TEX15 | | NM_031271.3 | c.5551A>G | p.Lys1851Glu | EX1 | Het | missense |  |  |  |  |  |
| 22B02836804 | chr8 | | 48691059 | 48691059 | | PRKDC | | NM_006904.6 | c.11814C>T | p.(Ile3938=) | EX83 | Het | coding-synon | rs750761283 | 0.000018 |  |  | 0.000016 |
| 22B02836804 | chr8 | | 87588047 | 87588047 | | CNGB3 | | NM_019098.4 | c.2415A>C | p.Glu805Asp | EX18E | Het | missense | rs186448979 | 0.001158 | 0.003594 |  | 0.001075 |
| 22B02836804 | chr8 | | 87641127 | 87641127 | | CNGB3 | | NM_019098.4 | c.1480+20G>A |  | IVS12 | Het | splice+20 | rs117375929 | 0.000222 | 0.001597 |  | 0.000203 |
| 22B02836804 | chr8 | | 118825226 | 118825226 | | EXT1 | | NM_000127.2 | c.1633-26C>A |  | IVS7 | Het | intron | rs188609829 | 0.003338 | 0.004193 | 0.000154 | 0.002856 |
| 22B02836804 | chr8 | | 133877623 | 133877623 | | TG | | NM_003235.4 | c.-1623A>G |  |  | Het | promoter | rs180195 | 0.592452 | 0.592452 |  |  |
| 22B02836804 | chr8 | | 144990353 | 144990353 | | PLEC | | NM_000445.3 | c.13717G>A | p.Val4573Met | EX33E | Het | missense | rs573424409 | 0.000599 | 0.000599 |  | 0.000178 |
| 22B02836804 | chr8 | | 144994778 | 144994778 | | PLEC | | NM_000445.3 | c.9292G>A | p.Ala3098Thr | EX33E | Het | missense | rs782624968 | 0.000018 |  |  | 0.000008 |
| 22B02836804 | chr8 | | 145009203 | 145009203 | | PLEC | | NM_000445.3 | c.882C>T | p.(Asp294=) | EX9 | Het | coding-synon | rs202218097 | 0.000549 | 0.001198 | 0.000159 | 0.000597 |
| **Sample ID** | | **#Chr** | **Start** | | **Stop** | | **Gene Symbol** | **Transcript** | **cHGVS** | **pHGVS** | **ExIn ID** | **Zygosity** | **Function** | **rsID** | **dbSNP Allele Freq** | **1000G AF** | **ESP6500 AF** | **GnomAD AF** |
| 22B02836804 | chr9 | | 2719083 | 2719083 | | KCNV2 | | NM_133497.3 | c.1344G>C | p.Trp448Cys | EX1 | Het | missense | rs143382624 | 0.000383 | 0.000799 |  | 0.000357 |
| 22B02836804 | chr9 | | 13176290 | 13176290 | | MPDZ | | NM_003829.4 | c.2776G>A | p.Ala926Thr | EX20 | Het | missense | rs144992780 | 0.000363 | 0.000998 |  | 0.000202 |
| 22B02836804 | chr9 | | 16436068 | 16436068 | | BNC2 | | NM_017637.5 | c.2124C>T | p.(Ala708=) | EX6 | Het | coding-synon | rs145645768 | 0.000309 | 0.000998 |  | 0.000326 |
| 22B02836804 | chr9 | | 34512388 | 34512388 | | DNAI1 | | NM_012144.2 | c.1455G>A | p.(Thr485=) | EX15 | Het | coding-synon | rs200766993 | 0.000072 | 0.0002 |  | 0.000044 |
| 22B02836804 | chr9 | | 35068323 | 35068323 | | VCP | | NM_007126.3 | c.54A>G | p.(Lys18=) | EX2 | Het | coding-synon | rs766042571 | 0.00014 |  |  | 0.000159 |
| 22B02836804 | chr9 | | 35077114 | 35077114 | | FANCG | | NM_004629.1 | c.647-16C>A |  | IVS5 | Het | splice-20 | rs200107462 | 0.001171 | 0.001797 | 0.000077 | 0.001006 |
| 22B02836804 | chr9 | | 95237025 | 95237030 | | ASPN | | NM_017680.4 | c.147_152delTGATGA | p.Asp49_Asp50del | EX2 | Het | cds-del |  |  |  |  |  |
| 22B02836804 | chr9 | | 133556993 | 133557007 | | PRDM12 | | NM_021619.2 | c.1062_1076delCGCCGCCGCCGCCGC | p.Ala355_Ala359del | EX5E | Het | cds-del |  |  |  |  | 0 |
| 22B02836804 | chr9 | | 138589474 | 138589474 | | SOHLH1 | | NM_001012415.2 | c.346-1G>A |  | IVS3 | Het | splice-3 | rs140132974 | 0.003279 | 0.002995 | 0.000308 | 0.002897 |
| 22B02836804 | chr9 | | 138589474 | 138589474 | | KCNT1 | | NM_020822.2 | c.-4631C>T |  |  | Het | promoter | rs140132974 | 0.003279 | 0.002995 | 0.000308 | 0.002897 |
| 22B02836804 | chr9 | | 139311556 | 139311556 | | PMPCA | | NM_015160.1 | c.787C>G | p.Leu263Val | EX7 | Het | missense |  |  |  |  |  |
| 22B02836804 | chr9 | | 139329234 | 139329234 | | INPP5E | | NM_019892.4 | c.894C>T | p.(Asn298=) | EX2 | Het | coding-synon |  |  |  |  | 0.000032 |
| 22B02836804 | chr9 | | 139905532 | 139905532 | | ABCA2 | | NM_212533.2 | c.6039G>T | p.(Pro2013=) | EX39 | Het | coding-synon | rs200252232 | 0.000704 | 0.000399 | 0.000163 | 0.00061 |
| 22B02836804 | chrX | | 9656196 | 9656196 | | TBL1X | | NM_001139466.1 | c.497C>G | p.Ala166Gly | EX7 | Het | missense |  |  |  |  |  |
| 22B02836804 | chrX | | 38145910 | 38145910 | | RPGR | | NM_001034853.1 | c.2342C>G | p.Ala781Gly | EX15E | Het | missense |  |  |  | 0 |  |
| 22B02836804 | chrX | | 54275207 | 54275207 | | WNK3 | | NM_020922.4 | c.3574A>G | p.Ser1192Gly | EX17 | Hemi | missense |  |  |  |  |  |
| 22B02836804 | chrX | | 55051235 | 55051235 | | ALAS2 | | NM_000032.4 | c.220C>T | p.(Leu74=) | EX3 | Hemi | coding-synon | rs200307584 | 0.000404 | 0.00053 |  | 0.000414 |
| 22B02836804 | chrX | | 73811761 | 73811761 | | RLIM | | NM_016120.3 | c.1389C>A | p.(Ser463=) | EX4E | Het | coding-synon | rs773922290 | 0 |  |  | 0.000202 |
| 22B02836804 | chrX | | 74376092 | 74376092 | | ABCB7 | | NM_004299.3 | c.16A>G | p.Met6Val | EX1 | Hemi | missense |  |  |  |  | 0.000006 |
| 22B02836804 | chrX | | 76939281 | 76939281 | | ATRX | | NM_000489.3 | c.1467C>T | p.(Thr489=) | EX9 | Hemi | coding-synon | rs199929884 | 0.00024 | 0.000265 |  | 0.000312 |
| 22B02836804 | chrX | | 118985711 | 118985712 | | UPF3B | | NM_080632.2 | c.263+18_263+19insAAAAAAAAAA |  | IVS2 | Het | splice+20 |  |  |  |  |  |
| **Sample ID** | | **#Chr** | **Start** | | **Stop** | | **Gene Symbol** | **Transcript** | **cHGVS** | **pHGVS** | **ExIn ID** | **Zygosity** | **Function** | **rsID** | **dbSNP Allele Freq** | **1000G AF** | **ESP6500 AF** | **GnomAD AF** |
| 22B02836804 | chrX | | 119077459 | 119077459 | | NKAP | | NM_024528.3 | c.110C>T | p.Pro37Leu | EX1 | Hemi | missense |  |  |  |  | 0.000006 |
| 22B02836804 | chrX | | 153008434 | 153008434 | | ABCD1 | | NM_000033.3 | c.1781-7C>T |  | IVS7 | Het | splice-10 | rs79915675 | 0.000166 |  |  | 0.000021 |
| 22B02836804 | chrX | | 153008476 | 153008476 | | ABCD1 | | NM_000033.3 | c.1816T>C | p.Ser606Pro | EX8 | Het | missense | rs201774661 | 0.008522 |  |  | 0.000381 |
| 22B02836804 | chrX | | 153008483 | 153008483 | | ABCD1 | | NM_000033.3 | c.1823G>A | p.Gly608Asp | EX8 | Het | missense | rs78993751 | 0.008355 |  |  | 0.000409 |
| 22B02836804 | chrX | | 153008537 | 153008537 | | ABCD1 | | NM_000033.3 | c.1865+12G>A |  | IVS8 | Het | splice+20 | rs373638861 | 0.000279 |  |  | 0.000059 |
| 22B02836804 | chrX | | 153008542 | 153008542 | | ABCD1 | | NM_000033.3 | c.1865+17G>A |  | IVS8 | Het | splice+20 | rs377149542 | 0.00025 |  |  | 0.000022 |
